# Novel oxindole/benzofuran hybrids as potential dual CDK2/GSK-3β inhibitors targeting breast cancer: design, synthesis, biological evaluation, and *in silico* studies

**DOI:** 10.1080/14756366.2020.1862101

**Published:** 2020-12-16

**Authors:** Wagdy M. Eldehna, Sara T. Al-Rashood, Tarfah Al-Warhi, Razan O. Eskandrani, Amal Alharbi, Ahmed M. El Kerdawy

**Affiliations:** a Department of Pharmaceutical Chemistry, Faculty of Pharmacy, Kafrelsheikh University, Kafr El-Sheikh, Egypt; b Department of Pharmaceutical Chemistry, College of Pharmacy, King Saud University, Riyadh, Saudi Arabia; c Department of Chemistry, College of Science, Princess Nourah Bint Abdulrahman University, Riyadh, Saudi Arabia; d Department of Pharmaceutical Chemistry, Faculty of Pharmacy, Cairo University, Cairo, Egypt; e Department of Pharmaceutical Chemistry, Faculty of Pharmacy, New Giza University, Cairo, Egypt

**Keywords:** Dual kinase inhibitors, CDK2/GSK-3β inhibitors, Benzofuran-2-carbohydrazide, Isatin, anticancer agents

## Abstract

The serine/threonine protein kinases CDK2 and GSK-3β are key oncotargets in breast cancer cell lines, therefore, in the present study three series of oxindole-benzofuran hybrids were designed and synthesised as dual CDK2/GSK-3β inhibitors targeting breast cancer (**5a–g**, **7a–h**, and **13a–b**). The *N^1^*-unsubstituted oxindole derivatives, **series 5**, showed moderate to potent activity on both MCF-7 and T-47D breast cancer cell lines. Compounds **5d–f** showed the most potent cytotoxic activity with IC_50_ of 3.41, 3.45 and 2.27 μM, respectively, on MCF-7 and of 3.82, 4.53 and 7.80 μM, respectively, on T-47D cell lines, in comparison to the used reference standard (staurosporine) IC_50_ of 4.81 and 4.34 μM, respectively. On the other hand, the *N^1^*-substituted oxindole derivatives, **series 7** and **13**, showed moderate to weak cytotoxic activity on both breast cancer cell lines. CDK2 and GSK-3β enzyme inhibition assay of **series 5** revealed that compounds **5d** and **5f** are showing potent dual CDK2/GSK-3β inhibitory activity with IC_50_ of 37.77 and 52.75 nM, respectively, on CDK2 and 32.09 and 40.13 nM, respectively, on GSK-3β. The most potent compounds **5d–f** caused cell cycle arrest in the G2/M phase in MCF-7 cells inducing cell apoptosis because of the CDK2/GSK-3β inhibition. Molecular docking studies showed that the newly synthesised *N^1^*-unsubstituted oxindole hybrids have comparable binding patterns in both CDK2 and GSK-3β. The oxindole ring is accommodated in the hinge region interacting through hydrogen bonding with the backbone CO and NH of the key amino acids Glu81 and Leu83, respectively, in CDK2 and Asp133 and Val135, respectively, in GSK-3β. Whereas, in series **7** and **13**, the *N^1^*-substitutions on the oxindole nucleus hinder the compounds from achieving these key interactions with hinge region amino acids what rationalises their moderate to low anti-proliferative activity.

## 1. Introduction

Protein kinases (PKs) represent the fifth largest human protein family comprising 518 proteins^1–2^. They are key regulators of cell functions through cellular signalling modulation and complex biological functions coordination such as cell growth, differentiation, proliferation, metabolism, migration, and apoptosis (programmed cell death)^1^^,^^3–5^. PKs exert their functions through catalysing the transfer of the gamma-phosphate group of an ATP molecule onto a substrate protein hydroxyl group (substrate protein phosphorylation) accomplishing cellular signals transduction and amplification^1^. According to the phosphorylated residue in the substrate protein, PKs are classified into tyrosine kinases (90 kinases) and serine/threonine kinases (385 kinases), with a small group of dual specificity kinases including MEK1 and MEK2 which could catalyse the phosphorylation of both tyrosine and threonine on target proteins^6–7^.

The prominent and critical importance of PKs dictates strict regulation of their cellular levels and activities, therefore, PKs dysregulation contributes to several diseases such as cancer, metabolic disorders (such as diabetes), cardiovascular diseases, neurodegenerative diseases (such as Alzheimer’s disease), inflammatory disorders and autoimmune diseases (such as rheumatoid arthritis)^8^. The usage of PKs inhibitors is a promising strategy to manage their dysregulation in these diseases^8–11^. In cancer treatment, PK inhibitors represent a key class of targeted chemotherapy which is devoid of the common side effects of conventional cancer chemotherapy because they target cancer cells’ signalling pathways and microenvironment with minimum undesirable effects on normal cells^12–15^.

Breast cancer is a very common cancer type all over the world, and despite of its possible early detection by advanced diagnostic techniques, breast cancer is still considered the leading cause of death among women worldwide^16–17^. Thus, there is a serious continuous demand for the discovery of more effective anti-breast cancer agents. Several studies reported the overexpression of several PKs in primary as well as in metastatic breast cancers such as vascular endothelial growth factor receptor (VEGFR)^18–20^, fibroblast growth factor receptor (FGFR)^20–21^, platelet-derived growth factor receptor (PDGFR)^20^^,^^22^, cyclin dependent kinases (CDKs) and their activating cyclins^23–27^, and glycogen synthase kinase 3β (GSK-3β)^28–30^ and references therein.

**Figure 1.**
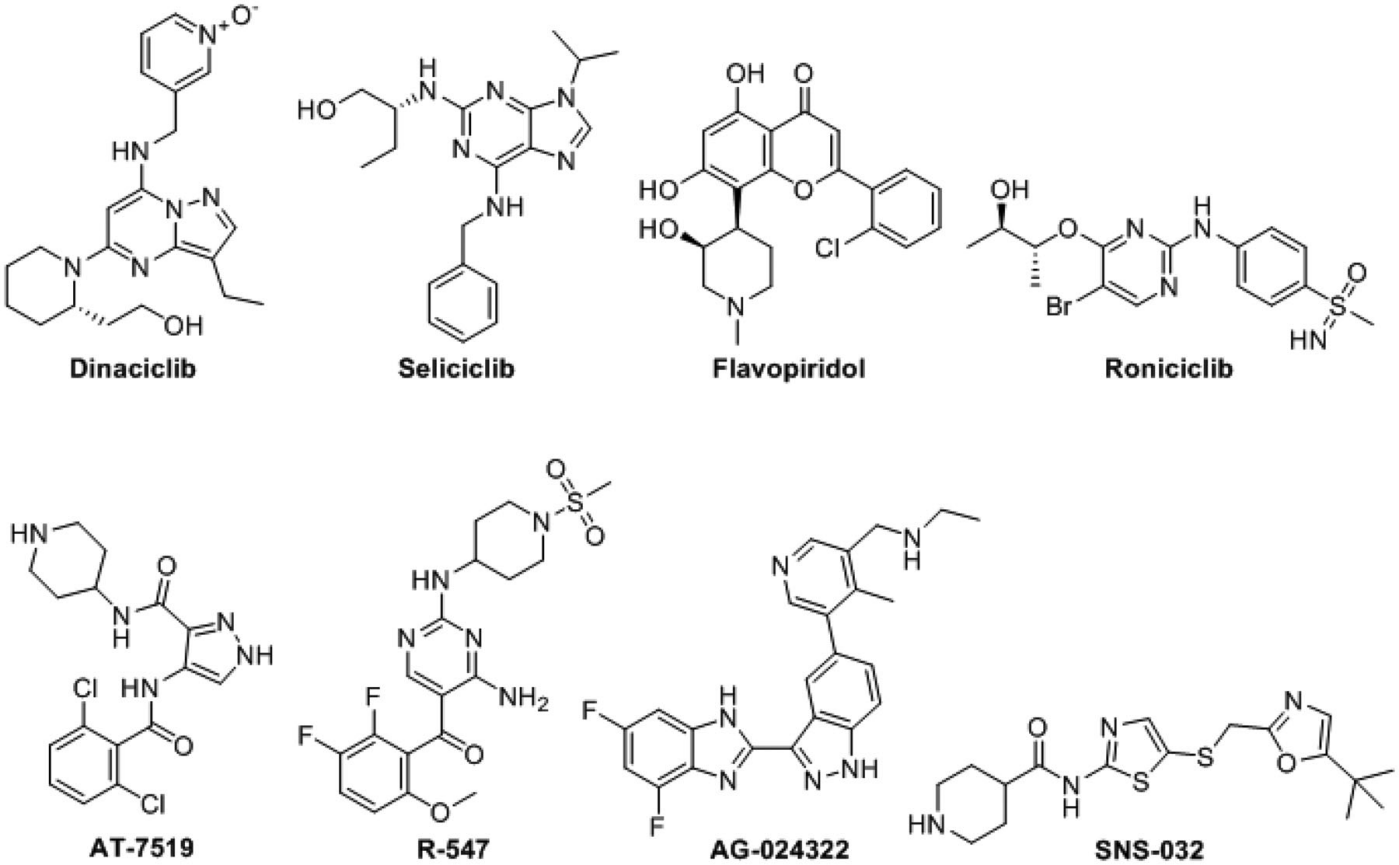
CDKs inhibitors in the clinical trial phases.

CDKs, a family of serine/threonine protein kinases, are involved in several cellular functions such as division, proliferation, apoptosis, and gene transcription. CDK1, CDK2, CDK4 and CDK6 subtypes are responsible for the regulation of cell-cycle progression in its different phases, moreover, they play a pivotal role in cancer cell continuous proliferation^31–33^. CDK2 subtype, specifically, received a great attention as a therapeutic target for cancer treatment due to its key role in several cellular processes upon complexation with its activating cognate, cyclin A or E, in addition, dysregulation of CDK2 or its cyclin partners was detected in various cancers such as ovarian, lung, pancreatic carcinomas, melanoma as well as breast cancer ^23–27^^,^^34–37^. The impact of CDK2 inhibition on cancer cells confirmed the validity of CDK2 as a promising anticancer drug target, therefore, many CDK2 inhibitors have been progressed through clinical trials (Figure 1)^34^^,^^35^^,^^38^. In particular, CDK2 inhibition was found to effectively hinder breast cancer cells proliferation including those resistant to hormonal therapy^39–41^.

GSK-3, another serine/threonine protein kinase and an essential element of the WNT signalling pathway, contributes to several physiological processes ranging from gene expression to glycogen metabolism^42^. GSK-3 has two isozymes α and β encoded by two independent GSK-3 genes^43–48^. GSK-3β is known as the “multi-tasking kinase” due to its participation in several signalling pathways^49^. Dysregulated activity of GSK-3β is associated with several diseases such as type 2 diabetes, heart disease, chronic inflammatory diseases, neurodegenerative diseases, and cancer, therefore, GSK-3β inhibition is a potential approach for the treatment of these diseases^30^^,^^50^. GSK-3β is overexpressed in colon, pancreatic as well as breast cancers as it plays a key role in cancer cell proliferation and survival^28^^,^^29^^,^^51^. The overexpression of GSK-3β correlates with poor prognosis in patients with breast cancer^28–29^, moreover, aberrant nuclear accumulation of GSK-3β in five human breast cancer cell lines and in 70% of human breast carcinomas was reported^29^, furthermore, GSK-3β inhibition suppressed the viability of breast cancer cells *in vitro*, moreover, GSK-3β inhibition overcomes chemoresistance in human breast cancer^52^.

The complex nature of cancer mandates the implementation of multitarget treatment strategies^53–55^. Moreover, in several cancer types especially solid tumours, such as breast cancer, more than one PK is upregulated and contributes to carcinogenesis^54^^,^^56^. Furthermore, drug resistance is commonly developed by target PK mutation, target PK amplification/overexpression, or through upregulation of alternative/downstream pathways^54^. Thus, the use of multiple PK inhibitors is an emerging and appealing trend to overcome cancer development, progression, and resistance. A possible drawback of this approach is the pharmacokinetics (ADME) interference among the co-administered inhibitors, another concern is the potential toxicity of the used combination. To avoid these drawbacks, the design of small-molecules that are intentionally tailored to target more than one PK (multikinase inhibitors) was considered^53–55^^,^^57^^,^^58^. Multikinase inhibitors with favourable selectivity or multitarget selectivity (poly-specific inhibitors) might be more suitable for cancer treatment to balance efficacy and toxicity^53–55^^,^^57^^,^^58^.

Dual CDKs/GSK-3β inhibition is a promising therapeutic approach to confront uncontrolled cancer cell proliferation^35^^,^^36^^,^^51^^,^^59–66^. In particular, CDK2 and GSK-3β share a high homologous sequence (33% amino acid identity) and are used as examples for the homological CMGC protein kinase family^59^^,^^64^^,^^67–75^. Therefore, designing dual CDK2/GSK-3β inhibitors as anticancer agents is an amenable and attractive strategy^65–66^.

**Figure 2.**
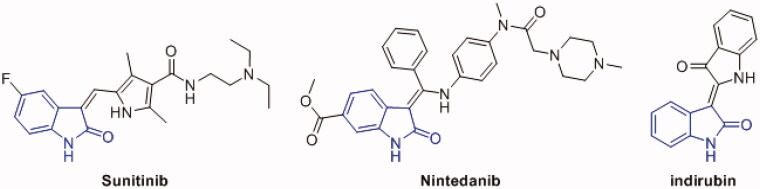
Oxindole derivatives with multikinase activity.

1*H*-indole-2,3-dione (isatin) is a privileged scaffold that emerged as a promising nucleus in medicinal chemistry demonstrating a broad range of pharmacological activities including antibacterial, anticonvulsant, antifungal, antiviral, as well as anticancer activity^76–78^. Isatin derivatives exert their anticancer activity through several mechanisms such as inhibition and/or modulation of proteases, translation initiation, angiogenesis or tubulin polymerisation, moreover, PK inhibition is one of the key anticancer mechanisms of isatin derivatives^76–78^. Several oxindole-based multikinase inhibitors (Figure 2) have been approved for cancer treatment such as sunitinib (2006) for gastrointestinal stromal tumour and renal cell carcinoma as PDGFR and VEGFR inhibitor^79^, and nintedanib (2014) for idiopathic pulmonary fibrosis as FGFR, PDGFR and VEGFR inhibitor^80^. Moreover, several oxindole derivatives have been designed and synthesised as inhibitors for diverse PKs such as FLT3 kinase^81^, VEGFR^82–83^, polo-like kinase 4 (PLK4)^84^, aurora B kinase^85^, p90 ribosomal S6 protein kinase 2 (RSK2)^86^, microtubule affinity-regulating kinase 4 (MARK4)^87^ as well as CDKs^78^^,^^88^, and GSK-3β^59^.

The naturally occurring oxindole derivative, indirubin, (Figure 2) has been identified as the main active ingredient of the traditional Chinese medicinal remedy, *Dang Gui Long Hui Wan* which is used to treat chronic myelocytic leukaemia (CML)^64^^,^^66^^,^^89^. Indirubin and its derivatives showed distinctive antiproliferative effect which is attributed to their inhibition of CDKs and GSK-3 through competing with ATP at the kinase domain^74^^,^^90–93^.

**Figure 3.**
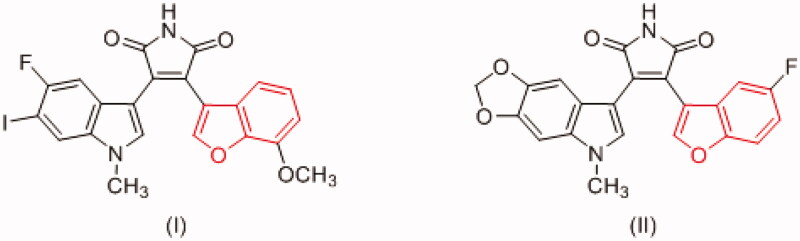
Benzofuran derivatives with GSK-3β inhibitory activity.

Benzofuran is another privileged scaffold that demonstrated several desirable biological activities among which are analgesic, anti-inflammatory, antipyretic, antibacterial, antifungal, antiviral, antihyperglycemic, anti-hyperlipidemic, anti-oxidant, as well as anticancer activities^94–96^. Benzofuran derivatives mediate their anticancer activity through several mechanism such as farnesyltransferase, angiogenesis, oestrogen receptor, human peptide deformylase, tubulin polymerisation, and carbonic anhydrase inhibition^94^^,^^97^ and reference therein. Moreover, some benzofuran derivatives exert their anticancer activity through PK inhibition such as Pim-1^98^, mTOR signaling^99–100^, Src kinase^101^, as well as GSK-3β^51^ (Figure 3).

**Figure 4.**
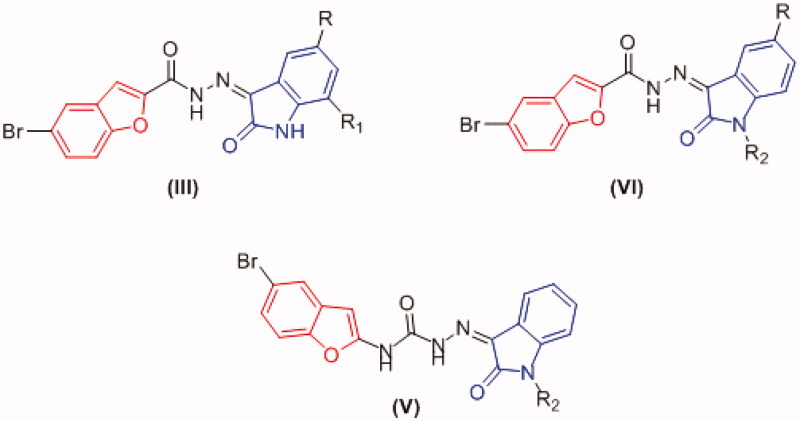
Designed oxindole/benzofuran dual CDK2/GSK-3β hybrid inhibitors.

Based on the reported PK inhibitory activity of isatin and benzofuran nuclei, specially, on the homologous CDK2 and GSK-3β kinases, a hybridisation strategy of these two privileged scaffolds was adapted to design dual CDK2/GSK-3β hybrid inhibitors which could target breast cancer. For the hybridisation strategy, the bioisosteric amido and uriedo moieties were used as linkers between the two nuclei, moreover, different substitution pattern and nature on the oxindole ring offering various electronic and lipophilic environments were introduced to study their impact on the activity (Figure 4). On account of the hydrophobic nature of the CDK2 binding site, it was anticipated that the grafting a hydrophobic substituent (such as Br) within the benzofuran motif could achieve a plenty of hydrophobic interactions.

The newly synthesised compounds were experimentally evaluated for their anti-proliferative activities against breast cancer cell lines T-47D and MCF-7. Compounds showed promising anti-proliferative activity were tested for their potential dual CDK2/GSK-3β inhibitory activity, moreover, compounds showed prominent anti-proliferative activity and *in vitro* PK inhibitory activity were investigated further by studying their effect on cell cycle progression and cell apoptosis. Furthermore, molecular docking studies were also performed to study the interaction of the newly synthesised hybrid compounds with CDK2 and GSK-3β kinase domain hot spots (key amino acids) to rationalise their biological activity and to reveal their probable binding mode.

## 2. Results and discussion

### 2.1. Chemistry

**Scheme 1.**
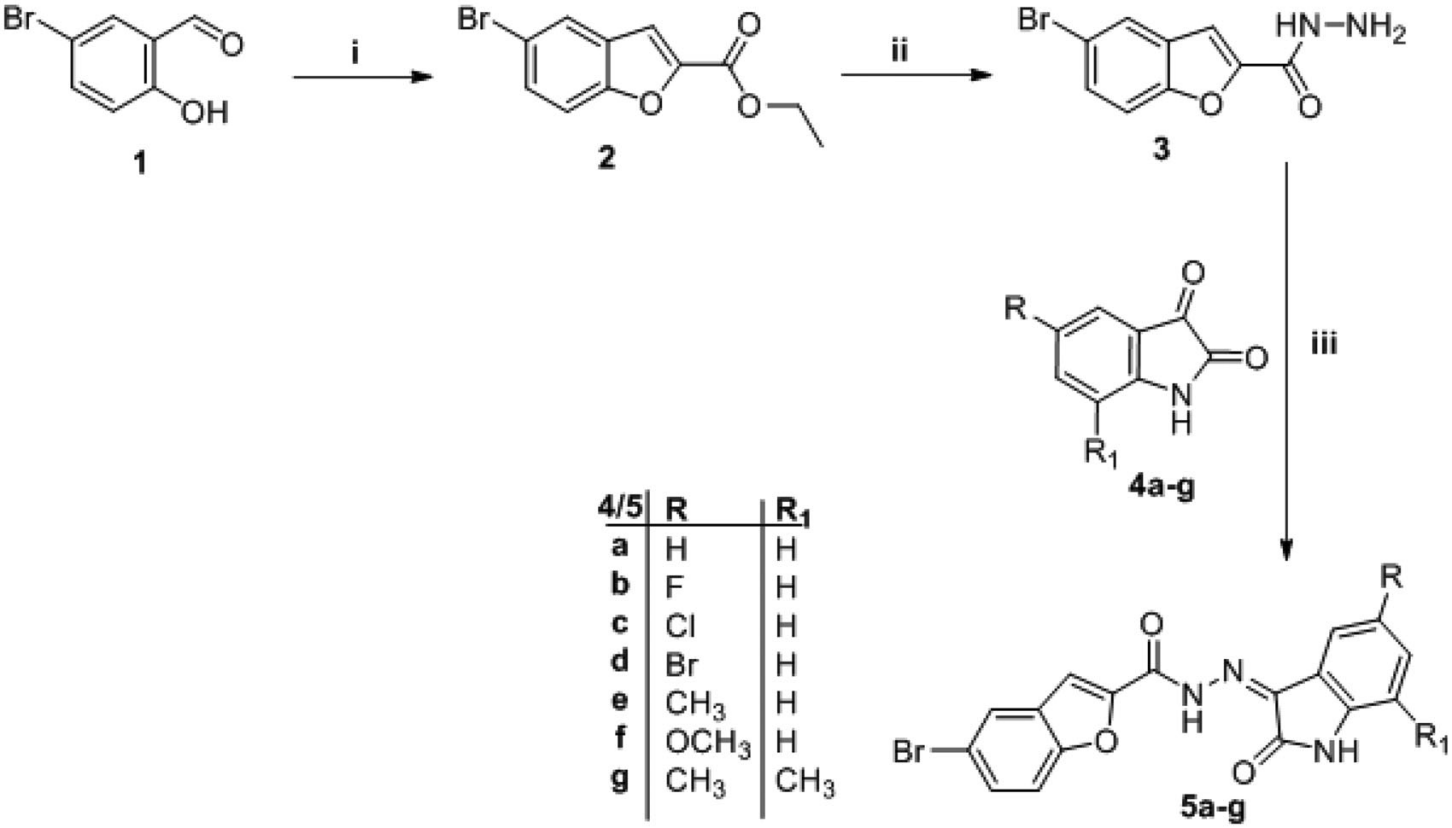
Synthesis of target compounds **5a–g**; Reagents and conditions: (**i**) Ethyl bromoacetate/Acetonitrile/K_2_CO_3_/reflux 4 h, (**ii**) NH_2_NH_2_.H_2_O/methanol/reflux 3 h, (**iii**) Ethanol/glacial acetic acid (Cat.)/reflux 4–7 h.

**Scheme 2.**
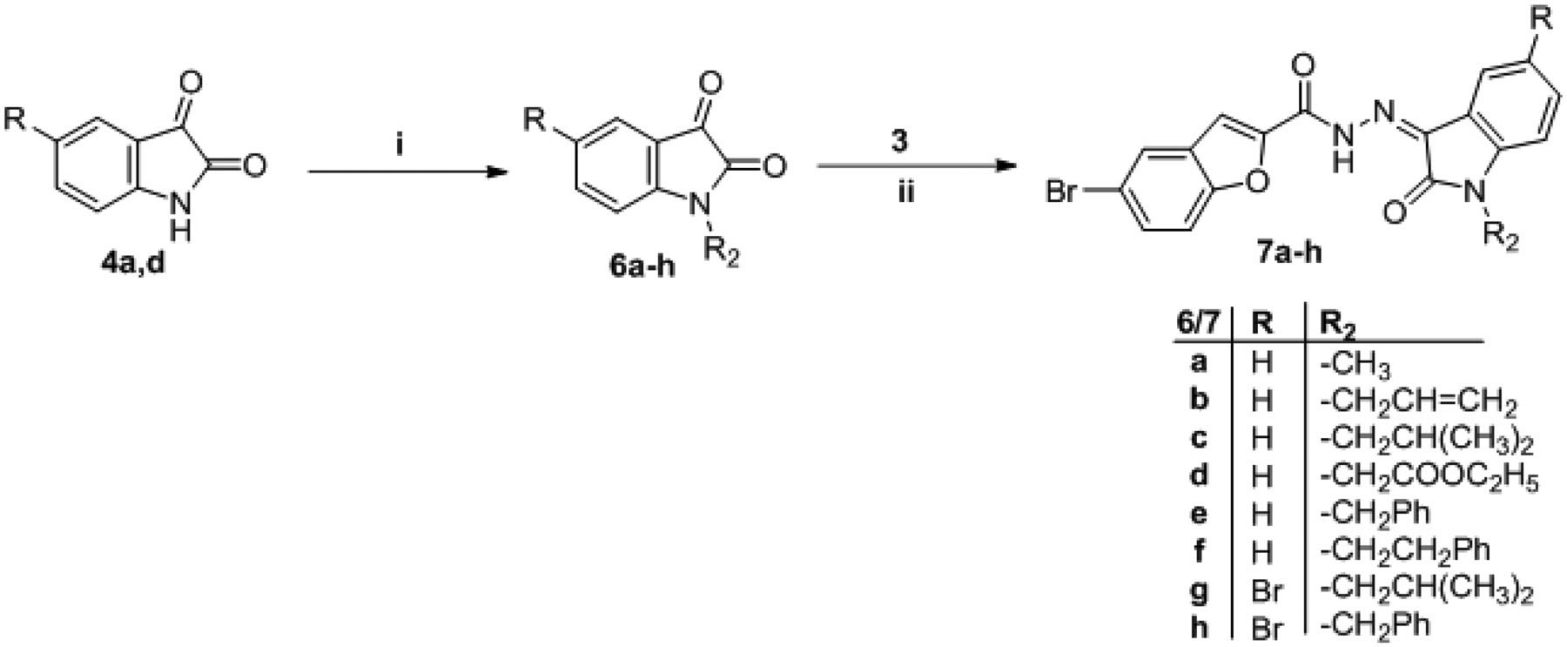
Synthesis of target compounds **7a-h**; Reagents and conditions: (**i**) R_2_-Br/anhydrous DMF/K_2_CO_3_/reflux 4 h, (**ii**) Ethanol/drops glacial acetic acid (Cat.)/reflux 4–7 h.

**Scheme 3.**
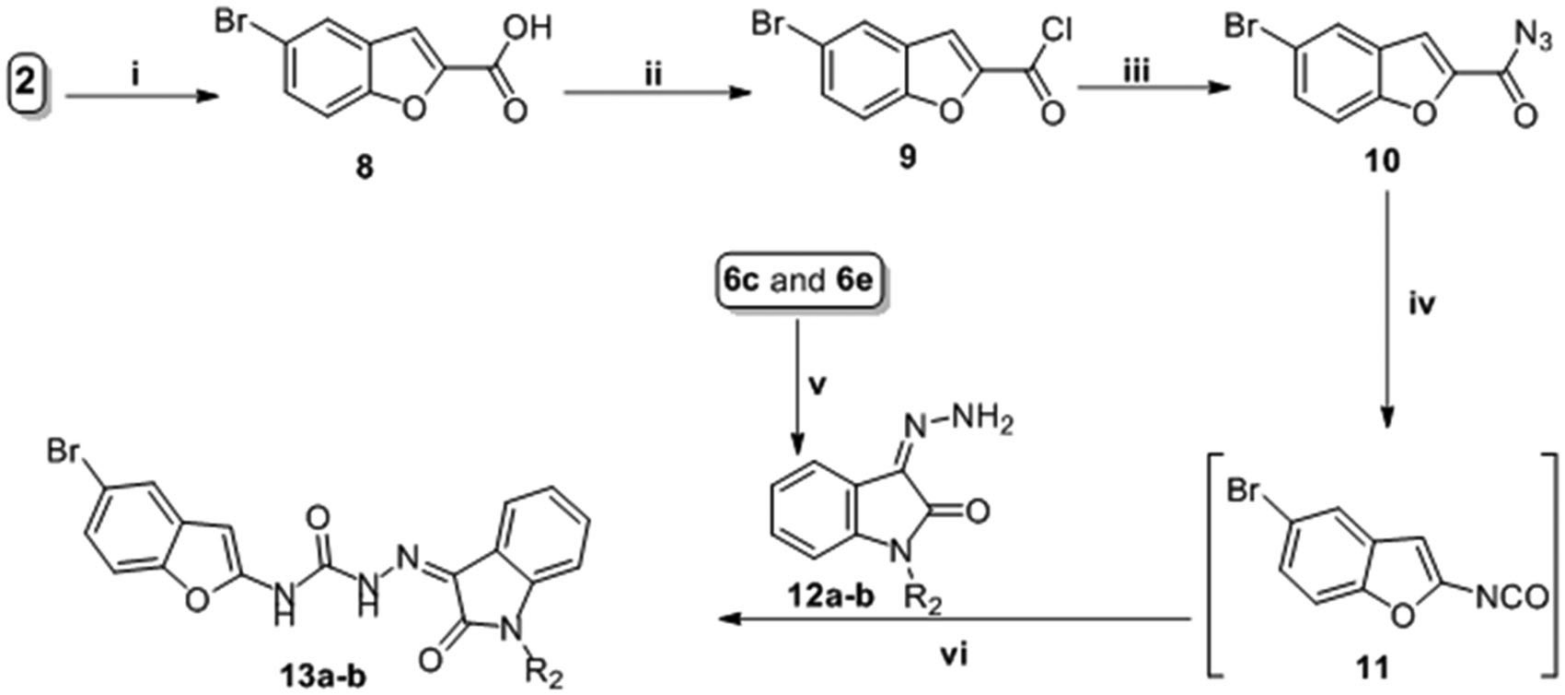
Synthesis of target compounds **13a-b**; Reagents and conditions: (**i**) HCl/reflux 6 h, (**ii**) (COCl)_2_/anhydrous toluene/reflux 5 h, (**iii**) NaN_3_/acetone/stirring r.t. 1 h, (**iv**) Dry toluene/reflux 1 h. (**v**) NH_2_NH_2_.H_2_O/methanol/reflux 3 h, (**vi**) Anhydrous toluene/reflux 4 h.

The synthetic strategies designed for the preparation of the target final compounds (**5a-g**, **7a-h** and **13a-b**) were illustrated in Schemes 1–3. In Scheme 1, bromobenzofuran-2-carbohydrazide **3** was prepared *via* reaction of ethylbromacetate with 5-bromosalicylaldehyde **1** in acetonitrile to furnish ethyl 5-bromobenzofuran-2-carboxylate **2**. Thereafter, the ester functionality was subjected to hydrazinolysis via refluxing hydrazine hydrate to afford the key intermediate hydrazide **3**, further; this key intermediate **3** was condensed with different isatin derivatives **4a-g** in absolute ethyl alcohol with catalytic drops of glacial acetic acid to get the final novel compounds **5a-g**.

In Scheme 2, isatin derivatives **4a** and **4d** were subjected to *N*-alkylation in anhydrous DMF to furnish *N*-substituted isatin **6a-h**, which then condensed with the key intermediate **3** to afford target compounds **7a-h**.

In the last scheme, the ester moiety of 5-bromobenzofuran-2-carboxylate **2** succumbed to acidic hydrolysis to afford the acid analogue **8**, which was chlorinated with oxalyl chloride in toluene to get the acid chloride derivative **9** that reacted with NaN_3_ in acetone to produce 5-bromobenzofuran-2-carbonyl azide **10**. The azide derivative **10** was subjugated to Curtius Rearrangement through heating in anhydrous toluene to furnish isocyanate analogue **11**. On the other hand, isatin derivatives **6c** and **6e** were condensed with hydrazine hydrate to release their hydrazone analogue **12a** and **12 b**, respectively, which reacted with isocyanate analogue **11** to get the final target compounds **13a-b**.

The structures of all the synthesised compounds were confirmed under the basis of spectral and elemental analyses which were in full agreement with the proposed structures.

### 2.2. Biological evaluation

All the newly synthesised compounds were initially tested for their anti-proliferative activity against breast cancer cell lines T-47D and MCF-7 in MTT assay. Compounds showed promising anti-proliferative activity were tested for their PK inhibitory activity on the target kinases CDK-2 and GSK-3β. Furthermore, compounds showed prominent activity at cellular level and on enzymes were further evaluated for their effect on cell cycle progression and cell apoptosis in the breast cancer cell line MCF-7.

#### 2.2.1. In vitro *anti-proliferative activity*

All the newly synthesised benzofuran-oxindole hybrids were screened for their *in vitro* anti-proliferative activity against the breast cancer cell lines T-47D and MCF-7 using the MTT assay, and the results were compared with the pan-kinase inhibitor staurosporine as a reference standard ^102^. The IC_50_ values are presented in Table 1.

**Table 1.** *In vitro* anti-proliferative activity of hybrids **5a–g**, **7a–h** and **13a–b** against breast T-47D and MCF-7 cancer cell lines.
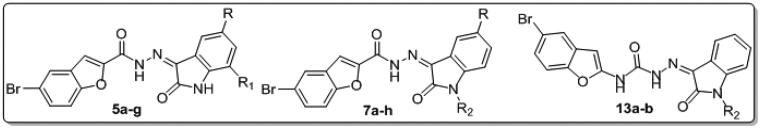

Comp.	R	R_1_	R_2_	IC_50_ (µM)^a^	
				MCF-7	T-47D
**5a**	H	H	–	5.47 ± 0.16	9.67 ± 0.31
**5b**	F	H	–	12.93 ± 0.38	1.27 ± 0.04
**5c**	Cl	H	–	12.46 ± 0.37	6.03 ± 0.19
**5d**	Br	H	–	3.41 ± 0.10	3.82 ± 0.12
**5e**	CH_3_	H	–	3.45 ± 0.10	4.53 ± 0.14
**5f**	OCH_3_	H	–	2.27 ± 0.06	7.80 ± 0.25
**5g**	CH_3_	CH_3_	–	11.95 ± 0.35	6.65 ± 0.21
**7a**	H	–	CH_3_	10.43 ± 1.01	11.79 ± 1.05
**7b**	H	–	CH_2_CH = CH_2_	2.64 ± 0.07	12.24 ± 0.40
**7c**	H	–	CH_2_CH(CH_3_)_2_	37.04 ± 1.87	43.27 ± 2.39
**7d**	H	–	CH_2_COOC_2_H_5_	7.48 ± 0.22	18.99 ± 0.62
**7e**	H	–	CH_2_Ph	8.33 ± 0.24	11.80 ± 0.38
**7f**	H	–	CH_2_CH_2_Ph	24.67 ± 0.72	38.7 ± 1.26
**7g**	Br	–	CH_2_CH(CH_3_)_2_	24.82 ± 0.73	9.67 ± 0.31
**7h**	Br	–	CH_2_Ph	4.32 ± 0.12	1.72 ± 0.04
**13a**	–	–	CH_2_CH(CH_3_)_2_	18.21 ± 0.53	3.22 ± 0.10
**13b**	–	–	CH_2_Ph	5.70 ± 0.16	10.88 ± 0.35
**Staurosporine**	–	–	–	4.81 ± 0.14	4.34 ± 0.14

^a^ IC_50_ values are the mean ± SD of three separate experiments.

On MCF-7 cell line, the tested hybrids showed an IC_50_ range of 2.27–37.04 µM, with compounds **5d-f**, **7 b** and **7 h** showing potent cytotoxic effect (3.41, 3.45, 2.27, 2.64 and 4.32 µM, respectively) compared to the reference staurosporine which showed an IC_50_ of 4.81 µM. Compounds **5a**, **7d**, **7e** and **13 b** showed moderate cytotoxic activity with IC_50_ of 5.47, 7.48, 8.33 and 5.70, respectively, whereas, the rest of compounds showed weak cytotoxic activity with IC_50_ higher than 10 µM (Table 1).

On T-47D cell line, the tested compounds showed an IC_50_ range of 1.27–43.27 µM, with compounds **5 b**, **5d-e**,**7h** and **13a** showing potent cytotoxic effect (IC_50_ of 1.27, 3.82, 4.53, 1.72 and 3.22 µM, respectively) compared to the reference staurosporine which showed an IC_50_ of 4.34 µM. Compounds **5a**, **5c**, **5f**, **5 g** and **7 g** showed moderate cytotoxic activity with IC_50_ of 9.67, 6.03, 7.80, 6.65 and 9.67 µM, respectively, whereas, the rest of compounds showed weak cytotoxic activity with IC_50_ higher than 10 µM (Table 1).

These results indicate the superior cytotoxic activity of the *N*-unsubstituted isatin derivatives **5a-g** on both breast cancer cell lines with IC_50_ range of 2.27–12.93 µM and 1.27–9.67 µM on MCF-7 and T-47D cell lines, respectively, which could be due to their better binding to the target kinases. Compounds **5d–f** with Br, CH_3_ and OCH_3_ substituent on the isatin nucleus, respectively, showed the best cytotoxic activity (Table 1).

Series **7** showed moderate to weak cytotoxic activity on either or both cell lines, except for the *N*-benzylisatin hybrid **7 h** which showed a potent cytotoxic activity on both cell lines with IC_50_ of 4.32 and 1.72 µM on MCF-7 and T-47D, respectively. These results indicate that the *N*-substitution at the isatin nucleus greatly affects the cytotoxic activity in the newly synthesised compounds mostly in a negative manner (Table 1).

Worthy of note, replacing the amido group in compound **7c** and **7e** with the ureido moiety to afford **13a** and **13 b** greatly enhanced the cytotoxic activity in both cell lines (37.04 and 8.33 µM *vs* 18.21 and 5.70 µM, respectively, in MCF-7) and (43.27 and 11.80 µM *vs* 3.22 and 10.88 µM, respectively, in T-47D) (Table 1).

#### 2.2.2. CDK2 and GSK-3β inhibitory activities

The most potent hybrids on breast cancer cell lines (**series 5**) were further evaluated biochemically for their kinase inhibitory activity on CDK-2 and GSK-3β using their Kinase Assay Kits, and were compared to the pan-kinase inhibitor staurosporine as a reference standard. The results were presented in Table 2.

**Table 2.** Inhibitory activities of compounds **5a–g** against CDK2 and GSK-3β.

Comp.	IC_50_ (nM)^a^	
	CDK2	GSK-3β
**5a**	140.6 ± 7.7	136.9 ± 7.5
**5b**	52.46 ± 2.9	212.3 ± 12
**5c**	85.36 ± 4.6	102 ± 5.6
**5d**	37.77 ± 2.1	32.09 ± 1.7
**5e**	176.5 ± 9.6	80.75 ± 4.4
**5f**	52.75 ± 2.9	40.13 ± 2.2
**5g**	104.8 ± 5.7	75.54 ± 4.1
**Staurosporine**	38.5 ± 2.1	43.38 ± 2.4

^a^ IC_50_ values are the mean ± SD of three separate experiments.

On CDK2, the tested compounds showed potent sub-micromolar inhibitory activity with IC_50_ ranged from 37.80 to 177 nM in comparison to that of the used reference standard (IC_50_ = 38.50 nM). The bromo isatin derivative **5d** showed the most potent inhibitory activity with IC_50_ of 37.80 nM comparable to that of staurosporine. Compounds **5 b**, **5c** and **5f** showed two-digit nanomolar inhibitory activity with IC_50_ of 52.50, 85.40 and 52.80, respectively (Table 2).

On GSK-3β, the tested compounds showed potent sub-micromolar inhibitory activity as well with IC_50_ range of 32.09–212.30 nM in comparison to the used reference standard (IC_50_ = 43.38 nM). The bromo isatin **5d** and the methoxy isatin **5f** derivative showed more potent inhibitory activity than that of staurosporine (IC_50_ of 32.09 and 40.13 nM, respectively). Compounds **5e** and **5 g** showed two-digit nanomolar inhibitory activity with IC_50_ of 75.54 and 80.75 nM, respectively (Table 2).

These results indicate the superiority of the bromo isatin **5d** and the methoxy isatin **5f** derivatives on both kinases (Table 2). The methyl isatin derivative **5e** shows a relatively less kinase inhibitory activity than its bromo **5d** and methoxy **5f** congeners on both kinases despite of its obvious potent cellular cytotoxicity which could be attributed to its probable further cytotoxic mechanisms besides CDK2/GSK-3β dual inhibition.

#### 2.2.3. Cell cycle analysis

Cyclin E/CDK2 complexation plays an important role at G1 phase, whereas, cyclin A/CDK2 complexation terminates S phase and drives the cell cycle through normal G2/M phase progression^25^^,^^37^^,^^103^^,^^104^. Furthermore, a significant percentage of CDK2-deficient cells arrest in the G2/M phase, additionally, breast cancer cells exposed to CDK2 inhibitors show G2/M phase arrest^23^^,^^105^. In the same vein, GSK-3β regulates the strength of the mitotic checkpoint and connects the PI3K and WNT-signalling pathways to mitosis, furthermore, GSK-3β inhibitors induce G2/M phase arrest as well^30^^,^^106^^,^^107^.

**Figure 5.**
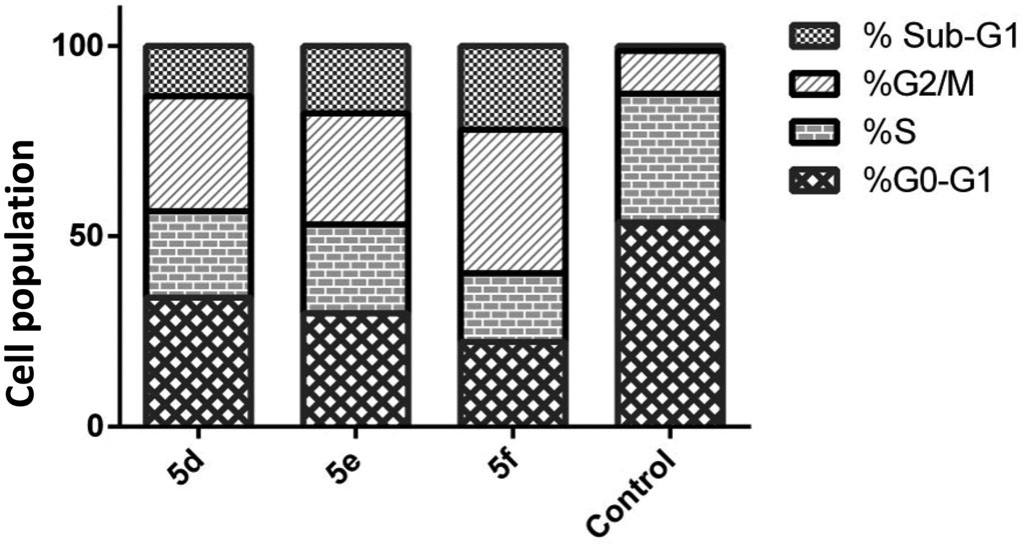
Effect of hybrids **5d**, **5e** and **5f** on the phases of cell cycle of MCF-7 cells.

Compounds showing promising antiproliferative as well as kinase inhibitory activity **5d**, **5e** and **5f** were further investigated for their influence on the cell cycle progression by flow cytometry analysis using propidium iodide (PI) stain. Cell cycle parameters were compared for the breast cancer MCF-7 cells with DMSO as control and after treatment with the compounds of interest and incubation for 24 h, and the results were presented in Figure 5 and Table 3.

**Table 3.** Effect of compounds **5d**, **5e** and **5f** on the phases of cell cycle of MCF-7 cells

Comp.	%G0-G1	%S	%G2/M	%Sub-G1
**5d**	33.97	22.5	30.26	13.27
**5e**	29.85	23.21	29.21	17.73
**5f**	22.28	17.88	37.82	22.02
**Control**	53.59	33.82	11.3	1.29

From the obtained results Figure 5 and Table 3, it is noticeable that there is an increase in the percent of cell distribution in the G2/M phase, from 11.30% in control to 30.26, 29.21 and 37.82% in treated cells with **5d**, **5e** and, **5f**, respectively, indicating a G2/M phase arrest which is the expected consequence from CDK2/GSK-3β dual inhibition confirming the mechanism of action of the designed compounds on the target kinases. Furthermore, an increase in the percent of cells accumulated in the sub-G1 phase, from 1.29% in the control to 13.27, 17.73 and 22.02% in the treated cells with **5d**, **5e** and, **5f**, respectively, as a result of cell apoptosis.

The high efficacy of compound **5f** in cell arrest at G2/M phase and in cell apoptosis as indicated by its results in Table 3 relative to its congeners **5d** and **5e** aligns with its potent effect on MCF-7 as indicated by its IC_50_ (2.27 μM) in comparison to the IC_50_ of compounds **5d** and **5e** (3.41 and 3.45 μM, respectively).

#### 2.2.4. Apoptosis assay

To investigate further the effect of the promising compounds **5d**, **5e**, and **5f** on cell apoptosis, Annexin V-FITC/propidium iodide dual staining assay was performed according to the reported method^108^. The morphological markers of apoptosis in the breast cancer MCF-7 cell line were examined before and after treatment with the compounds of interest. Apoptosis assay depends on the translocation of the phosphatidylserine (PS) phospholipid to the cell surface in cells undergoing apoptosis which can be easily detected by staining with the fluorescent conjugate of annexin V followed by flow cytometry analysis. Simultaneously, MCF-7 cells were stained with propidium iodide (PI) which could enter cells with damaged plasma membranes only. This enables the discrimination between early apoptotic cells (positive for PS, but negative for PI) from late apoptotic and necrotic cells (positive for both PS and PI).

**Figure 6.**
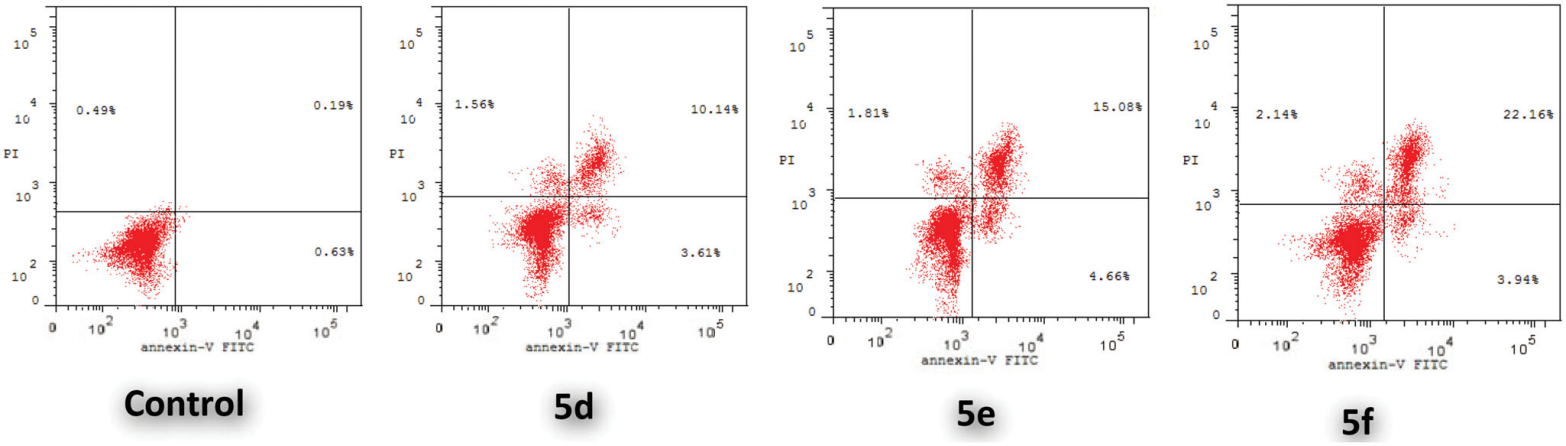
Effect of **5d**, **5e** and **5f** on the percentage of annexin V-FITC-positive staining in MCF-7 cells. The experiments were done in triplicates. The four quadrants identified as: **LL**, viable; **LR**, early apoptotic; **UR**, late apoptotic; **UL**, necrotic.

Figure 6 and Table 4 show that the percentage of the total apoptotic cells in MCF-7 cell line increases after treatment with compounds **5d**, **5e**, and **5f** (13.75, 19.74, and 26.10%, respectively) relative to control cells (0.82%) which is a significant indication of the apoptotic effect of the compounds of interest. Compounds **5d**, **5e**, and **5f** produced an increase in the early apoptotic phase, from 0.63 to 3.61, 4.66, and 3.94, respectively, and an increase in the late apoptotic phase, from 0.19 to 10.14, 15.08, and 22.16, respectively.

**Table 4.** Distribution of apoptotic cells in the AnnexinV-FITC/PI dual staining assay in MCF-7 cells upon treatment with compounds **5d**, **5e** and **5f**.

Comp.	Total (L.R % + U.R %)	Early Apoptosis (Lower Right %)	Late Apoptosis (Upper Right %)	Necrosis
**5d**	13.75	3.61	10.14	1.56
**5e**	19.74	4.66	15.08	1.81
**5f**	26.10	3.94	22.16	2.14
**Control**	0.82	0.63	0.19	0.49

#### 2.2.5. Cytotoxicity towards non-tumorigenic cells

In order to investigate their selectivity on cancer cells, hybrids **5d**, **5e** and **5f**, endowed with dual growth inhibitory action against MCF-7 and T-47D cells, were assesses for their cytotoxic action towards the non-tumorigenic breast cells (MCF-10A), *via* the MTT assay. The obtained IC_50_s and the calculated mean tumour selectivity index (S.I.); IC_50_ for MCF-10A/IC_50_ average for (MCF-7 and T-47D) have been presented in Table 5. The examined hybrids exerted weak cytotoxic effect (IC_50_ = 21.66 ± 1.05, 23.59 ± 0.86 and 39.95 ± 1.42 μM, respectively, with selectivity indexes equal 6.0, 5.9 and 7.9, respectively, Table 5.

**Table 5.** Cytotoxic action for hybrids **5d**, **5e** and **5f** towards non-tumorigenic breast cell line (MCF-10A), and mean tumour selectivity index (S.I.) (MCF-10A/MCF-7 and T-47D).

Comp.	IC_50_ (µM)			Mean tumour selectivity
	MCF-10A	MCF-7	T-47D	
**5d**	21.66 ± 1.05	3.41 ± 0.10	3.82 ± 0.12	6.0
**5e**	23.59 ± 0.86	3.45 ± 0.10	4.53 ± 0.14	5.9
**5f**	39.95 ± 1.42	2.27 ± 0.06	7.80 ± 0.25	7.9

## 3. Molecular docking study and structure activity relationship

In the present molecular docking study, a pair of protein structures were used for CDK2 and GSK-3β, *viz*, PDB ID: 1FVT^109^ and PDB ID: 1Q41^110^, respectively, which are co-crystallized with potent CDK2 and GSK-3β oxindole-based inhibitors, respectively. Molecular docking was carried out to investigate the interaction of the designed hybrid compounds with CDK2 and GSK-3β kinase domain to rationalise their biological activity, to reveal their probable binding pattern and to elicit their SAR.

**Figure 7.**
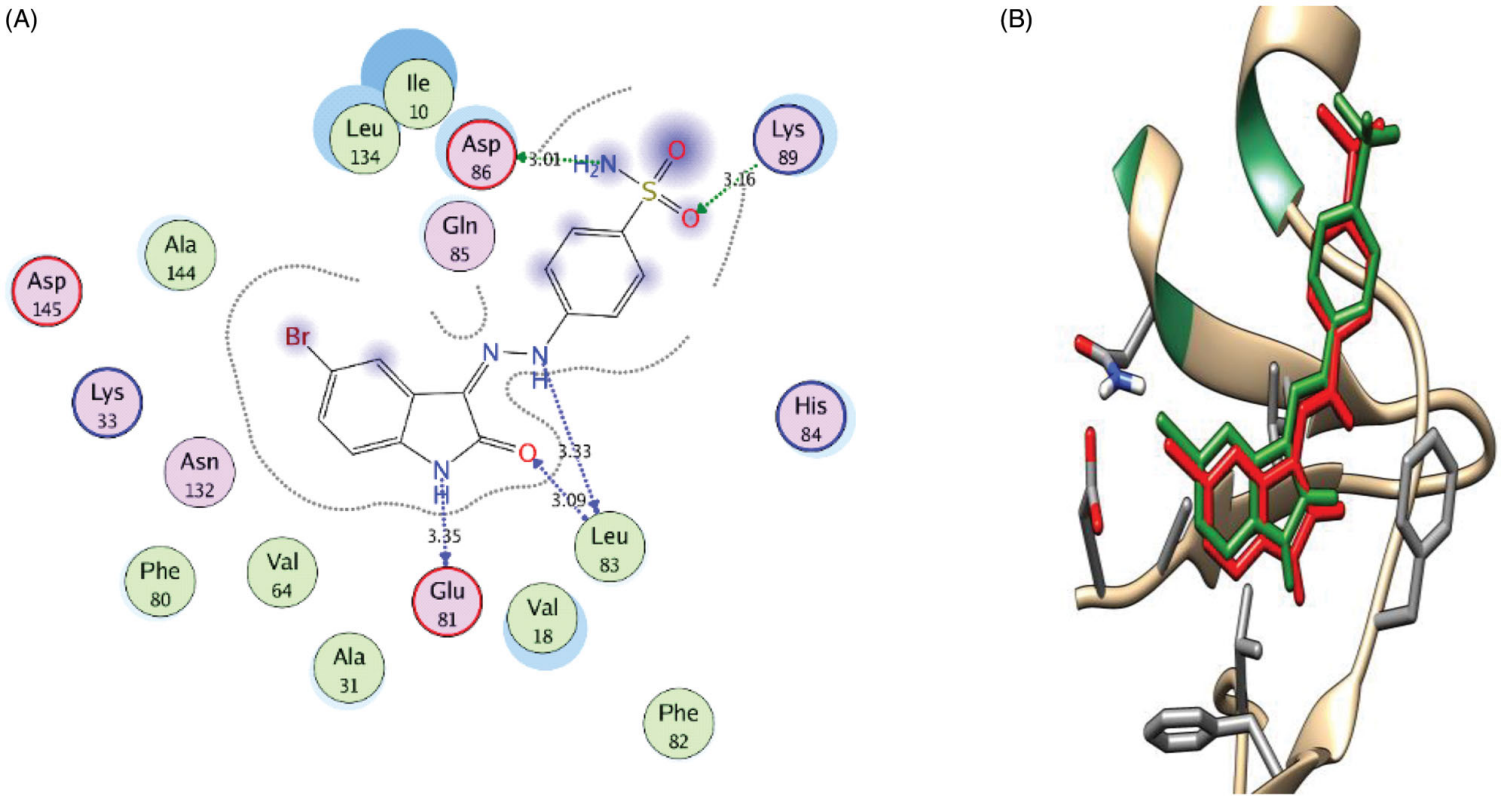
(**A**) 2 D interaction diagram showing the oxindole derivative docking pose interactions with the key amino acids (hot spots) in the CDK2 active site. (Distances in Å) (**B**) 3 D representations of the superimposition of the docking pose (green) and the co-crystallized pose (red) of the oxindole derivative in the CDK2 active site, respectively, with RMSD of 0.894 Å.

**Figure 8.**
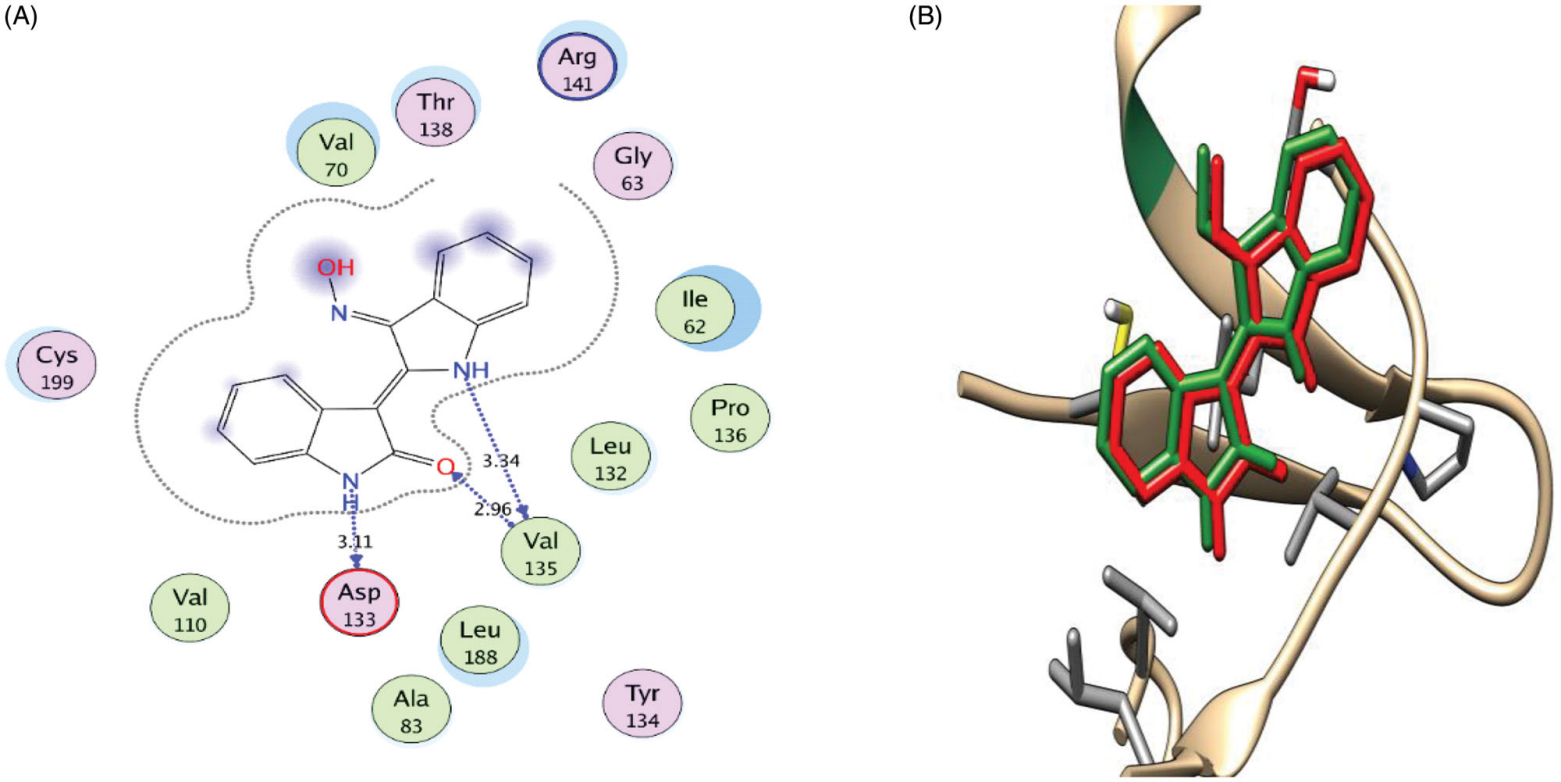
(**A**) 2 D interaction diagram showing Indirubin-3'-monoxime docking pose interactions with the key amino acids (hot spots) in the GSK-3β active site. (Distances in Å) (**B**) 3 D representations of the superimposition of the docking pose (green) and the co-crystallized pose (red) of Indirubin-3'-monoxime in the GSK-3β active site, respectively, with RMSD of 0.471 Å.

Initially, self-docking of the co-crystallized ligands in CDK2 and GSK-3β active sites was performed to validate the used molecular docking protocol. The self-docking step stimulated the binding pattern of the co-crystallized ligands accurately indicating the suitability of the used docking setup for the planned simulations. This was demonstrated by the small RMSD between the docked and the co-crystallized ligand poses in CDK2 (0.894 Å) and GSK-3β (0.471 Å), and by the ability of the attained docking poses to simulate the main interactions achieved by the co-crystallized ligands with the key amino acids in CDK2 and GSK-3β active sites (Figures 7 and 8, respectively).

In the CDK2 active site the docking pose of the oxindole derivative reproduced the key interactions of the co-crystalized ligand with the active site; it interacts in the hinge region through hydrogen bonding with Glu81 backbone CO and Leu83 backbone NH and CO. Furthermore, its sulfonamidophenylhydrazone group projected outward towards the bulk solvent with the sulphonamide group interacting with Asp86 backbone NH and side-chain carboxyl group and Lys89 side chain NH_3_^+^ (Figure 7).

As for GSK-3β, the docking pose of indirubin-3′-monoxime reproduced the key interactions of the co-crystalized ligand with the active site; it interacts in the hinge region through hydrogen bonding with Asp133 backbone CO and Val135 backbone NH and CO (Figure 8). Furthermore, through a water mediated hydrogen bonding network, it interacts with Gln185 and Thr138 by its oxime group which is responsible for its selectivity towards GSK-3β over CDK2.

**Figure 9.**
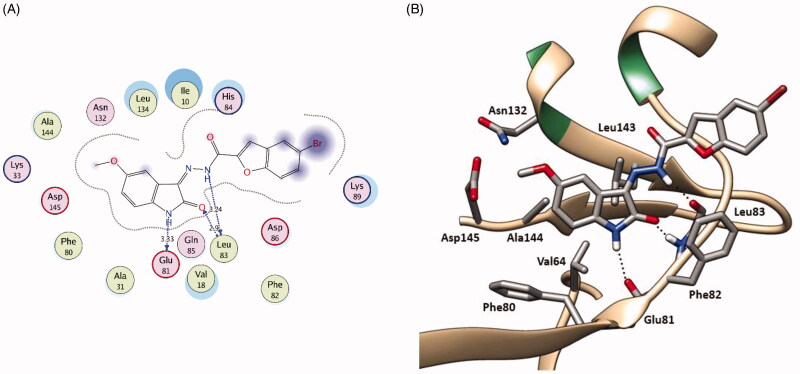
2 D diagram (**A**) and 3 D representation (**B**) of compound **5f** showing its interaction with the CDK2 active site.

**Figure 10.**
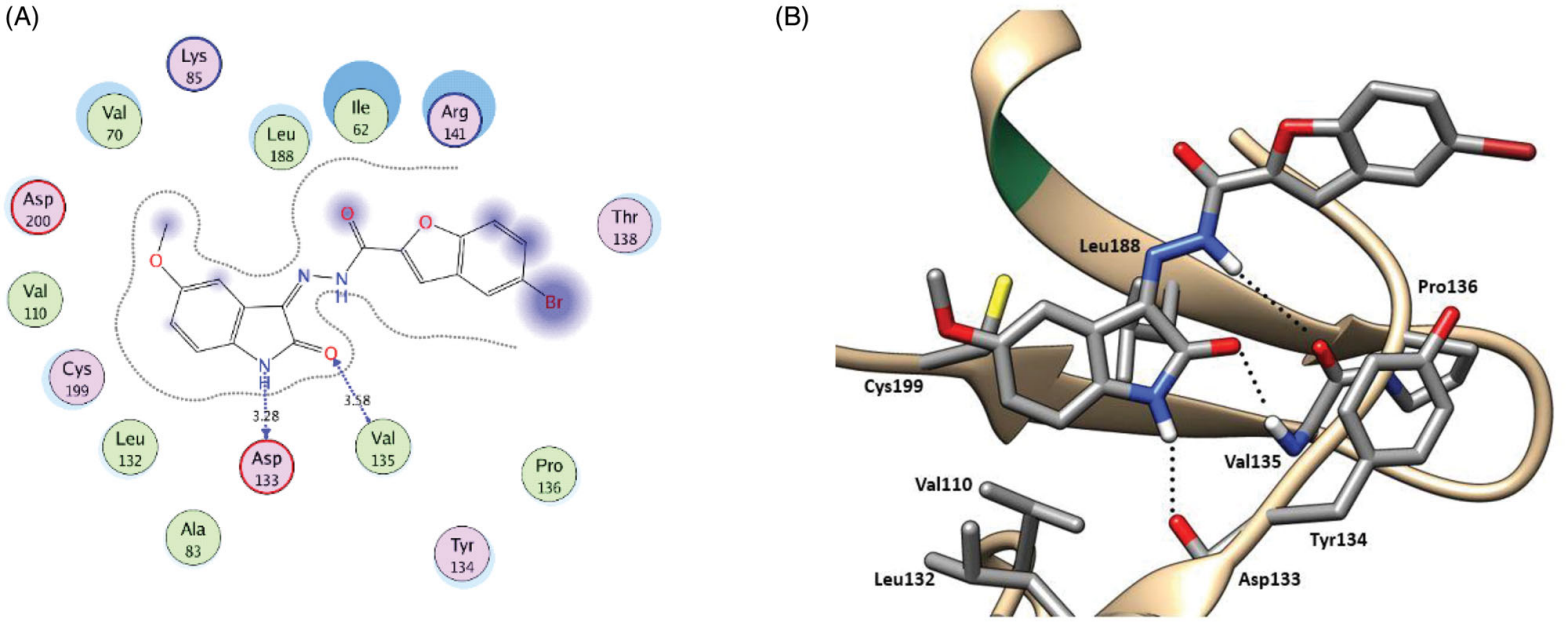
2 D diagram (**A**) and 3 D representation (**B**) of compound **5f** showing its interaction with the GSK-3β active site.

In series **5**, the newly synthesised *N^1^*-unsubstituted oxindole hybrids showed comparable binding patterns in both kinases (Figures 9 and 10 and for further details, see supporting materials). The oxindole ring is accommodated in the hinge region interacting through hydrogen bonding by its NH and CO with the backbone CO and NH of the key amino acids Glu81 and Leu83, respectively, in CDK2, and Asp133 and Val135, respectively, in GSK-3β.

Moreover, through hydrophobic interaction the fused phenyl ring of the oxindole nucleus interacts with the hydrophobic side chains of the surrounding amino acids; Val18, Ala31, Val64, Phe80, Leu134, and Ala144 in CDK2 and Val70, Ala83, Val110, Leu132, Leu188, and Cys199 in GSK-3β (Figures 9 and 10 and for further details, see supporting materials). Therefore, increasing the substituent hydrophobicity on the oxindole nucleus enhances the binding affinity which is well reflected in the docking binding score and further in the biological activity (Br, **5d** > Cl, **5c** ≈ CH_3_, **5e** > F, **5 b**) (Tables 1, 2 and 6). The remarkable binding affinity of the relatively polar methoxy group (OCH_3_, **5f**) is attributed to the fact that it is directed towards the bulk solvent and so decreased the solvation penalty during the binding scenario resulting in a considerable increase in the binding affinity. On the other hand, despite of its relatively higher hydrophobicity, the dimethyl substituted hybrid **5 g** does not show significant predicted binding score or experimental biological activity what could be attributed to the C^7^ methyl substituent which sterically clashes with the bottom of the hinge region hindering the key interaction of the NH and CO of the oxindole ring with the key amino acids; Glu81 and Leu83 in CDK2, respectively, and Asp133 and Val135 in GSK-3β, respectively (For further details, see supporting materials).

**Table 6.** Docking energy scores (*S*) in kcal/mol for the newly synthesised hybrid compounds and the co-crystalized compounds in CDK2 and GSK-3β kinase domain

Compound	Energy score (S) kcal/mol CDK2	Energy score (S) kcal/mol GSK-3β
5a	−9.53	−9.99
5b	−9.89	−10.40
5c	−10.07	−10.59
5d	−10.22	−10.81
5e	−10.05	−10.53
5f	−10.35	−10.96
5g	−10.39	−10.60
7a	−9.19	−10.40
7b	−9.61	−10.24
7c	−10.03	−11.48
7d	−10.21	−11.76
7e	−9.98	−11.58
7f	−10.05	−10.68
7g	−10.56	−11.27
7h	−10.40	−11.46
13a	−13.60	−11.77
13b	−14.72	−11.92
Co-crystalized ligand	−10.63	−12.00

The hydrazono linker NH interacts through hydrogen bonding with the backbone CO of Leu83 and Val135 in CDK2 and GSK-3β, respectively, projecting the benzofuran moiety outward towards the bulk solvent which is involved in a hydrophobic interaction with the hydrophobic side chains of the surrounding amino acids lining the gate of the hinge region; Ile10, Phe82, and Leu298 in CDK2 and Ile62, Tyr134 and Pro136 in GSK-3β (Figures 9 and 10 and for further details, see supporting materials).

In series **7** and **13**, the *N^1^*-substitutions on the oxindole nucleus hinder the compounds from achieving the key interactions with hinge region amino acids; Glu81 and Leu83 in CDK2, and Asp133 and Val135 in GSK-3β what rationalises their moderate to low antiproliferative activity (For further details, see supporting materials). Molecular docking simulations show their non-selective bindings and random binding patterns what rationalise their low activity despite of their good docking binding scores (Table 6).

These results point out the criticality of N^1^ being unsubstituted for CDK2/GSK-3β kinase inhibition. The N^1^-benzyloxindole derivative **7 h** showing promising antiproliferative activity might be exerting its effect through other mechanism than CDK2/GSK-3β kinase inhibition.

## 4. Conclusions

In the present study three series of oxindole-benzofuran hybrids were designed and synthesised as dual CDK2/GSK-3β inhibitors targeting breast cancer (**5a-g**, **7a-h**, and **13a-b**). In MTT assay on the breast cancer cell lines MCF-7 and T-47D, the *N^1^*-unsubstituted oxindole derivatives, **series 5**, showed moderate to potent activity on both breast cancer cell lines. Compounds **5d-f** showed the most potent cytotoxic activity with IC_50_ of 3.41, 3.45 and 2.27 μM, respectively, on MCF-7 and IC_50_ of 3.82, 4.53 and 7.80 μM, respectively, on T-47D cell lines, in comparison to the used reference standard (staurosporine) IC_50_ of 4.81 and 4.34 μM, respectively. On the other hand, the *N^1^*-substituted oxindole derivatives, **series 7 and 13**, showed moderate to weak cytotoxic activity on both breast cancer cell lines except for compound **7 h** which showed potent antiproliferative activity with IC_50_ of 4.32 and 1.72 μM on MCF-7 and T-47D cell lines, respectively. CDK2 and GSK-3β inhibition testing of the potent **series 5** indicated that compounds **5d** and **5f** exhibit potent dual CDK2/GSK-3β inhibitory activity with IC_50_ of 37.77 and 52.75 nM, respectively, on CDK2 and 32.09 and 40.13 nM, respectively, on GSK-3β. The most potent hybrids **5d-f** triggered cell cycle arrest in the G2/M phase as a consequence of their dual CDK2/GSK-3β inhibition. Moreover, compounds **5d-f** induced apoptosis in MCF-7 cells as indicated by the percent of the total apoptosis of 13.75, 19.74, and 26.10%, respectively, in comparison to the untreated cells which showed 0.82% total apoptosis. The molecular docking study showed that the newly synthesised *N^1^*-unsubstituted oxindole hybrids have comparable binding patterns in both CDK2 and GSK-3β. The oxindole ring is accommodated in the hinge region interacting through hydrogen bonding with the backbone CO and NH of the key amino acids Glu81 and Leu83, respectively, in CDK2 and Asp133 and Val135, respectively, in GSK-3β. Moreover, through hydrophobic interaction the fused phenyl ring of the oxindole nucleus interacts with the hydrophobic side chains of the surrounding amino acids; Val18, Ala31, Val64, Phe80, Leu134, and Ala144 in CDK2 and Val70, Ala83, Val110, Leu132, Leu188, and Cys199 in GSK-3β. The hydrazono linker NH interacts through hydrogen bonding with the backbone CO of Leu83 and Val135 in CDK2 and GSK-3β, respectively, projecting the benzofuran moiety outward towards the bulk solvent which is involved in a hydrophobic interaction with the hydrophobic side chains of the surrounding amino acids lining the gate of the hinge region; Ile10, Phe82, and Leu298 in CDK2 and Ile62, Tyr134 and Pro136 in GSK-3β. In series **7** and **13**, the *N^1^*-substitutions on the oxindole nucleus hinder the compounds from achieving the key interactions with hinge region amino acids; Glu81 and Leu83 in CDK2, and Asp133 and Val135 in GSK-3β what rationalises their moderate to low antiproliferative activity.

## 5. Experimental

### 5.1. Chemistry

#### 5.1.1. General

Melting points were measured with a Stuart melting point apparatus and were uncorrected. Infra-red spectra were recorded on Schimadzu FT-IR 8400S spectrophotometer. The NMR spectra were recorded by Bruker spectrometer at 400 MHz. ^13 ^C NMR spectra were run at 100 MHz in deuterated dimethylsulphoxide (DMSO*d6*). Elemental analyses were carried out at the Regional Centre for Microbiology and Biotechnology, Al-Azhar University, Cairo, Egypt. Unless otherwise noted, all solvents and reagents were commercially available and used without further purification. Compounds (**2** and **3**)^97^, (**6a–h**)^111–112^, (**8–9**)^113^, and (**12 b**)^114^ were prepared according to the reported methods.

##### 5.1.1.2. Synthesis of 5-bromobenzofuran-2-carbohydrazide 3

To hot stirred solution of ethyl 5-bromobenzofuran-2-carboxylate **2** (1 g, 3.7 mmol) in 30 ml of methanol, hydrazine hydrate (0.25 ml, 7.5 mmol) was added. The reaction solution was left for heating under reflux for 4 h, and then poured onto cold water. The formed solid was collected by filtration, washed with diethyl ether and recrystallized from EtOH/DMF mixture to furnish the key intermediate **3**.

##### 5.1.1.3. Synthesis of target compounds 5a–g and 7a–h

5-Bromobenzofuran-2-carbohydrazide **3** (0.3 g, 1.2 mmol) was added to a hot solution of equivalent amount of the appropriate isatin derivative (**4a–g** or **6a–h**) in ethanol (15 ml) with catalytic amount of ethanoic acid. The reaction mixture was heated under reflux for 4–7 h with TLC monitoring, once the reaction completed, the reaction mixture was left for cooling then was filtered-off. The produced solid was washed with water, diethyl ether and recrystallized from dioxane/propanol mixture to produce target compounds **5a–g** and **7a–h**, respectively.

###### 5.1.1.3.1. 5-Bromo-N'-(2-oxoindolin-3-ylidene)benzofuran-2-carbohydrazide 5a

Yellow powder (yield 75%), m.p. > 300 °C; ^1^H NMR *ppm*: 6.94–7.00 (m, 1H, Ar-H), 7.11 (t, 1H, Ar-H, *J* = 8.0 Hz), 7.41–7.47 (m, 1H, Ar-H), 7.64–7.84 (m, 3H, Ar-H), 7.99 (t, 1H, Ar-H, *J* = 8.0 Hz), 8.09, 8.11 (2 s, 1H, Ar-H), 10.91, 11.39 (2 s, 1H, NH indolin-2-one), 11.89, 14.06 (2 s, 1H, NH); ^13 ^C NMR *δ ppm*: 111.32, 114.73, 116.72, 120.10, 121.76, 122.45, 123.30, 126.13, 127.23, 129.61, 131.18, 132.70, 133.70, 143.29, 153.87, 163.35 (C = O indolin-2-one), 165.04 (C = O hydrazide); IR (KBr, *ν* cm^−1^) 3380, 3345 (2NH) and 1711, 1701 (2 C = O); MS *m/z* [%]: 384 [M^+^+2, 50.94], 382 [M^+^, 48.60], 160 [100]; Analysis calculated for C_17_H_10_BrN_3_O_3_: C, 53.15; H, 2.62; N, 10.94; found C, 53.33; H, 2.64; N, 10.82.

###### 5.1.1.3.2. 5-Bromo-N'-(5-fluoro-2-oxoindolin-3-ylidene)benzofuran-2-carbohydrazide 5b

Red powder (yield 77%), m.p. > 300 °C; ^1^H NMR *ppm*: 6.92–7.00 (m, 1H, Ar-H), 7.24–7.33 (m, 1H, Ar-H), 7.49, 7.86 (2br s, 1H, Ar-H), 7.67–7.71 (m, 1H, Ar-H), 7.74 (t, 1H, Ar-H, *J* = 8.0 Hz), 7.96 (d, 0.6H, Ar-H, *J* = 8.0 Hz), 8.04–8.13 (m, 1.4H, Ar-H), 10.92,11.42 (2 s, 1H, NH indolin-2-one), 12.02, 14.02 (2 s, 1H, NH); IR (KBr, *ν* cm^−1^) 3280, 3245 (2NH) and 1723, 1705 (2 C = O); Analysis calculated for C_17_H_9_BrFN_3_O_3_: C, 50.77; H, 2.26; N, 10.45; found C, 50.90; H, 2.24; N, 10.57.

###### 5.1.1.3.3. 5-Bromo-N'-(5-chloro-2-oxoindolin-3-ylidene)benzofuran-2-carbohydrazide 5c

Orange powder (yield 83%), m.p. > 300 °C; ^1^H NMR *ppm*: 6.94–7.01 (m, 1H, Ar-H), 7.45–7.50 (m, 1H, Ar-H), 7.64–7.76 (m, 2.3H, Ar-H), 8.04, 8.10 (2 s, 1H, Ar-H), 8.14–8.17 (m, 1.7H, Ar-H), 11.03,11.50 (2 s, 1H, NH indolin-2-one), 12.08, 13.96 (2 s, 1H, NH); ^13 ^C NMR *δ ppm*: 112.66, 114.60, 116.94, 118.92, 121.28, 124.34, 126.29, 126.77, 131.04, 132.93, 137.31, 141.43, 143.41, 153.82, 163.17 (C = O indolin-2-one), 164.88 (C = O hydrazide); IR (KBr, *ν* cm^−1^) 3300, 3240 (2NH) and 1735, 1702 (2 C = O); Analysis calculated for C_17_H_9_BrClN_3_O_3_: C, 48.77; H, 2.17; N, 10.04; found C, 48.82; H, 2.16; N, 9.93.

###### 5.1.1.3.4. 5-Bromo-N'-(5-bromo-2-oxoindolin-3-ylidene)benzofuran-2-carbohydrazide 5d

Orange powder (yield 87%), m.p. > 300 °C; ^1^H NMR *ppm*: 6.91–6.97 (m, 1H, Ar-H), 7.60 (d, 1H, Ar-H, *J* = 8.0 Hz), 7.71–7.76 (m, 2H, Ar-H), 8.03–8.15 (m, 2H, Ar-H), 8.31 (s, 1H, Ar-H), 11.05,11.52 (2 s, 1H, NH indolin-2-one), 12.09, 13.99 (2 s, 1H, NH); IR (KBr, *ν* cm^−1^) 3295, 3271 (2NH) and 1732, 1701 (2 C = O); Analysis calculated for C_17_H_9_Br_2_N_3_O_3_: C, 44.09; H, 1.96; N, 9.07; found C, 43.81; H, 1.98; N, 9.16.

###### 5.1.1.3.5. 5-Bromo-N'-(5-methyl-2-oxoindolin-3-ylidene)benzofuran-2-carbohydrazide 5e

Brown powder (yield 84%), m.p. > 300 °C; ^1^H NMR *ppm*: 2.23, 2.35 (2 s, 3H, CH_3_), 6.84–6.88 (m, 1H, Ar-H), 7.22–7.27 (m, 1H, Ar-H), 7.47 (s, 0.6H, Ar-H), 7.68–7.71 (m, 1H, Ar-H), 7.74–7.79 (m, 1H, Ar-H), 7.82–7.88 (m, 1H, Ar-H), 8.00 (s, 0.4H, Ar-H), 8.09–8.14 (m, 1H, Ar-H), 10.80, 11.28 (2 s, 1H, NH indolin-2-one), 11.83, 14.05 (2 s, 1H, NH) ; ^13 ^C NMR *δ ppm*: 20.97 (CH_3_), 111.57, 114.65, 116.89, 120.09, 122.08, 126.14, 127.54, 129.60, 130.92, 131.15, 132.44, 133.12, 141.02, 142.44, 153.84, 163.41 (C = O indolin-2-one), 165.09 (C = O hydrazide); IR (KBr, *ν* cm^−1^) 3320, 3341 (2NH) and 1721, 1700 (2 C = O); Analysis calculated for C_18_H_12_BrN_3_O_3_: C, 54.29; H, 3.04; N, 10.55; found C, 54.57; H, 3.01; N, 10.62.

###### 5.1.1.3.6. 5-Bromo-N'-(5-methoxy-2-oxoindolin-3-ylidene)benzofuran-2-carbohydrazide 5f

Red powder (yield 82%), m.p. > 300 °C; ^1^H NMR *ppm*: 3.80, 3.83 (2 s, 3H, OCH_3_), 6.85–6.91 (m, 1H, Ar-H), 6.98–7.07 (m, 1H, Ar-H), 7.18 (s, 1H, Ar-H), 7.68–7.76 (m, 2H, Ar-H), 7.82, 8.01 (2 s, 1H, Ar-H), 8.08–8.13 (m, 1H, Ar-H), 10.72,11.20 (2 s, 1H, NH indolin-2-one), 11.97, 14.10 (2 s, 1H, NH); IR (KBr, *ν* cm^−1^) 3324, 3301 (2NH) and 1718, 1710 (2 C = O); MS *m/z* [%]: 416 [M^+^+2, 75.42], 414 [M^+^, 77.51], 402 [100]; Analysis calculated for C_18_H_12_BrN_3_O_4_: C, 52.19; H, 2.92; N, 10.14; found C, 52.01; H, 2.95; N, 10.25.

###### 5.1.1.3.7. 5-Bromo-N'-(5,7-dimethyl-2-oxoindolin-3-ylidene)benzofuran-2-carbohydrazide 5 g

Red powder (yield 76%), m.p. > 300 °C; ^1^H NMR *ppm*: 2.20, 20.22 (2 s, 3H, CH_3_ of C-7 of indolin-2-one), 2.29, 2.32 (2 s, 3H, CH_3_ of C-5 of indolin-2-one), 7.06, 7.11 (2 s, 1H, Ar-H), 7.30 (s, 0.5H, Ar-H), 7.69–7.71 (m, 1.5H, Ar-H), 7.75–7.79 (m, 1H, Ar-H), 7.82, 8.00 (2 s, 1H, Ar-H), 8.09, 8.11 (2 s, 1H, Ar-H), 10.82,11.29 (2 s, 1H, NH indolin-2-one), 11.78, 14.06 (2 s, 1H, NH); ^13 ^C NMR *δ ppm*: 16.31 (CH_3_ C-7 of indolin-2-one), 20.88 (CH_3_ C-5 of indolin-2-one), 114.67, 116.88, 119.47, 120.97, 123.31, 126.13, 129.61, 131.11, 132.34, 134.53, 135.97, 139.65, 141.47, 142.47, 153.84, 163.82 (C = O indolin-2-one), 165.16 (C = O hydrazide); IR (KBr, *ν* cm^−1^) 3350, 3315 (2NH) and 1723, 1701 (2 C = O); MS *m/z* [%]: 414 [M^+^+2, 17.45], 412 [M^+^, 14.19], 160 [100]; Analysis calculated for C_19_H_14_BrN_3_O_3_: C, 55.36; H, 3.42; N, 10.19; found C, 55.53; H, 3.38; N, 10.27.

###### 5.1.1.3.8. 5-Bromo-N'-(1-methyl-2-oxoindolin-3-ylidene)benzofuran-2-carbohydrazide 7a

Yellow powder (yield 81%), m.p. 260–262 °C; ^1^H NMR *ppm*: 3.27 (s, 3H, *N*-CH_3_), 7.19 (t, 2H, Ar-H, *J* = 8.0 Hz), 7.50 (t, 1H, Ar-H, *J* = 8.0 Hz), 7.68–7.71 (m, 2H, Ar-H), 7.76 (d, 1H, Ar-H, *J* = 8.0 Hz), 7.85 (s, 1H, Ar-H), 8.09 (s, 1H, Ar-H), 11.97, 14.02 (2 s, 1H, NH); ^13 ^C NMR *δ ppm*: 26.26 (*N*-CH_3_), 110.59, 114.68, 116.94, 119.40, 121.41, 123.84, 126.16, 129.60, 130.33, 131.24, 132.62, 138.18, 144.51, 151.44, 153.87, 161.58 (C = O indolin-2-one), 165.10 (C = O hydrazide); IR (KBr, *ν* cm^−1^) 3345 (NH) and 1720, 1695 (2 C = O); MS *m/z* [%]: 400 [M^+^+2, 92.41], 398 [M^+^, 94.85], 91 [100]; Analysis calculated for C_18_H_12_BrN_3_O_3_: C, 54.29; H, 3.04; N, 10.55; found C, 54.48; H, 3.02; N, 10.64.

###### 5.1.1.3.9. N'-(1-Allyl-2-oxoindolin-3-ylidene)-5-bromobenzofuran-2-carbohydrazide 7 b

Orange powder (yield 73%), m.p. 207–209 °C; ^1^H NMR *ppm*: 4.46 (s, 2H, *N*-CH_2_), 5.22–5.31 (m, 2H, =CH_2_), 5.88–5.97 (m, 1H, *N*-CH_2_-CH), 7.13 (d, 1H, Ar-H, *J* = 8.0 Hz), 7.19 (t, 1H, Ar-H, *J* = 8.0 Hz), 7.47 (t, 1H, Ar-H, *J* = 8.0 Hz), 7.68–7.72 (m, 2H, Ar-H), 7.76 (d, 1H, Ar-H, *J* = 8.0 Hz),) , 7.85 (s, 1H, Ar-H), 8.09 (s, 1H, Ar-H), 11.99, 13.97 (2 s, 1H, NH); IR (KBr, *ν* cm^−1^) 3304 (NH) and 1715, 1701 (2 C = O); Analysis calculated for C_20_H_14_BrN_3_O_3_: C, 56.62; H, 3.33; N, 9.90; found C, 56.77; H, 3.31; N, 10.01.

###### 5.1.1.3.10. 5-Bromo-N'-(1-isobutyl-2-oxoindolin-3-ylidene)benzofuran-2-carbohydrazide 7c

Yellow powder (yield 65%), m.p. 216–218 °C; ^1^H NMR *ppm*: 0.94 (d, 6H, -CH-(CH_3_)_2_, *J* = 8.0 Hz), 2.09–2.16 (m, 1H, *N*-CH_2_-CH), 3.36 (d, 2H, *N*-CH_2_, *J* = 8.0 Hz), 7.17 (t, 1H, Ar-H, *J* = 8.0 Hz), 7.24 (d, 1H, Ar-H, *J* = 8.0 Hz), 7.47 (t, 1H, Ar-H, *J* = 8.0 Hz), 7.67–7.70 (m, 2H, Ar-H), 7.77 (d, 1H, Ar-H, *J* = 8.0 Hz), 7.84 (s, 1H, Ar-H), 8.08 (s, 1H, Ar-H), 13.99 (s, 1H, NH); IR (KBr, *ν* cm^−1^) 3312 (NH) and 1720, 1711 (2 C = O); Analysis calculated for C_21_H_18_BrN_3_O_3_: C, 57.29; H, 4.12; N, 9.54; found C, 57.08; H, 4.17; N, 9.47.

###### 5.1.1.3.11. Ethyl-2–(3-(2–(5-bromobenzofuran-2-carbonyl)hydrazono)-2-oxoindolin-1-yl)acetate 7d

Orange powder (yield 69%), m.p. 215–216 °C; ^1^H NMR *ppm*: 1.23 (t, 3H, CH_3_, *J* = 8.0 Hz), 4.18 (q, 2H, CH_2_, *J* = 8.0 Hz), 4.76 (s, 2H, *N*-CH_2_), 7.22 (t, 1H, Ar-H, *J* = 8.0 Hz), 7.50 (t, 1H, Ar-H, *J* = 8.0 Hz), 7.96–7.78 (m, 2H, Ar-H), 7.87 (s, 1H, Ar-H), 8.10 (s, 1H, Ar-H), 13.81 (s, 1H, NH); ^13 ^C NMR *δ ppm*: 14.49 (CH_3_), 41.47 (-CH_2_-CH_3_), 61.97 (*N*-CH_2_), 110.93, 113.40, 114.68, 116.96, 119.28, 121.62, 124.20, 124.67, 126.19, 129.59, 131.33, 132.68, 143.48, 150.87, 153.88, 161.55 (C = O indolin-2-one), 165.22 (C = O hydrazide), 167.89 (C = O ester); IR (KBr, *ν* cm^−1^) 3324 (NH) and 1745, 1715, 1698 (3 C = O); Analysis calculated for C_21_H_16_BrN_3_O_5_: C, 53.63; H, 3.43; N, 8.94; found C, 53.80; H, 3.42; N, 8.88.

###### 5.1.1.3.12. N'-(1-Benzyl-2-oxoindolin-3-ylidene)-5-bromobenzofuran-2-carbohydrazide 7e

Yellow powder (yield 80%), m.p. 248–250 °C; ^1^H NMR *ppm*: 5.01, 5.05 (2 s, 2H, *N*-CH_2_), 7.06 (t, 1H, Ar-H, *J* = 8.0 Hz), 7.17 (t, 1H, Ar-H, *J* = 8.0 Hz), 7.29– 7.32 (m, 4H, Ar-H), 7.42–7.48 (m, 2H, Ar-H), 7.69–7.73 (m, 1.5H, Ar-H), 7.77 (t, 1H, Ar-H, *J* = 8.0 Hz), 7.87, 8.02 (2 s, 1H, Ar-H), 8.09–8.12 (m, 1.5H, Ar-H), 12.01, 13.99 (2 s, 1H, NH); IR (KBr, *ν* cm^−1^) 3309 (NH) and 1712, 1701 (2 C = O); MS *m/z* [%]: 476 [M^+^+2, 16.41], 474 [M^+^, 21.15], 475 [100]; Analysis calculated for C_24_H_16_BrN_3_O_3_: C, 60.77; H, 3.40; N, 8.86; found C, 60.98; H, 3.38; N, 8.75.

###### 5.1.1.3.13. 5-Bromo-N'-(2-oxo-1-phenethylindolin-3-ylidene)benzofuran-2-carbohydrazide 7f

Yellow powder (yield 78%), m.p. 239–241 °C; ^1^H NMR *ppm*: 2.99 (br s, 2H, *N*-CH_2_-CH_2_), 4.03 (br s, 2H, *N*-CH_2_-CH_2_), 7.16–7.22 (m, 3H, Ar-H), 7.28–7.32 (m, 4H, Ar-H), 7.45 (t, 1H, Ar-H, *J* = 8.0 Hz), 7.46 (t, 2H, Ar-H, *J* = 8.0 Hz), 7.78 (d, 1H, Ar-H, *J* = 8.0 Hz), 7.85 (s, 1H, Ar-H), 8.09 (s, 1H, Ar-H), 11.97, 13.94 (2 s, 1H, NH); ^13 ^C NMR *δ ppm*: 33.43 (*N*-CH_2_-CH_2_), 41.36 (*N*-CH_2_-CH_2_), 110.86, 114.71, 116.95, 119.34, 120.21, 121.53, 123.75, 126.15, 127.02, 127.92, 128.93, 129.34, 129.59, 130.55, 131.24, 132.58, 135.38, 138.58, 141.28, 143.61, 153.87, 161.36 (C = O indolin-2-one), 165.06 (C = O hydrazide); IR (KBr, *ν* cm^−1^) 3356 (NH) and 1721, 1703 (2 C = O); Analysis calculated for C25H18BrN3O3: C, 61.49; H, 3.72; N, 8.60; found C, 61.63; H, 3.70; N, 8.66.

###### 5.1.1.3.14. 5-Bromo-N'-(5-bromo-1-isobutyl-2-oxoindolin-3-ylidene)benzofuran-2-carbohydrazide 7 g

Orange powder (yield 74%), m.p. 244–246 °C; ^1^H NMR *ppm*: 0.94 (d, 6H, –CH–(CH_3_)_2_, *J* = 7.6 Hz), 2.11–2.17 (m, 1H, *N*-CH_2_-CH), 3.51 (d, 2H, *N*-CH_2_, *J* = 8.0 Hz), 6.94–7.12 (m, 1H, Ar-H), 7.70 (d, 1H, Ar-H, *J* = 8.0 Hz), 7.73–7.80 (m, 2H, Ar-H), 8.10–8.18 (m, 2H, Ar-H), 8.35 (s, 1H, Ar-H), 12.12, 13.98 (2 s, 1H, NH); IR (KBr, *ν* cm^−1^) 3358 (NH) and 1724, 1700 (2 C = O); Analysis calculated for C21H17Br2N3O3: C, 48.58; H, 3.30; N, 8.09; found C, 48.73; H, 3.27; N, 8.14.

###### 5.1.1.3.15. N'-(1-Benzyl-5-bromo-2-oxoindolin-3-ylidene)-5-bromobenzofuran-2-carbohydrazide 7 h

Yellow powder (yield 81%), m.p. 236–237 °C; ^1^H NMR *ppm*: 5.00, 5.04 (2 s, 2H, *N*-CH_2_), 7.03 (d, 1H, Ar-H, *J* = 8.0 Hz), 7.29–7.32 (m, 1H, Ar-H), 7.35–7.39 (m, 2H, Ar-H), 7.42 (d, 2H, Ar-H, *J* = 8.0 Hz), 7.51–7.61 (m, 1H, Ar-H), 7.66–7.70 (m, 1H, Ar-H), 7.73–7.77 (m, 2H, Ar-H), 7.87, 8.13 (2 s, 1H, Ar-H), 8.07 (s, 1H, Ar-H), 12.19, 13.87 (2 s, 1H, NH); IR (KBr, *ν* cm^−1^) 3335 (NH) and 1731, 1708 (2 C = O); Analysis calculated for C_24_H_15_Br_2_N_3_O_3_: C, 52.11; H, 2.73; N, 7.60; found C, 51.86; H, 2.74; N, 7.69.

##### 5.1.1.4. Synthesis of 5-bromo-2-isocyanatobenzofuran 11

5-Bromobenzofuran-2-carbonyl chloride **9** (1 g, 3.8 mmol) was dissolved in dry acetone (30 ml), then added drop wisely to aqueous solution of equivalent amount of NaN_3_ at 0 °C over 30 min. The formed precipitate was filtrated under vacuum and washed with pet. ether, then heated without purification in dry toluene for 1 h to produce the intermediate **11**.

##### 5.1.1.5. Synthesis of hydrazones 12a-b

To hot stirred solution of the appropriate indolin-2-one **6c** and **6e** (6 mmol) in isopropyl alcohol (22 ml), hydrazine hydrate (0.50 ml, 15 mmol) was added. The reaction solution was heated under reflux for 3 h, then cold to room temperature. The formed solid was collected *via* filtration, washed with *n*-hexane and recrystallized from acetonitrile to afford the key intermediates hydrazones **12a-b** in a good yield (87–92%).

###### 5.1.1.5.1. 3-Hydrazono-1-isobutylindolin-2-one 12a

Yellow powder (yield 65%), m.p. 91–93 °C; ^1^H NMR *ppm*: 0.98 (d, 6H, -CH-(CH_3_)_2_, *J* = 6.8 Hz), 2.02–2.12 (m, 1H, *N*-CH_2_-CH), 3.55 (d, 2H, *N*-CH_2_, *J* = 7.6 Hz), 7.02 (t, 1H, Ar-H, *J* = 7.6 Hz), 7.08 (d, 1H, Ar-H, *J* = 8.0 Hz), 7.21 (t, 1H, Ar-H, *J* = 8.0 Hz), 7.41 (d, 1H, Ar-H, *J* = 7.6 Hz), 9.67 (d, 1H, NH_2_, D_2_O exchangeable, *J* = 14.8 Hz), 10.54 (d, 1H, NH_2_, D_2_O exchangeable, *J* = 14.8 Hz); ^13 ^C NMR *δ ppm*: 20.45 (–CH–(CH_3_)_2_), 27.39 (*N–*CH_2_–CH), 46.40 (*N–*CH_2_), 109.59, 117.73, 121.83, 122.23, 125.66, 127.45, 140.07, 161.49 (C = O indolin-2-one); Analysis calculated for C_12_H_15_N_3_O: C, 66.34; H, 6.96; N, 19.34; found C, 66.53; H, 6.91; N, 19.39.

##### 5.1.1.6. Synthesis of target compounds 13a-b

To the previously prepared solution of isocyanate **11**, equimolar amount of hydrazone **12a–b** was added. The reaction mixture was heated for 4 h, the formed precipitate was filtrated while hot, washed with diethyl ether and recrystallized from DMF/EtOH mixture to produce targeted compounds **13a–b**, respectively.

###### 5.1.1.6.1. N-(5-Bromobenzofuran-2-yl)-2–(1-isobutyl-2-oxoindolin-3-ylidene)hydrazine-1-carboxamide 13a

Yellow powder (yield 73%), m.p. 208–210 °C; ^1^H NMR *ppm*: 0.94 (d, 6H, –CH–(CH_3_)_2_, *J* = 8.0 Hz), 2.06–2.13 (m, 1H, *N***–**CH_2_**–**CH), 3.57 (d, 2H, *N*-CH_2_, *J* = 7.6 Hz), 6.55 (s, 1H, Ar-H), 6.63 (s, 0.5H, Ar-H), 7.15–7.21 (m, 1H, Ar-H), 7.28–7.32 (m, 1.5H, Ar-H), 7.41–7.47 (m, 2H, Ar-H), 7.70–7.74 (m, 2H, Ar-H), 10.12 (s, 1H, NH urea), 11.33 (s, 1H, NH urea); ^13 ^C NMR *δ ppm*: 20.44 (–CH–(CH_3_)_2_), 27.28 (*N–*CH_2_–CH), 66.82 (*N*–CH_2_), 110.60, 112.52, 116.02, 119.90, 121.04, 122.52, 123.30, 125.07, 131.39, 132.27, 133.49, 143.34, 148.57, 148.99, 150.31, 151.01, 161.27 (C = O indolin-2-one), 165.09 (C = O hydrazide); IR (KBr, *ν* cm^−1^) 3345, 3240 (2NH) and 1730, 1711 (2 C = O); MS *m/z* [%]: 457 [M^+^+2, 27.45], 455 [M^+^, 29.72], 414 [100]; Analysis calculated for C_21_H_19_BrN_4_O_3_: C, 55.40; H, 4.21; N, 12.31; found C, 55.59; H, 4.16; N, 12.42.

###### 5.1.1.6.2. 2–(1-Benzyl-2-oxoindolin-3-ylidene)-N-(5-bromobenzofuran-2-yl)hydrazine-1-carboxamide 13 b

Yellow powder (yield 68%), m.p. 217–219 °C; ^1^H NMR *ppm*: 4.98 (s, 2H, *N*-CH_2_), 6.53 (s, 1H, Ar-H), 6.61 (s, 1H, Ar-H), 7.02 (d, 1H, Ar-H, *J* = 8.0 Hz), 7.11 (t, 1H, Ar-H, *J* = 8.0 Hz), 7.25–7.30 (m, 2H, Ar-H), 7.31–7.38 (m, 3H, Ar-H), 7.41–7.45 (m, 2H, Ar-H), 7.68–7.71 (m, 2H, Ar-H), 10.10 (s, 1H, NH urea), 11.38 (s, 1H, NH urea); ^13 ^C NMR *δ ppm*: 42.51 (CH_2_), 115.57, 119.67, 120.62, 122.17, 123.04, 124.56, 124.73, 127.43, 127.63, 128.74, 130.81, 131.68, 132.92, 135.84, 142.18, 148.06, 148.21, 148.48, 149.75, 150.56, 150.69, 160.57 (C = O indolin-2-one), 165.06 (C = O hydrazide); IR (KBr, *ν* cm^−1^) 3334, 3270 (2NH) and 1728, 1707 (2 C = O); Analysis calculated for C_24_H_17_BrN_4_O_3_: C, 58.91; H, 3.50; N, 11.45; found C, 59.22; H, 3.53; N, 11.52.

### 5.2. Biological evaluations

All experimental procedures utilised in the biological assays herein conducted were performed as reported earlier; anti-proliferation^115^, cell cycle^116–117^, Annexin V-FITC Apoptosis^118^ and CDK2 kinase^119^ assays, and have been provided in the Supplementary Materials.

### 5.3. Molecular docking study

All the molecular modelling simulations were performed using Molecular Operating Environment (MOE, 2010.10) software. All minimizations were carried out with MOE until an RMSD gradient of 0.05 kcal·mol^−1 ^Å^−1^ with MMFF94× force field and the partial charges were automatically calculated. The X-ray crystallographic structure of CDK2 co-crystallized with an oxindole derivative (IC_50_ = 60 nM) as inhibitor (PDB ID: 1FVT)^109^ and of GSK-3β co-crystallized with the oxindole derivative Indirubin-3′-monoxime (IC_50_ = 22 nM) as inhibitor (PDB ID: 1Q41)^110^ were downloaded from the protein data bank^120^. The selection of these two protein structures specifically attributed to their co-crystallization with potent CDK2 and GSK-3β oxindole-based inhibitors, respectively.

For the CDK2 protein structure (PDB ID: 1FVT); water molecules were first removed, as for the GSK-3β protein structure (PDB ID: 1Q41); Chain B and water molecules which are not involved in binding were first removed, whereas, two conservative water molecules near chain A Thr138 that serve a functional role in the co-crystalized inhibitor binding were kept^110^. Then, the proteins were prepared for the docking study using *LigX* protocol in MOE with default options. The co-crystalized ligands were used to define the active site for docking. Triangle Matcher placement method and London dG scoring function were used for docking. Self-docking of the co-crystallized ligands in the active site of the kinase domains was first performed to validate the used docking protocol giving a docking pose with an energy score (S) = −10.63 kcal/mol and an RMSD of 0.894 Å in CDK2 (PDB ID: 1FVT) and energy score (S) = −12.00 kcal/mol and an RMSD of 0.471 Å in GSK-3β (PDB ID: 1Q41), Figure 7 and Table 5.

The validated docking protocols were then used to study the ligand-protein interactions of the newly synthesised compounds in the active site of the target kinases to predict their binding mode and establish their structure activity relationship (SAR) to rationalise their binding affinity.

## Supplementary Material

Supplemental Material
