# Supplementary material for: Novel oxindole/benzofuran hybrids as potential dual CDK2/GSK-3β inhibitors targeting breast cancer: design, synthesis, biological evaluation, and in silico studies
Source: J Enzyme Inhib Med Chem. 2020 Dec 16;36(1):270–85. doi: 10.1080/14756366.2020.1862101 (PMC7751407; doi:10.1080/14756366.2020.1862101)

Supporting Information

Novel Oxindole/Benzofuran Hybrids as Potential Dual CDK2/GSK-3β Inhibitors Targeting Breast Cancer: Design, Synthesis, Biological Evaluation, and *In Silico* Studies

Wagdy M. Eldehna*, Sara T. Al-Rashood, Razan O. Eskandrani, Amal Alharbi, Tarfah Al-Warhi, Ahmed M. El Kerdawy

Tables of Contents

**1.**	Anti-proliferative Activity	3
**2.**	CDK2/GSK-3β Kinase Inhibitory Activity	3
**3.**	Cell Cycle Analysis	4
**4.**	AnnexinV-FITC/PI Apoptosis Assay	4
**5.**	2D Diagrams for hybrids **5** showing their interactions with the CDK2 binding site	5-7
**6.**	2D Diagrams for hybrids **5** showing their interactions with the GSK-3β binding site	8-10
**7.**	2D Representations of the interactions and superimposition of the docking pose and the co-crystallized pose	11
**8.**	NMR Spectra	12-35

1. Anti-proliferative activity toward human breast cell lines

The two examined human breast cancer cell lines (T-47D and MCF-7) have been obtained from American Type Culture Collection (ATCC). Cells lines were maintained as monolayers in Dulbecco's Modified Eagle's Medium (DMEM) supplemented with 10% FBS, 2 mM L-glutamine, 100 U/ml penicillin and 100µg/ml streptomycin sulfate. Cells were sub-cultured with trypsine /EDTA solution, counted with haemocytometer and plated onto 96-well plates (5000 cells/well) and left overnight to form a semi-confluent monolayer. Cell monolayers were treated in quadrates with vehicle (DMSO, 0.1% v/v), test samples or Adriamycin as positive control for an exposure time of 48 h. At the end of exposure, MTT solution in PBS (5 mg/ml) was then added to all well including no cell blank and left to incubate for 90 min. The formation of formazan crystals were visually confirmed using phase contract microscopy. DMSO (100 µl/well) was added to dissolve the formazan crystals with shaking for 10 min after which the absorbance was read at 590 nm against no cell blanks on a FLuo Star Optima microplate reader (BMG technologies, Germany). Cell proliferation was calculated comparing the OD values of the DMSO control wells and those of the samples represented as % proliferation to the control. Dose-response experiment was performed on samples producing > or =50% loss of cell proliferation using five serial 2-fold dilutions (50, 25, 12.5, 6.25 and 3.125 µM) of the sample. IC_{50} values (concentration of sample causing 50% loss of cell proliferation of the vehicle control) were calculated using non-linear regression curve fitting of the dose response plots on GraphPad Prism V.6.0 software.

2. CDK2/GSK-3β Kinase Inhibitory Activities

The CDK2 and GSK-3β inhibitory activities were determined for hybrids **5a-g** using the CDK2 Kinase Enzyme System (catalog No. V2971) (Promega, Milan, Italy), and GSK3β Assay Kit (catalog No. 79700) (BPS Biosciences, San Diego, CA, USA), according to the manufacturer's instructions.

3. Cell Cycle Analysis

Breast cancer MCF-7 cells were treated with hybrids (**5d**, **5e** and **5f**) for 24 h (at their IC_{50} concentration), and then cells were washed twice with ice-cold phosphate buffered saline (PBS). Subsequently, the treated cells were collected by centrifugation, fixed in ice-cold 70% (*v*/*v*) ethanol, washed with PBS, re-suspended with 100 μg/mL RNase, stained with 40 μg/mL PI, and analyzed by flow cytometry using FACS Calibur (Becton Dickinson, BD, Franklin Lakes, NJ, USA). The cell cycle distributions were calculated using CellQuest software 5.1 (Becton Dickinson).

4. Annexin V-FITC Apoptosis Assay

Phosphatidylserine externalization was assayed using Annexin V-FITC/PI apoptosis detection kit (BD Biosciences, USA) according to the manufacturer's instructions. Breast cancer MCF-7 cells were cultured to a monolayer then treated with hybrids (**5d, 5e and 5f**) at their IC_{50} concentration. Briefly, cells were then harvested *via* trypsinization, and rinsed twice in PBS followed by binding buffer. Moreover, cells were re-suspended in 100 μL of binding buffer with the addition of 1 μL of FITC-Annexin V followed by an incubation period of 30 min at 4 °C. Cells were then rinsed in binding buffer and resuspended in 150 μL of binding buffer with the addition of 1 μL of DAPI (1 μg/μL in PBS). Cells were then analyzed using the flow cytometer BD FACS Canto II and the results were interpreted with FlowJo7.6.4 software (Tree Star, Ashland, OR, USA).

5. 2D Diagrams for hybrids 5 showing their interactions with the CDK2 binding site

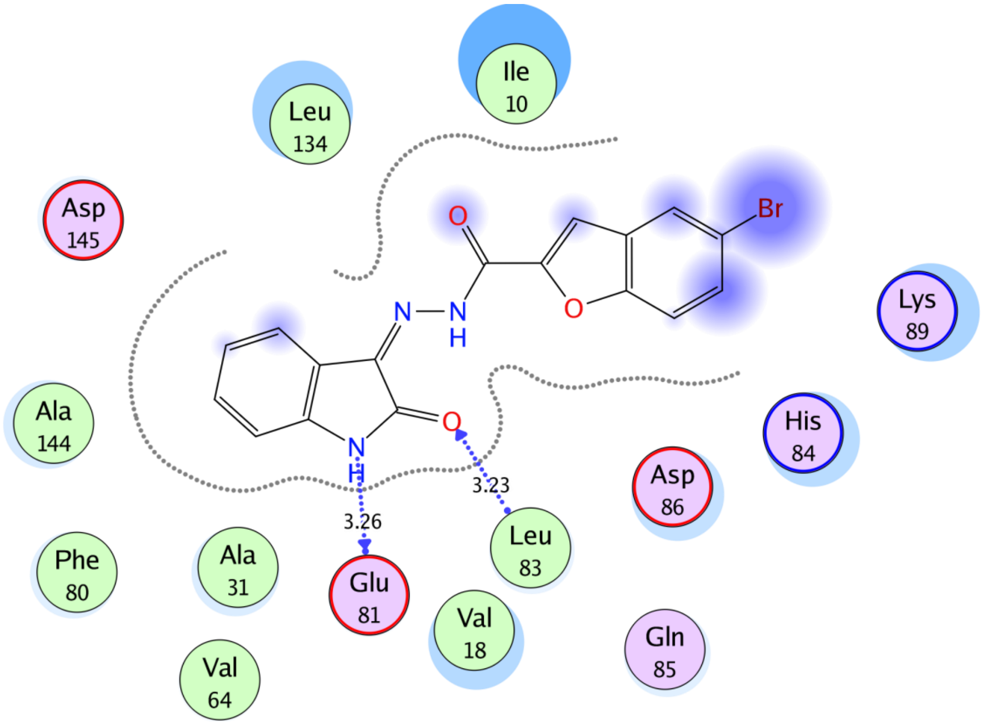

Figure S1. 2D diagram for hybrid **5a** showing its interaction with the CDK2 binding site.

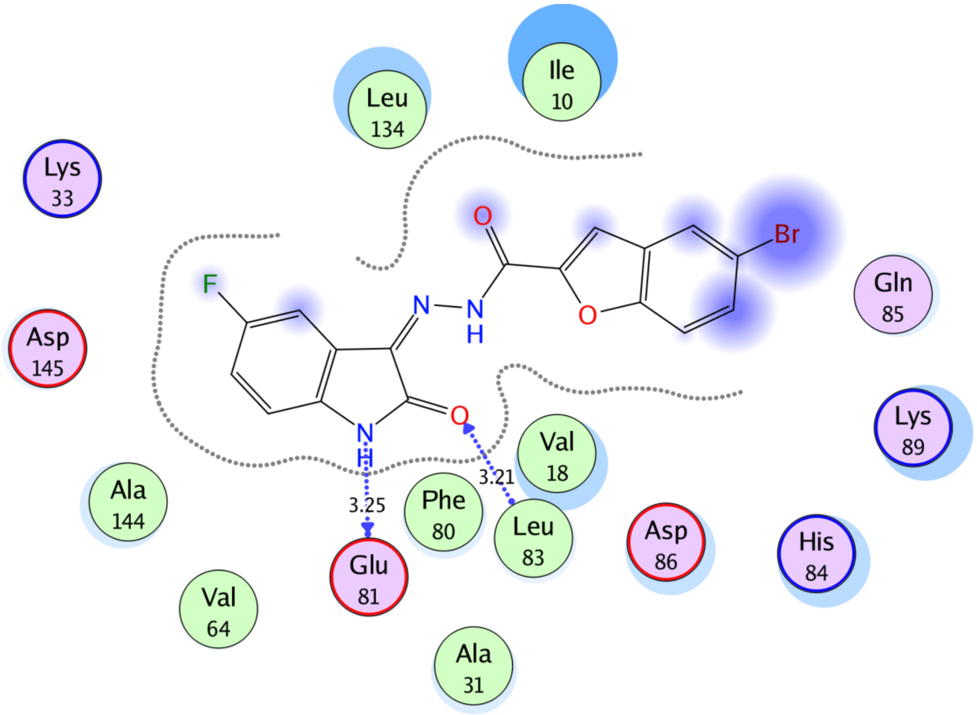

Figure S2. 2D diagram for hybrid **5b** showing its interaction with the CDK2 binding site.

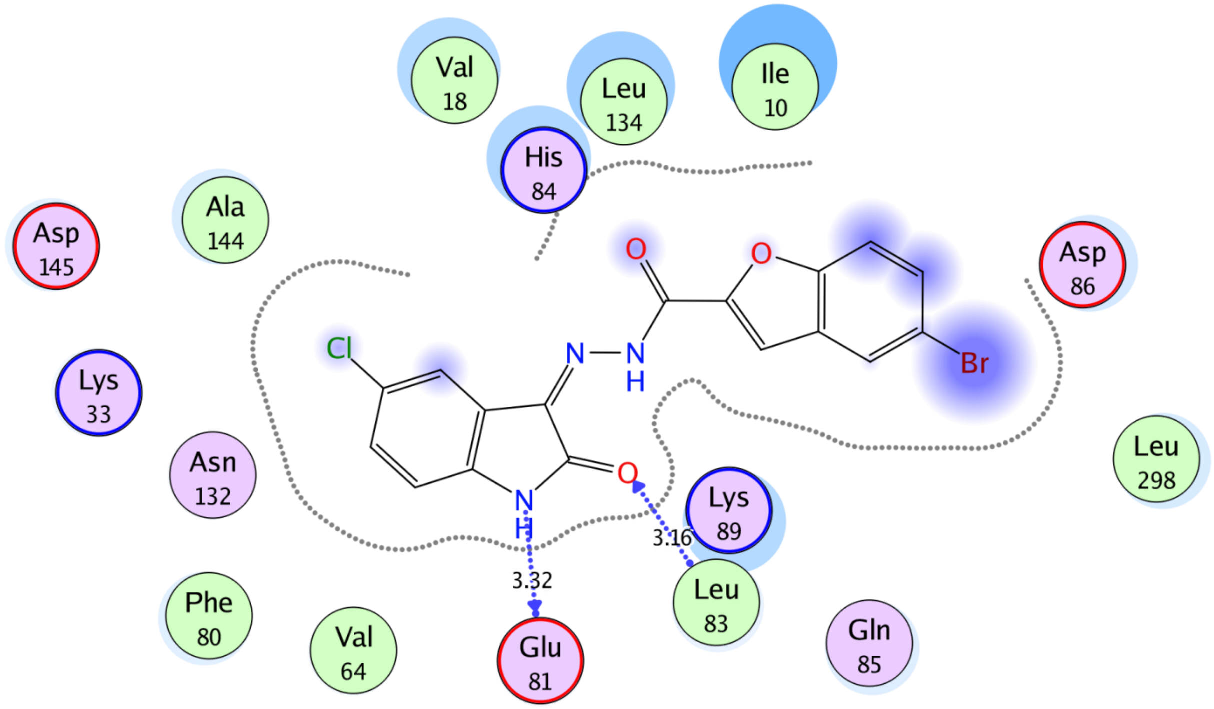

Figure S3. 2D diagram for hybrid **5c** showing its interaction with the CDK2 binding site.

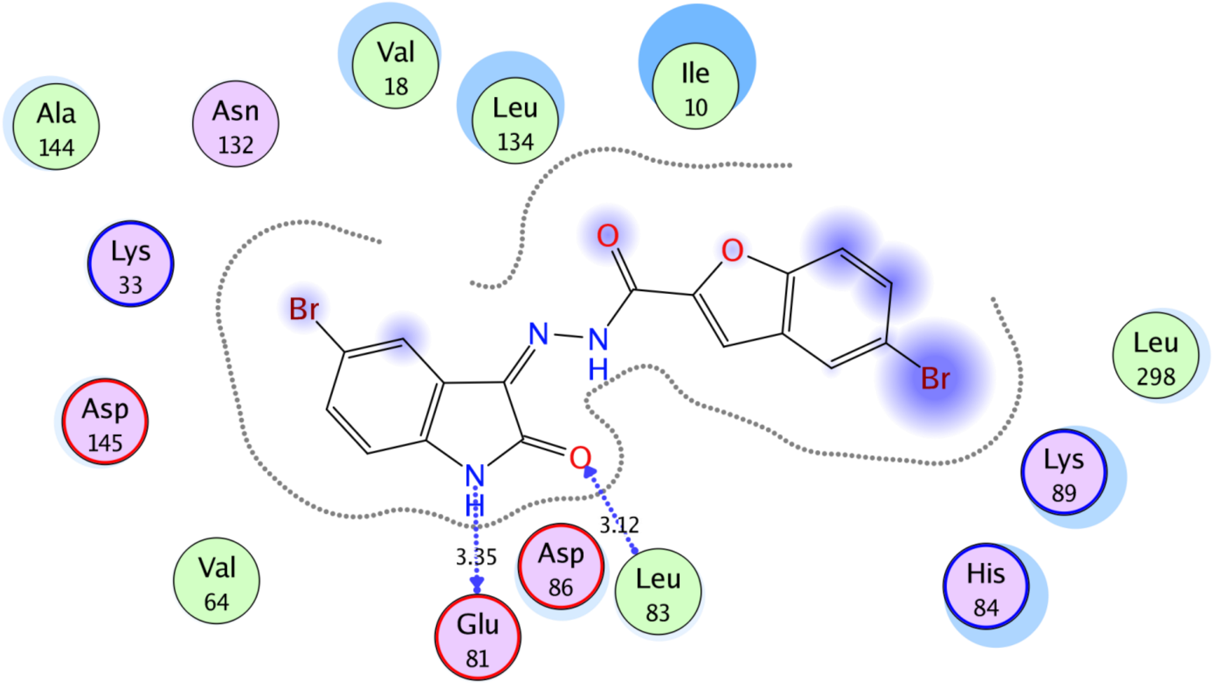

Figure S4. 2D diagram for hybrid **5d** showing its interaction with the CDK2 binding site.

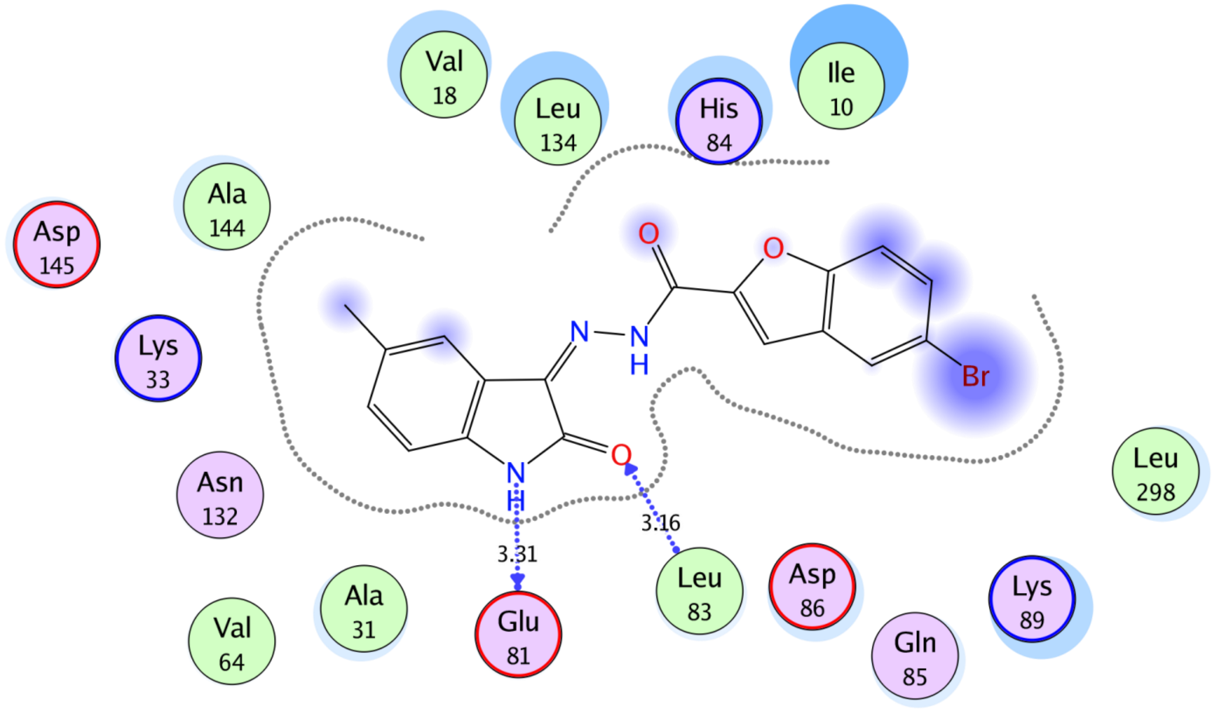

Figure S5. 2D diagram for hybrid **5e** showing its interaction with the CDK2 binding site.

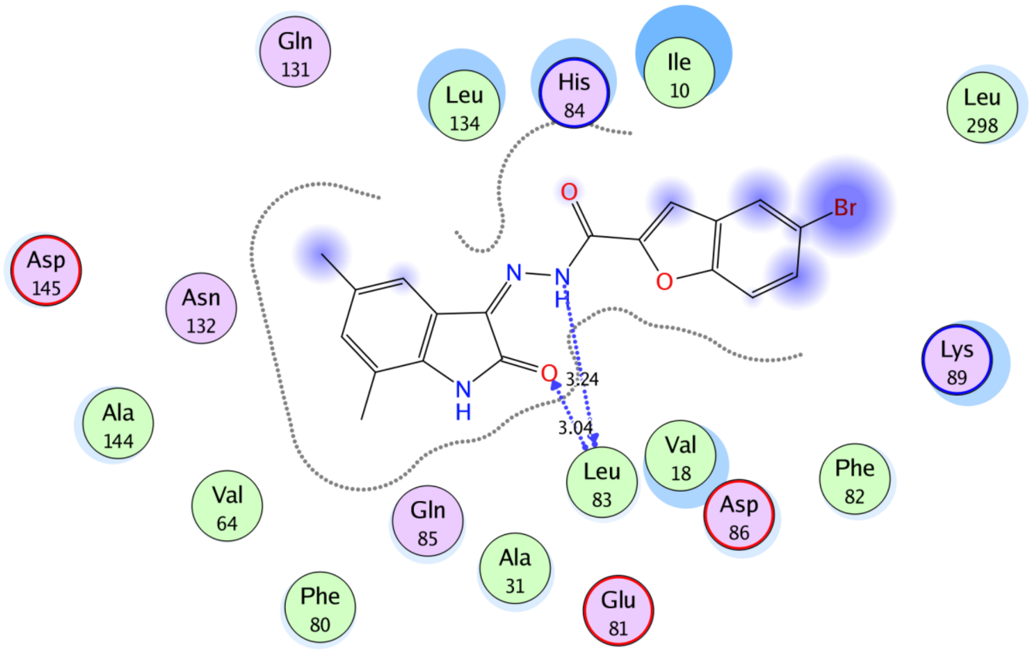

Figure S6. 2D diagram for hybrid **5g** showing its interaction with the CDK2 binding site.

6. 2D Diagrams for hybrids 5 showing their interactions with the GSK-3β binding site

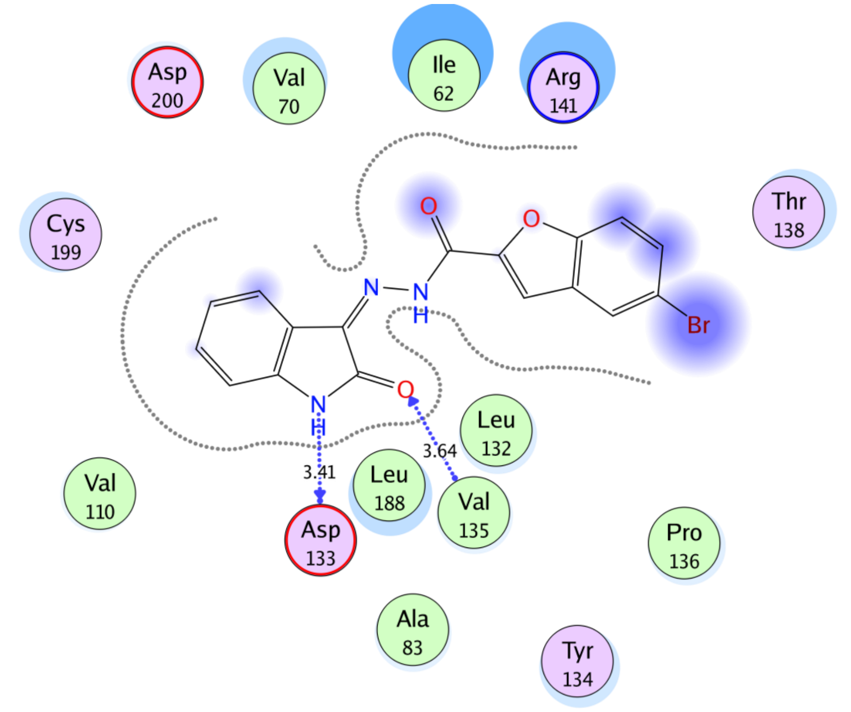

Figure S7. 2D diagram for hybrid **5a** showing its interaction with the GSK-3β binding site.

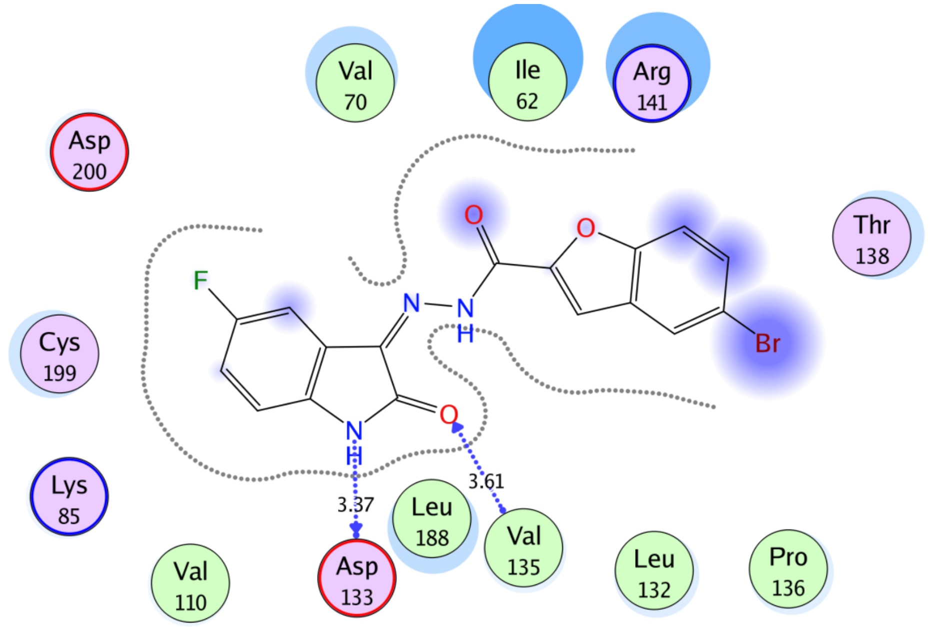

Figure S8. 2D diagram for hybrid **5b** showing its interaction with the GSK-3β binding site.

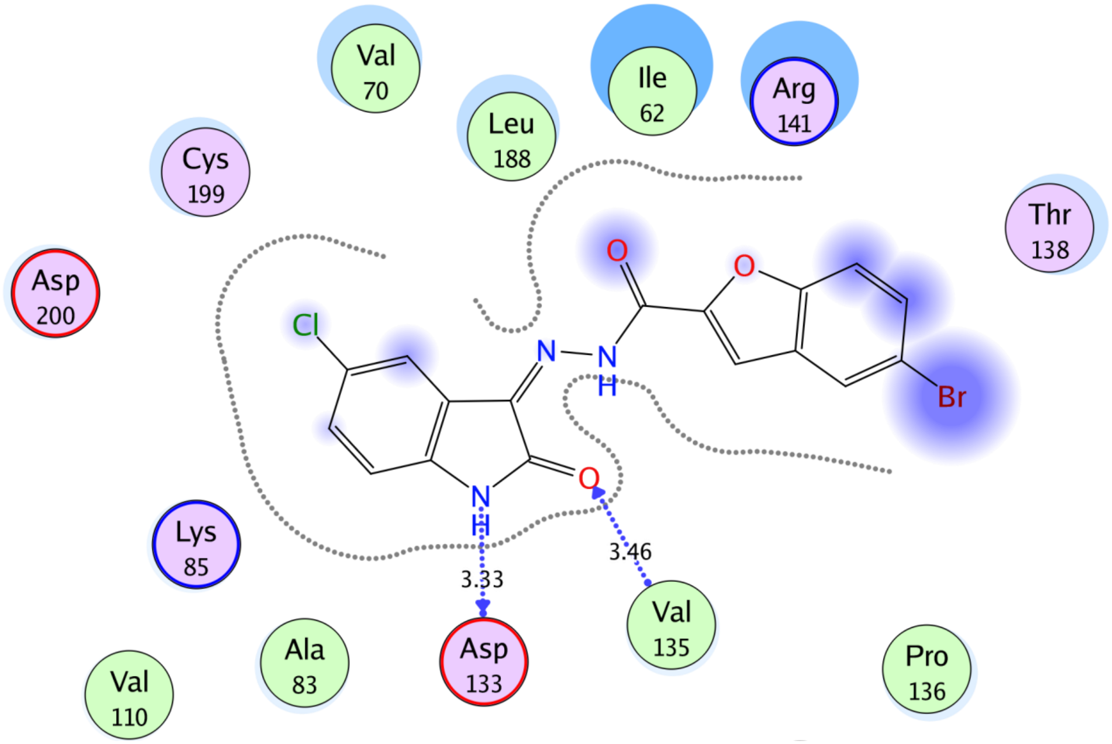

Figure S9. 2D diagram for hybrid **5c** showing its interaction with the GSK-3β binding site.

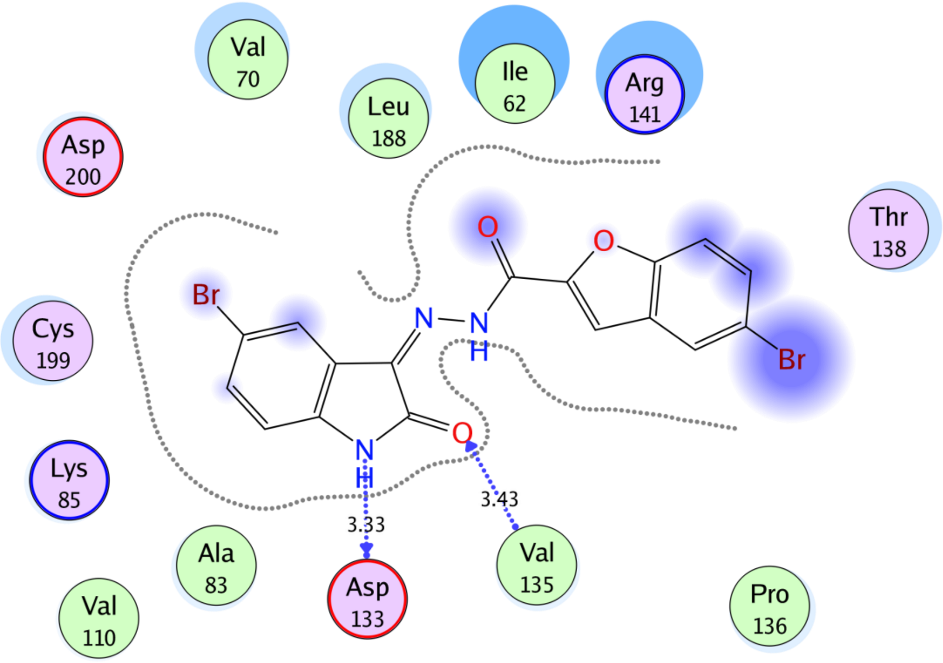

Figure S10. 2D diagram for hybrid **5d** showing its interaction with the GSK-3β binding site.

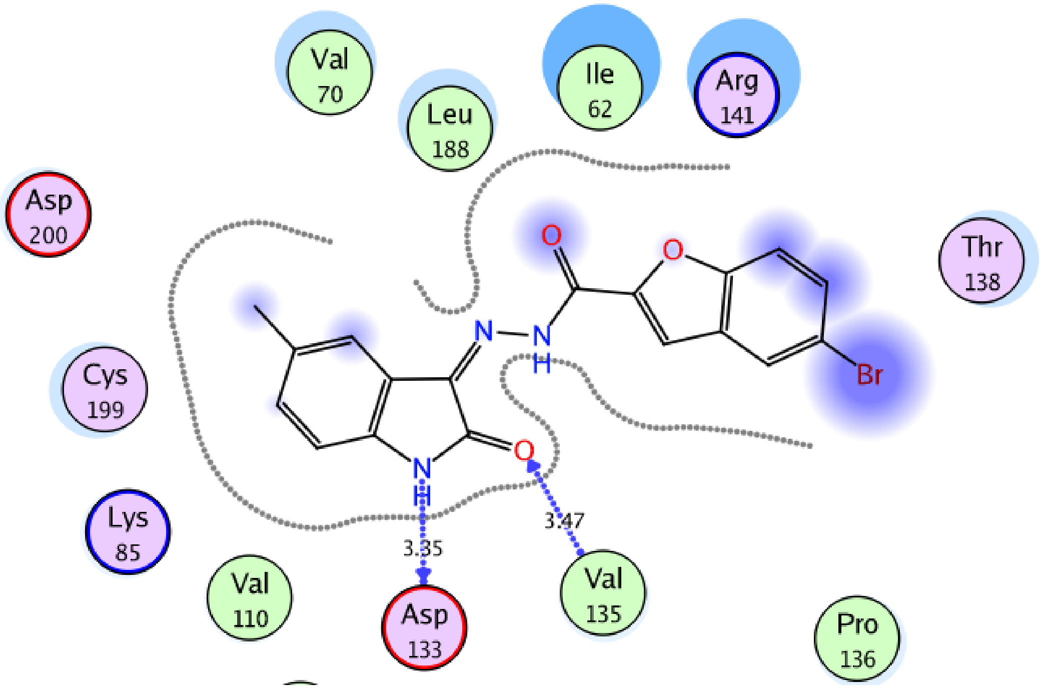

Figure S11. 2D diagram for hybrid **5e** showing its interaction with the GSK-3β binding site.

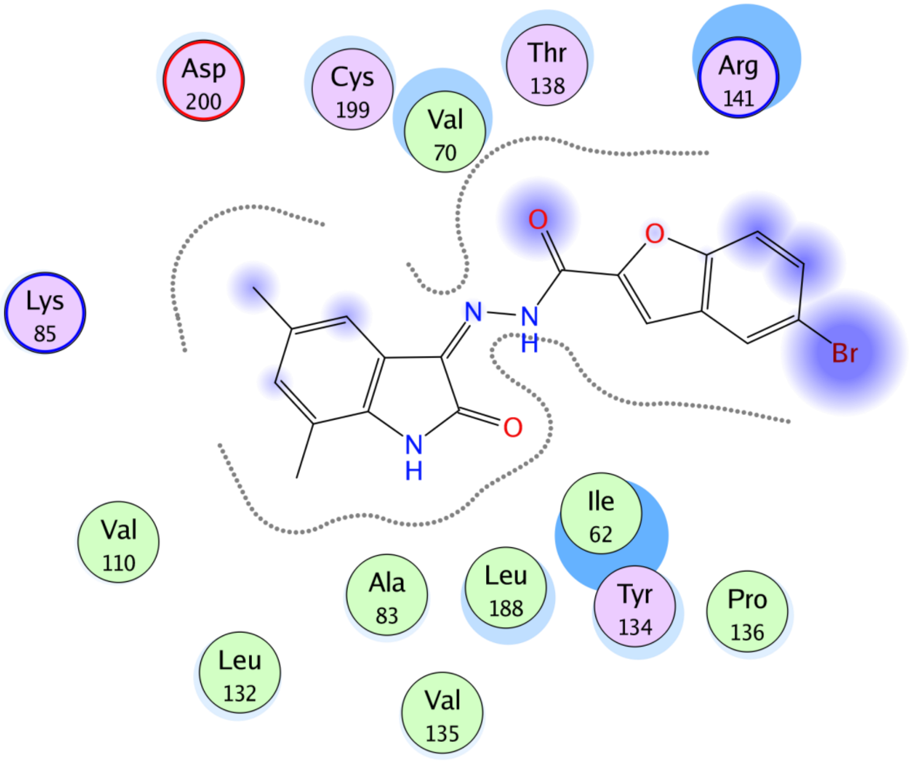

Figure S12. 2D diagram for hybrid **5g** showing its interaction with the GSK-3β binding site.

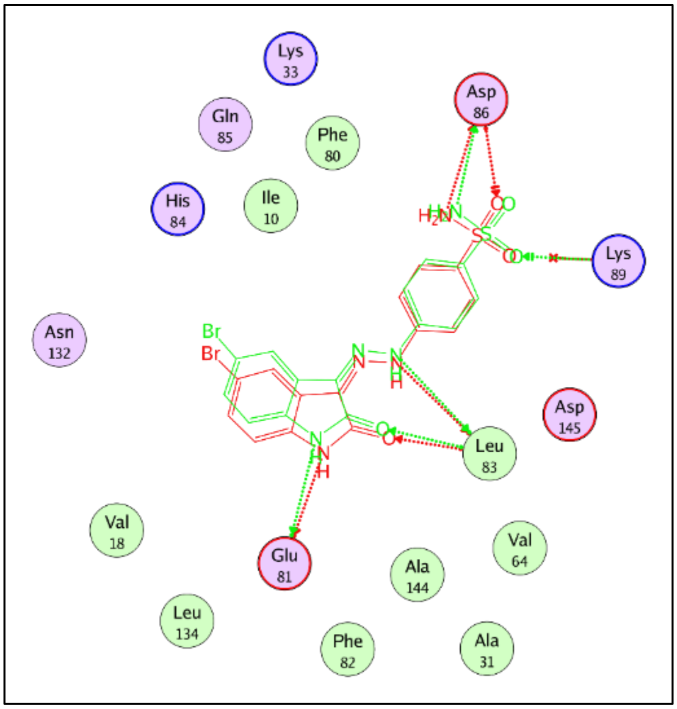

Figure S13. 2D representations of the interactions and superimposition of the docking pose (green) and the co-crystallized pose (red) of the oxindole derivative in the CDK2 active site, respectively, with RMSD of 0.894Å.

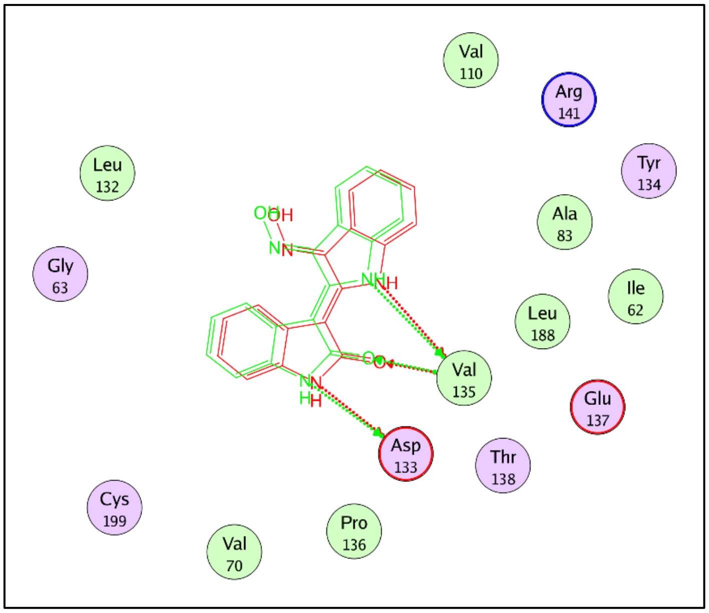

Figure S14. 2D representations of the interactions and superimposition of the docking pose (green) and the co-crystallized pose (red) of Indirubin-3'-monoxime in the GSK-3β active site, respectively, with RMSD of 0.471Å.

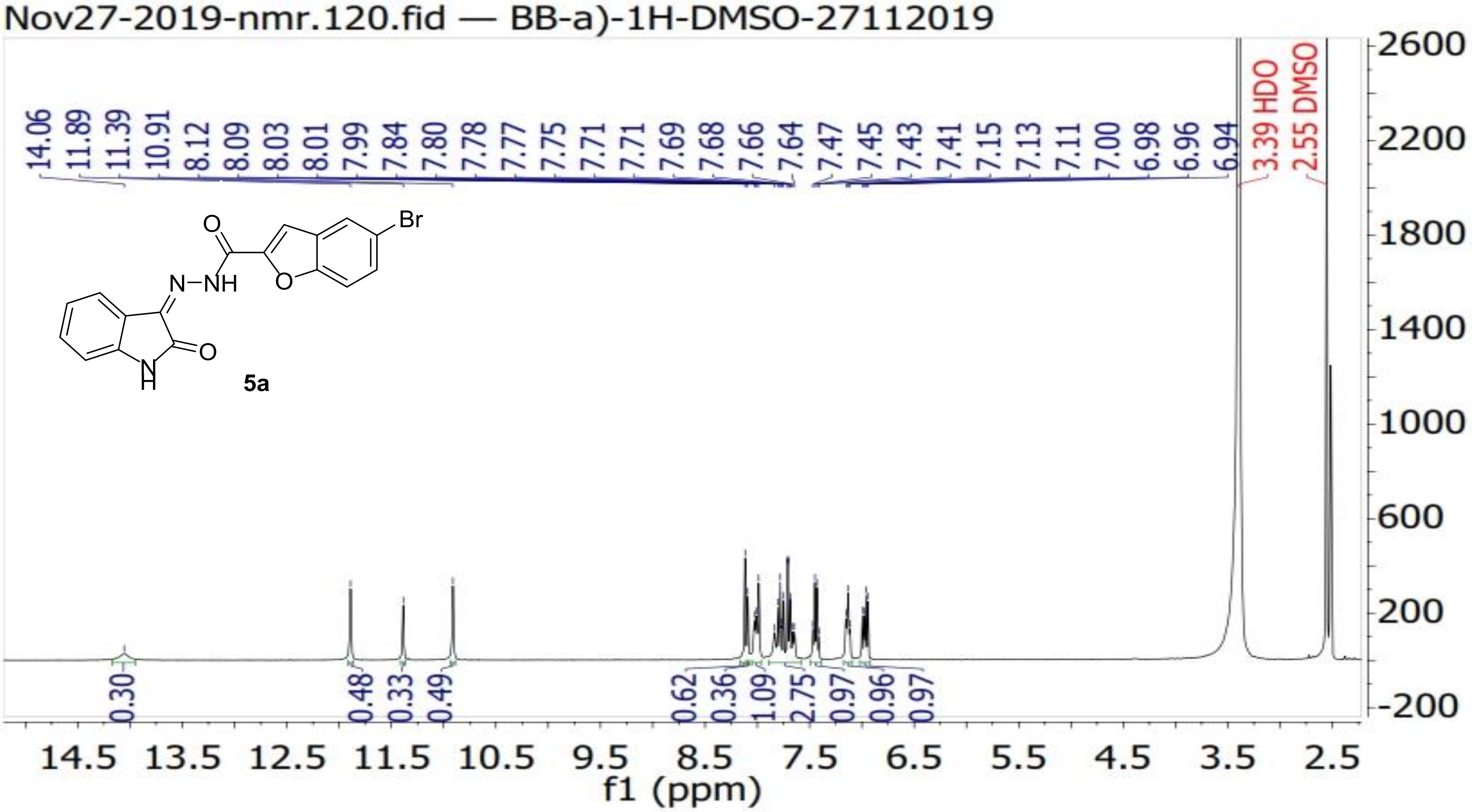
Nov27-2019-nmr.120.fid — BB-a)-1H-DMSO-27112019
14.06 11.89 11.39 10.91 8.12 8.09 8.03 8.01 7.99 7.84 7.80 7.78 7.77 7.75 7.71 7.71 7.69 7.68 7.66 7.64 7.47 7.45 7.43 7.41 7.15 7.13 7.11 7.00 6.98 6.96 6.94
3.39 HDO
2.55 DMSO
Br
O
N–NH
O
N
H
O
5a
0.30 0.48 0.33 0.49 0.62 0.36 1.09 2.75 0.97 0.96 0.97
14.5 13.5 12.5 11.5 10.5 9.5 8.5 7.5 6.5 5.5 4.5 3.5 2.5
f1 (ppm)
2600 2200 1800 1400 1000 600 200 -200

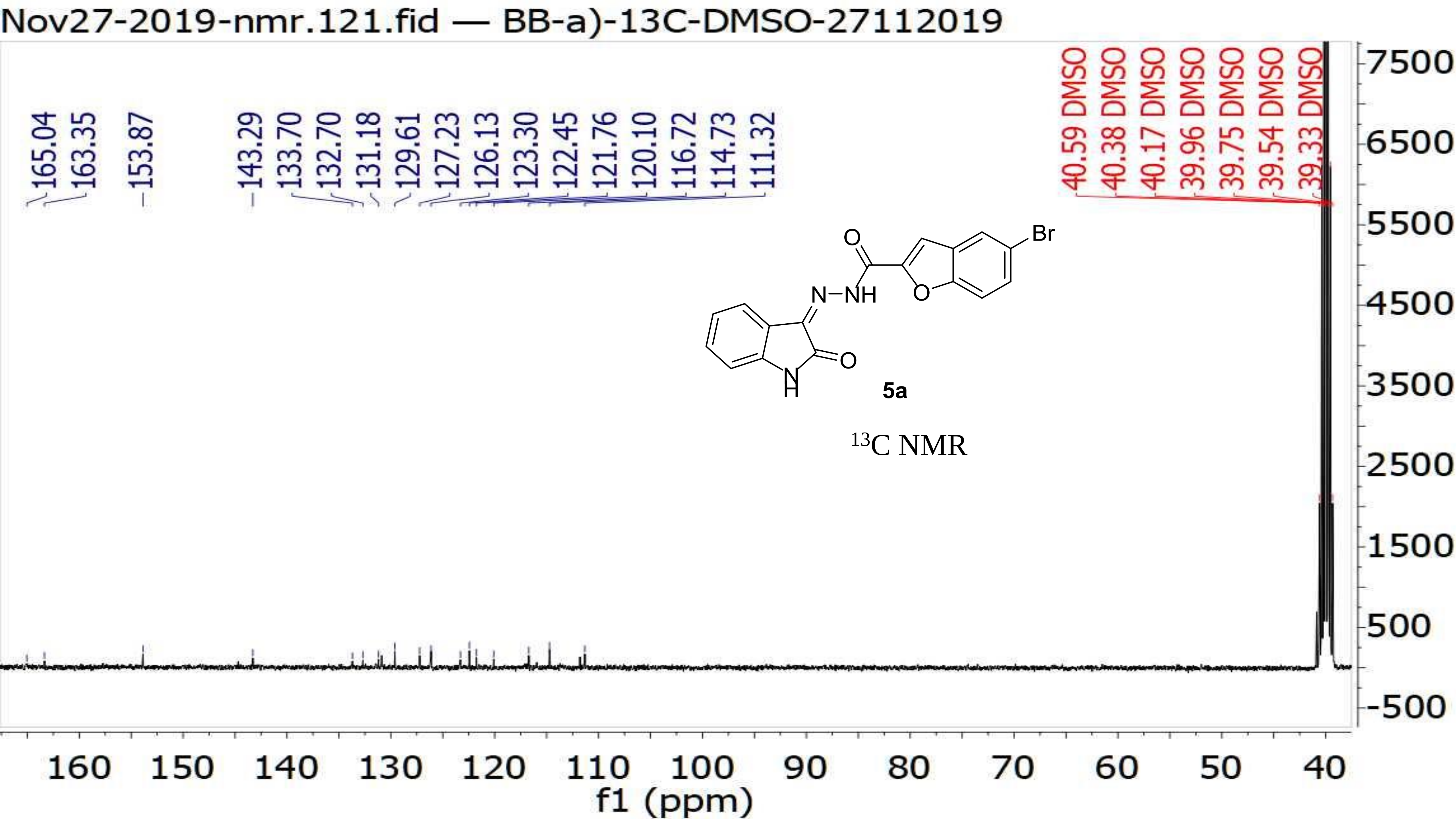
Nov27-2019-nmr.121.fid — BB-a)-13C-DMSO-27112019
165.04
163.35
153.87
143.29
133.70
132.70
131.18
129.61
127.23
126.13
123.30
122.45
121.76
120.10
116.72
114.73
111.32
40.59 DMSO
40.38 DMSO
40.17 DMSO
39.96 DMSO
39.75 DMSO
39.54 DMSO
39.33 DMSO
O
Br
N
NH
O
O
N
H
5a
13C NMR
7500
6500
5500
4500
3500
2500
1500
500
-500
160
150
140
130
120
110
100
90
80
70
60
50
40
f1 (ppm)

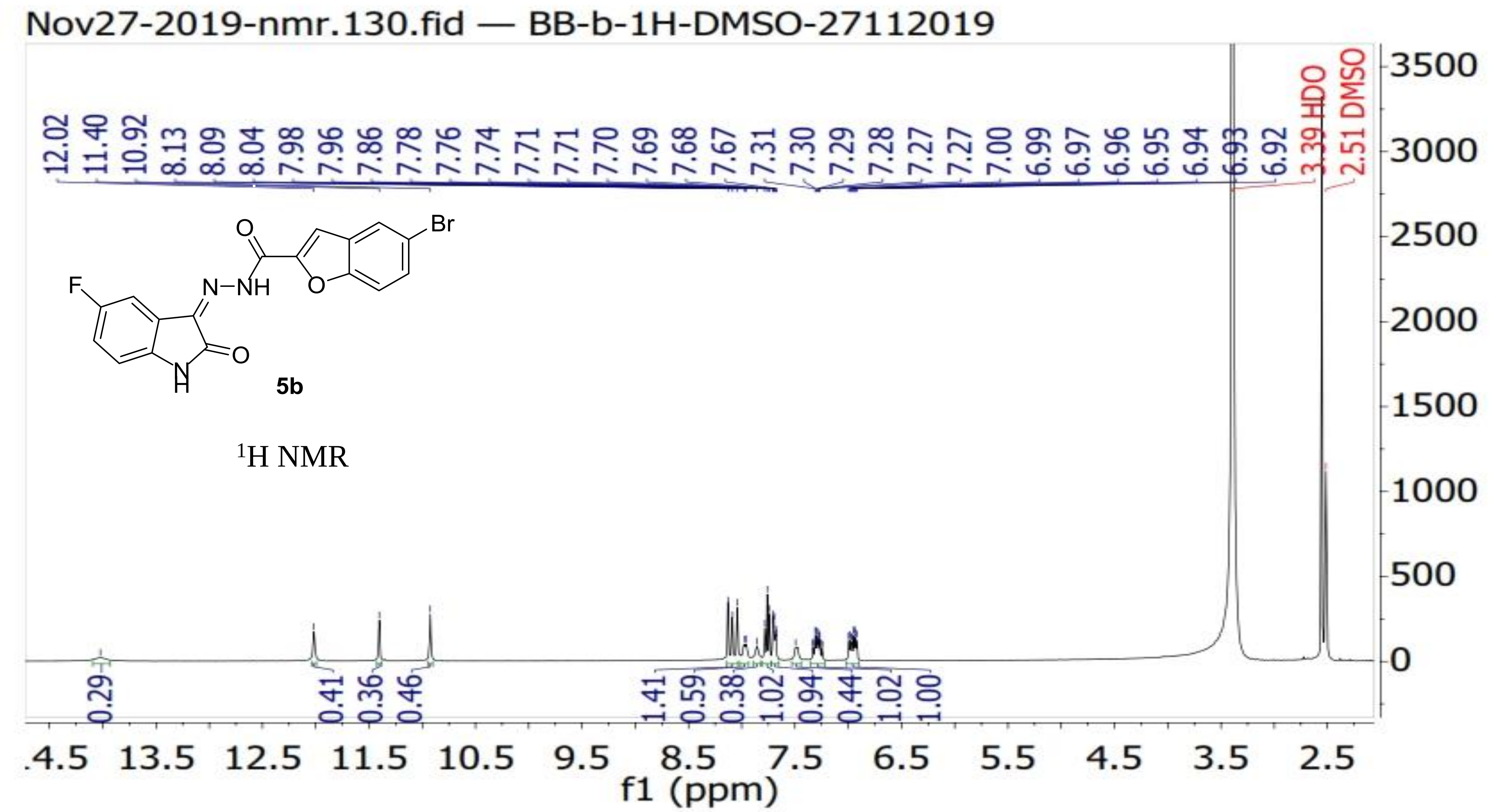
Nov27-2019-nmr.130.fid — BB-b-1H-DMSO-27112019
12.02 11.40 10.92 8.13 8.09 8.04 7.98 7.96 7.86 7.78 7.76 7.74 7.71 7.71 7.70 7.69 7.68 7.67 7.31 7.30 7.29 7.28 7.27 7.27 7.00 6.99 6.97 6.96 6.95 6.94 6.93 6.92
3.39 HDO
2.51 DMSO
Br
O
F
N–NH
O
O
N
H
5b
¹H NMR
0.29 0.41 0.36 0.46 1.41 0.59 0.38 1.02 0.94 0.44 1.02 1.00
3500
3000
2500
2000
1500
1000
500
0
.4.5 13.5 12.5 11.5 10.5 9.5 8.5 7.5 6.5 5.5 4.5 3.5 2.5
f1 (ppm)

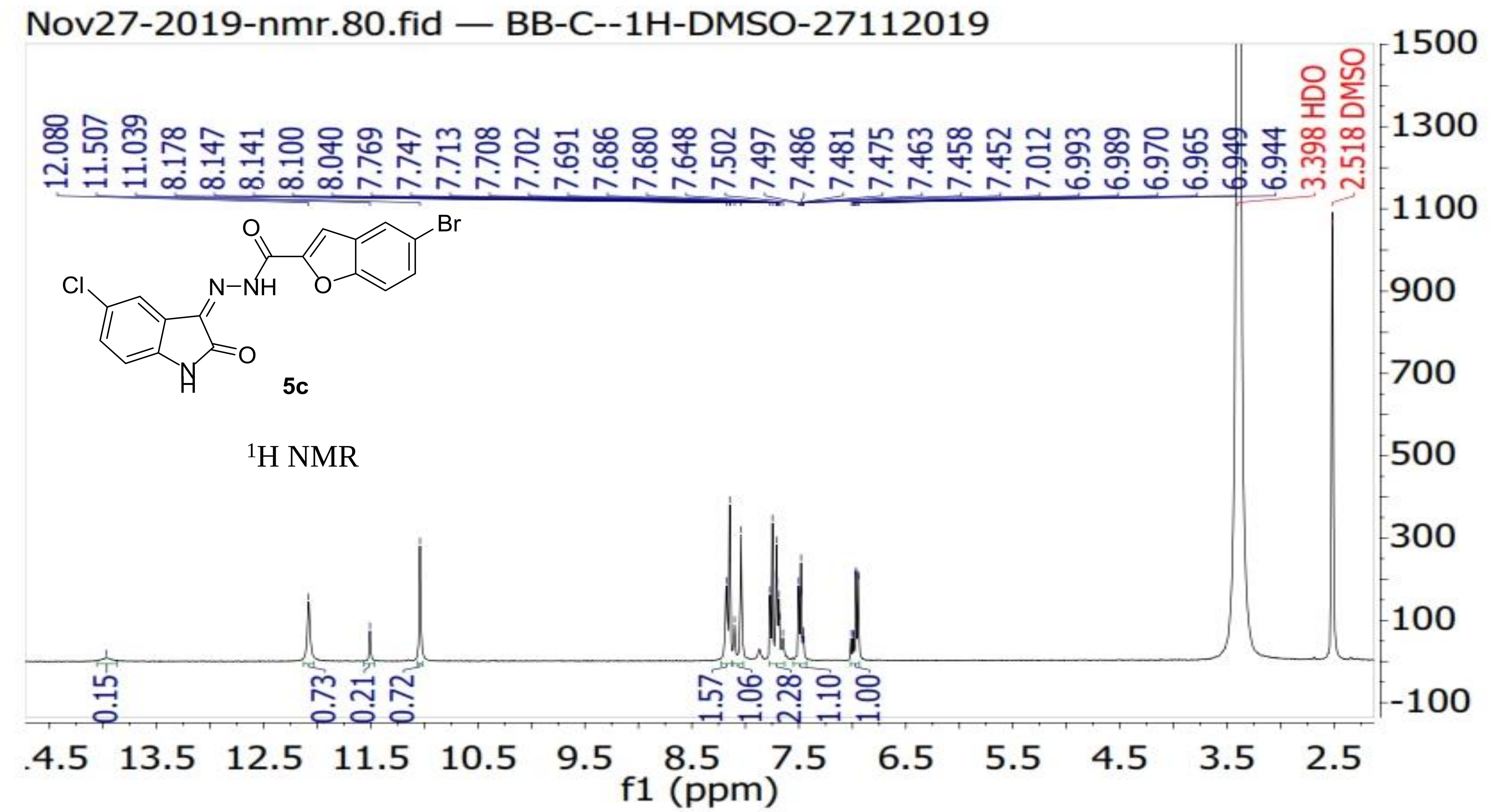

Nov27-2019-nmr.80.fid — BB-C--1H-DMSO-27112019
12.080 11.507 11.039 8.178 8.147 8.141 8.100 8.040 7.769 7.747 7.713 7.708 7.702 7.691 7.686 7.680 7.648 7.502 7.497 7.486 7.481 7.475 7.463 7.458 7.452 7.012 6.993 6.989 6.970 6.965 6.949 6.944
3.398 HDO
2.518 DMSO
Br
Cl
N–NH
5c
1H NMR
0.15 0.73 0.21 0.72 1.57 1.06 2.28 1.10 1.00
1500 1300 1100 900 700 500 300 100 -100
.4.5 13.5 12.5 11.5 10.5 9.5 8.5 7.5 6.5 5.5 4.5 3.5 2.5
f1 (ppm)

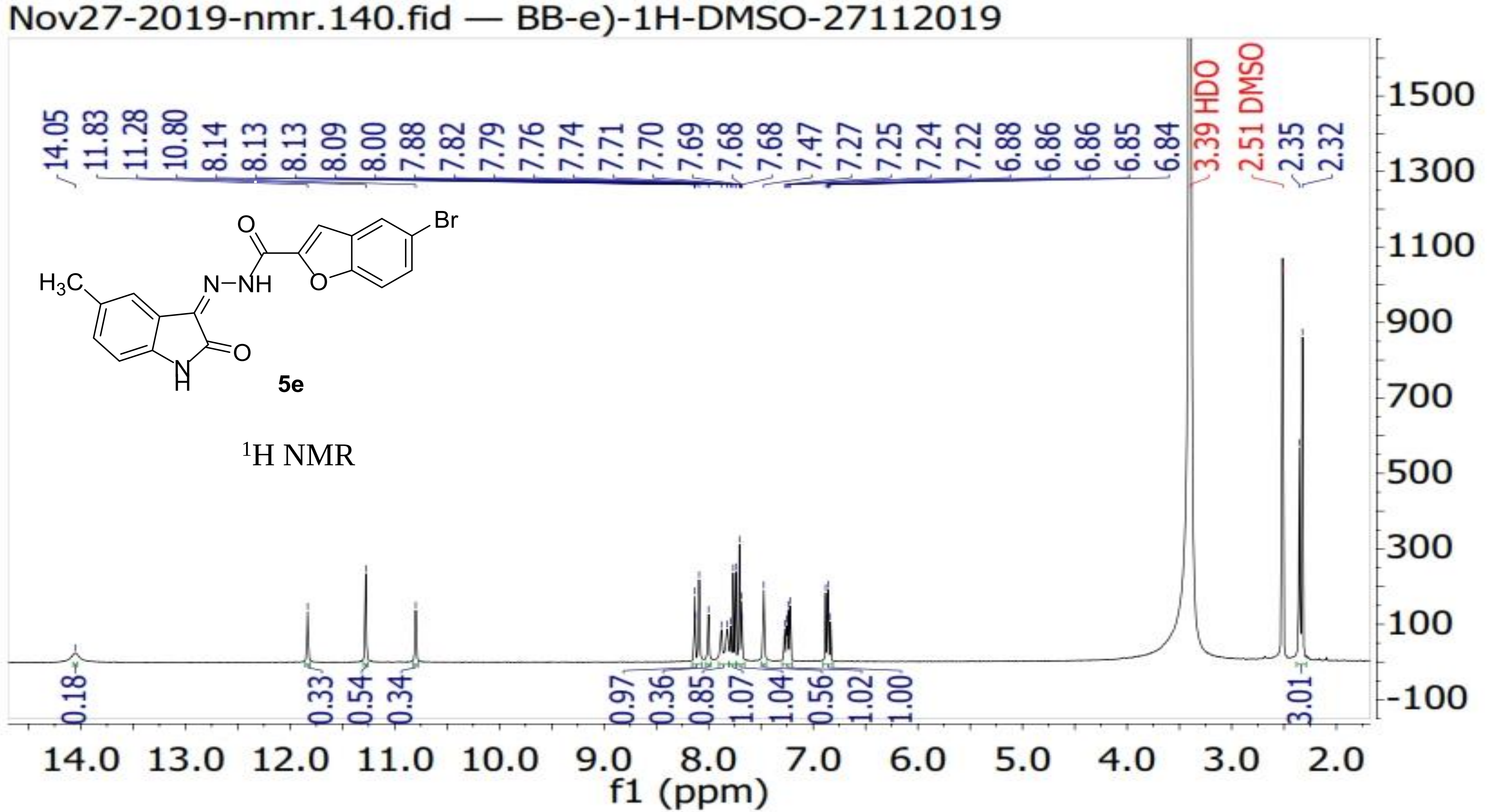

Nov27-2019-nmr.140.fid — BB-e)-1H-DMSO-27112019
14.05
11.83
11.28
10.80
8.14
8.13
8.13
8.09
8.00
7.88
7.82
7.79
7.76
7.74
7.71
7.70
7.69
7.68
7.68
7.47
7.27
7.25
7.24
7.22
6.88
6.86
6.86
6.85
6.84
3.39 HDO
2.51 DMSO
2.35
2.32
Br
O
O
H3C
N
NH
O
N
H
O
5e
1H NMR
0.18
0.33
0.54
0.34
0.97
0.36
0.85
1.07
1.04
0.56
1.02
1.00
3.01
1500
1300
1100
900
700
500
300
100
-100
14.0
13.0
12.0
11.0
10.0
9.0
8.0
7.0
6.0
5.0
4.0
3.0
2.0
f1 (ppm)

Nov27-2019-nmr.141.fid — BB-e)-13C-DMSO-27112019

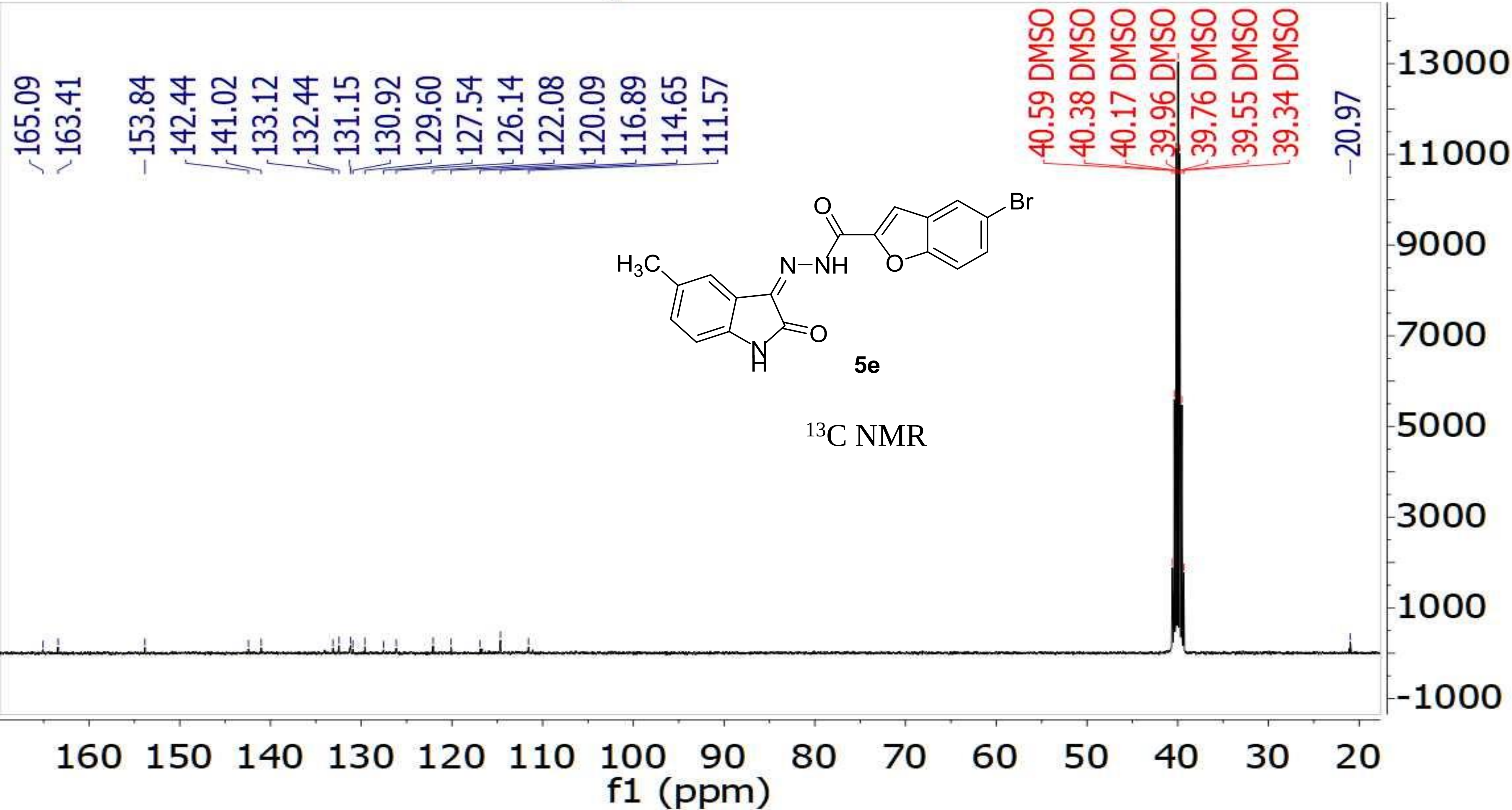

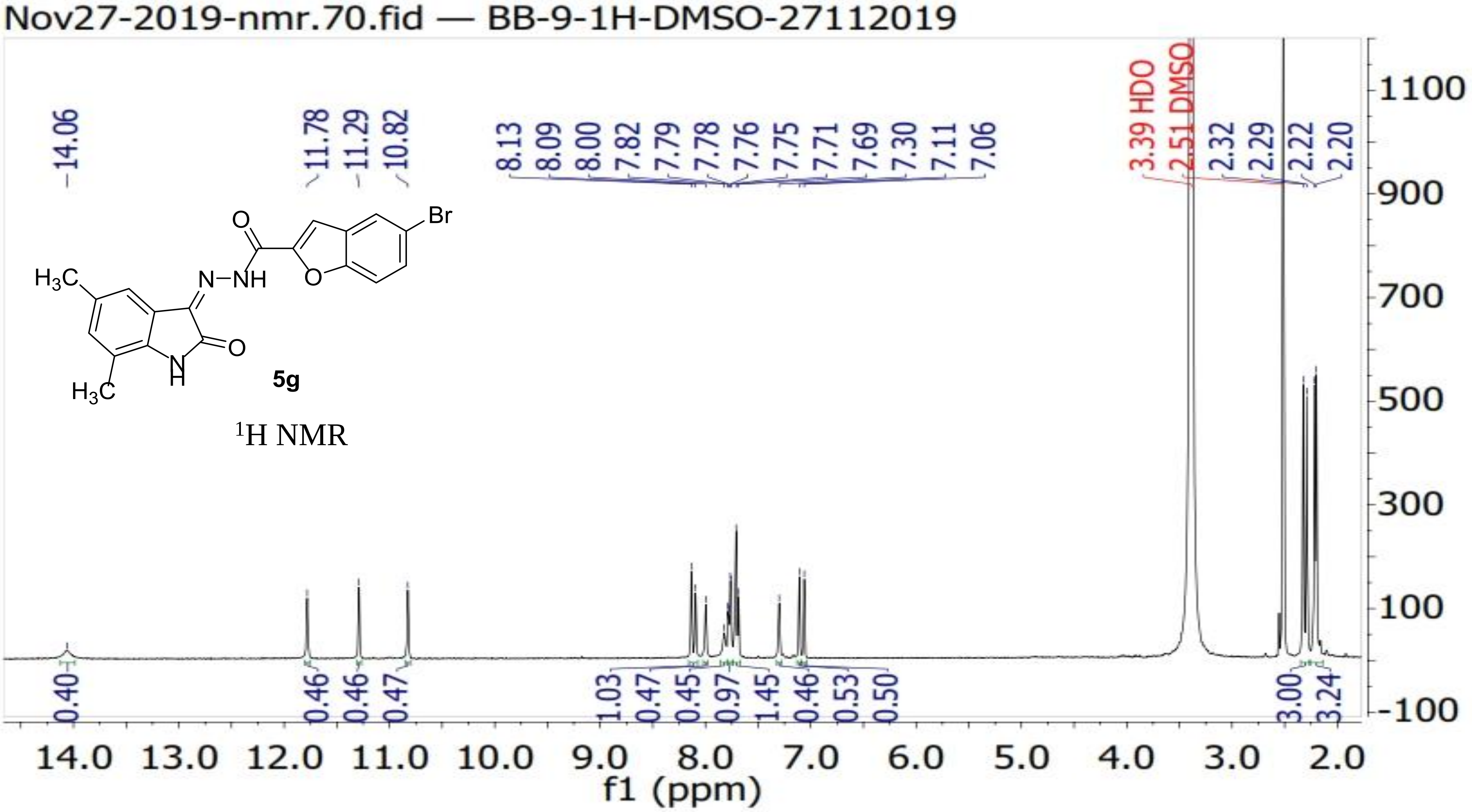
Nov27-2019-nmr.70.fid — BB-9-1H-DMSO-27112019
14.06
11.78
11.29
10.82
8.13 8.09 8.00 7.82 7.79 7.78 7.76 7.75 7.71 7.69 7.30 7.11 7.06
3.39 HDO
2.51 DMSO
2.32 2.29 2.22 2.20
Br
O
O
N–NH
H_3C
O
N
H
H_3C
5g
^{1}H NMR
0.40 0.46 0.46 0.47 1.03 0.47 0.45 0.97 1.45 0.46 0.53 0.50 3.00 3.24
1100 900 700 500 300 100 -100
14.0 13.0 12.0 11.0 10.0 9.0 8.0 7.0 6.0 5.0 4.0 3.0 2.0
f1 (ppm)

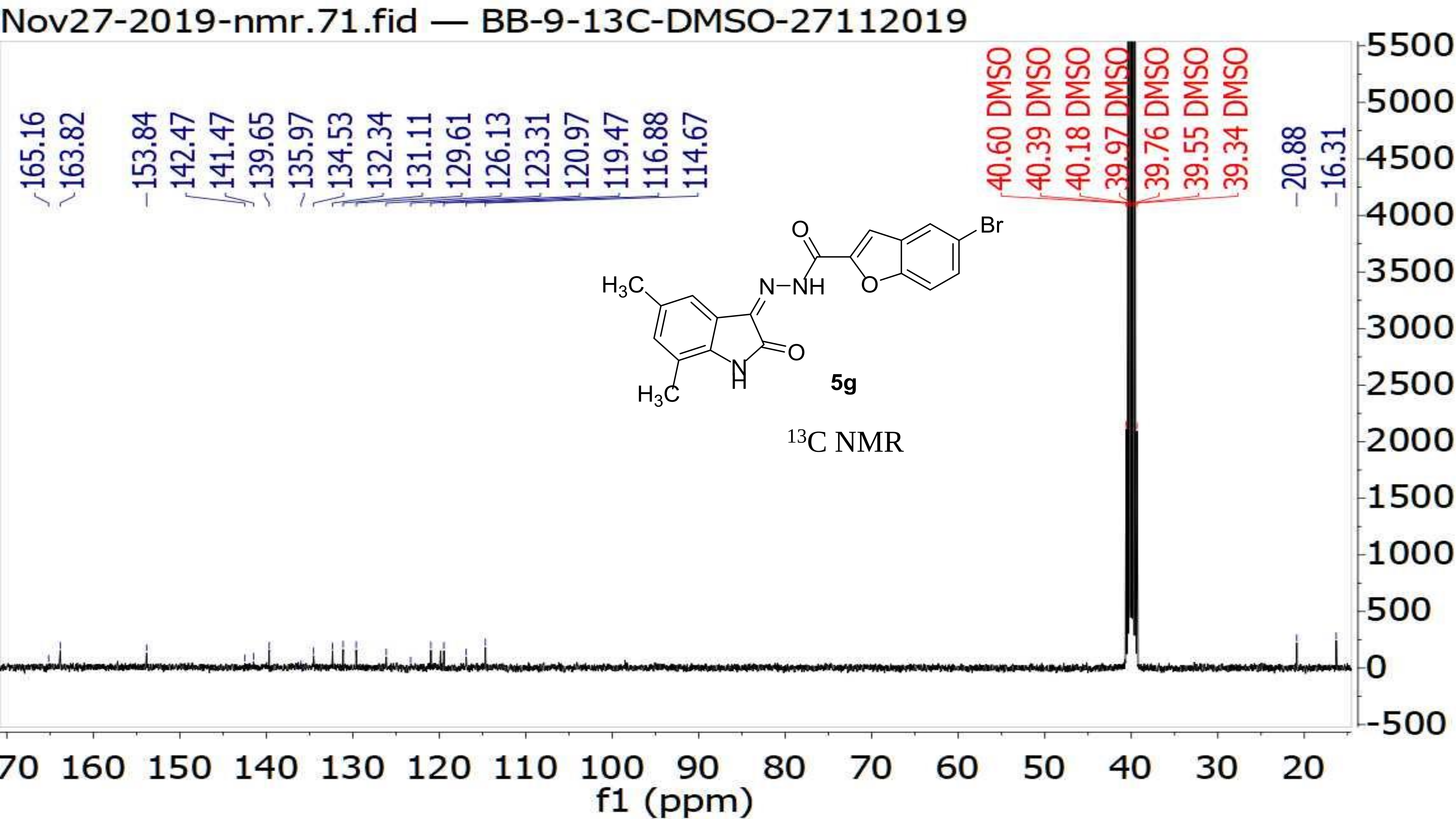
Nov27-2019-nmr.71.fid — BB-9-13C-DMSO-27112019
165.16
163.82
153.84
142.47
141.47
139.65
135.97
134.53
132.34
131.11
129.61
126.13
123.31
120.97
119.47
116.88
114.67
40.60 DMSO
40.39 DMSO
40.18 DMSO
39.97 DMSO
39.76 DMSO
39.55 DMSO
39.34 DMSO
20.88
16.31
Br
O
N–NH
O
H3C
O
N
H
H3C
5g
13C NMR
f1 (ppm)

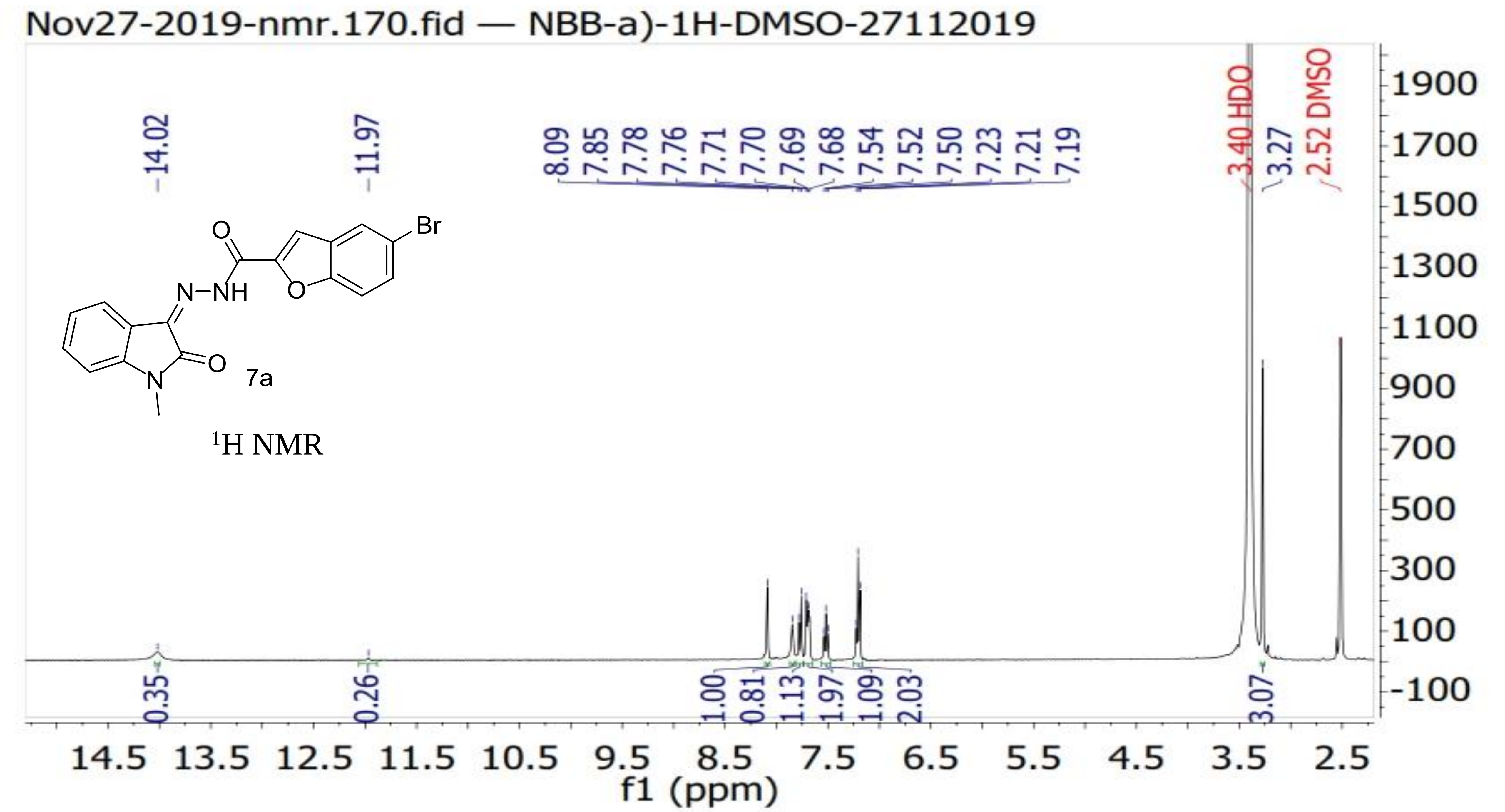

Nov27-2019-nmr.170.fid — NBB-a)-1H-DMSO-27112019
14.02
11.97
8.09 7.85 7.78 7.76 7.71 7.70 7.69 7.68 7.54 7.52 7.50 7.23 7.21 7.19
3.40 HDO
3.27
2.52 DMSO
O
Br
N–NH
O
O
N
7a
1H NMR
0.35
0.26
1.00 0.81 1.13 1.97 1.09 2.03
3.07
1900 1700 1500 1300 1100 900 700 500 300 100 -100
14.5 13.5 12.5 11.5 10.5 9.5 8.5 7.5 6.5 5.5 4.5 3.5 2.5
f1 (ppm)

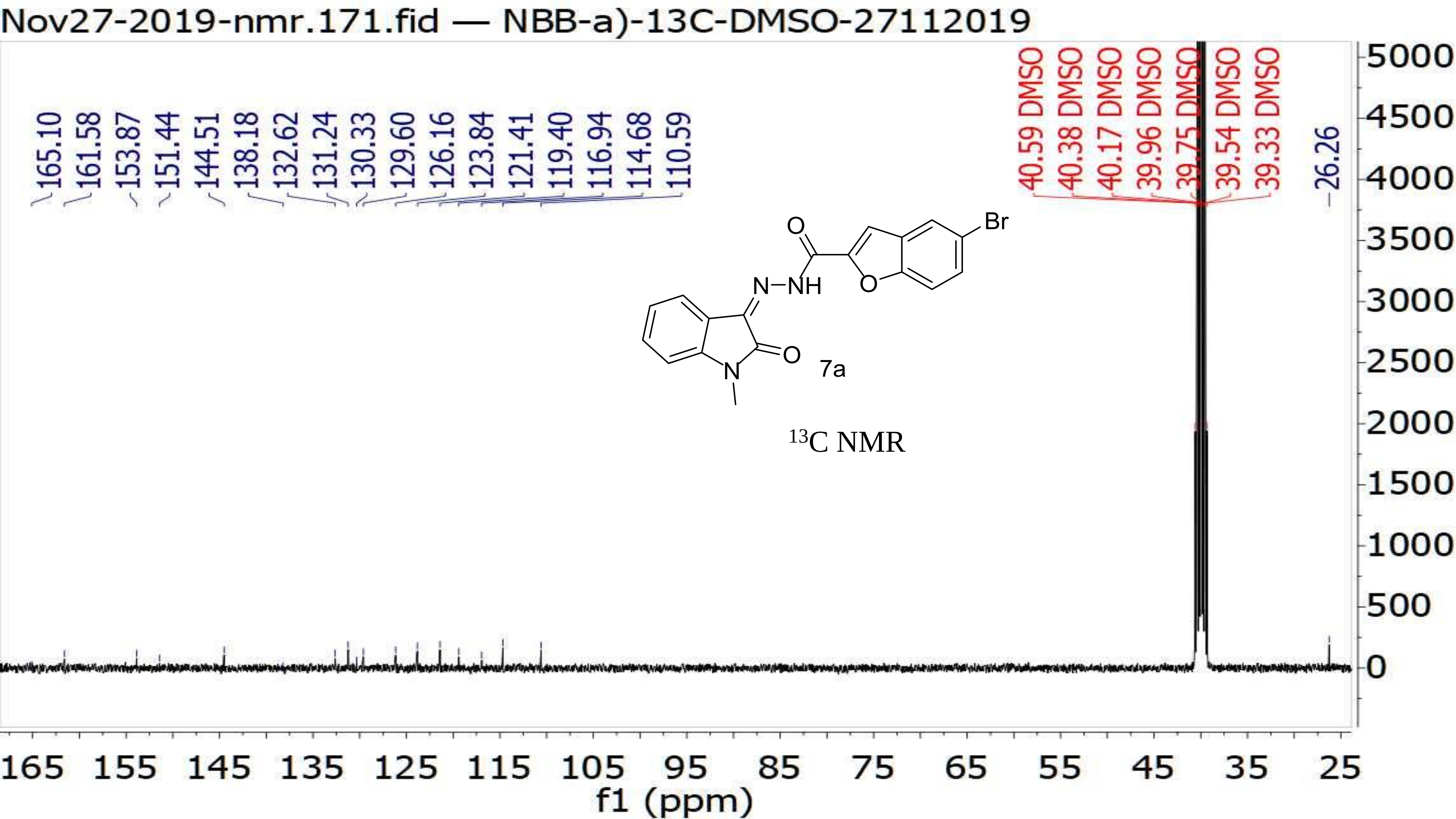

Nov27-2019-nmr.171.fid — NBB-a)-13C-DMSO-27112019
165.10
161.58
153.87
151.44
144.51
138.18
132.62
131.24
130.33
129.60
126.16
123.84
121.41
119.40
116.94
114.68
110.59
40.59 DMSO
40.38 DMSO
40.17 DMSO
39.96 DMSO
39.75 DMSO
39.54 DMSO
39.33 DMSO
26.26
O
Br
N
NH
O
O
N
7a
13C NMR
f1 (ppm)
165 155 145 135 125 115 105 95 85 75 65 55 45 35 25
5000 4500 4000 3500 3000 2500 2000 1500 1000 500 0

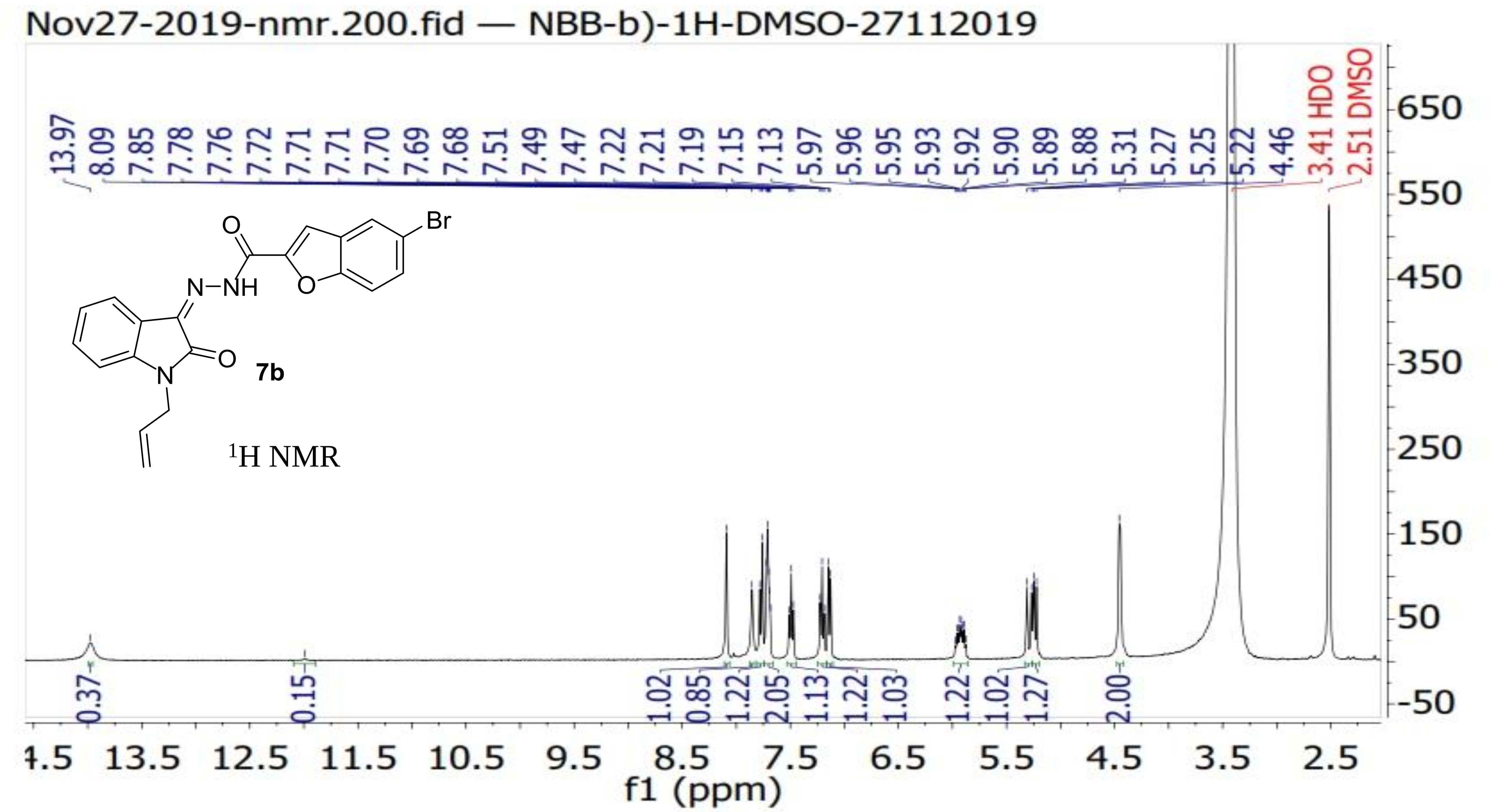

Nov27-2019-nmr.200.fid — NBB-b)-1H-DMSO-27112019
13.97
8.09
7.85
7.78
7.76
7.72
7.71
7.71
7.70
7.69
7.68
7.51
7.49
7.47
7.22
7.21
7.19
7.15
7.13
5.97
5.96
5.95
5.93
5.92
5.90
5.89
5.88
5.31
5.27
5.25
5.22
4.46
3.41 HDO
2.51 DMSO
O
Br
N–NH
O
O
N
7b
¹H NMR
0.37
0.15
1.02
0.85
1.22
2.05
1.13
1.22
1.03
1.22
1.02
1.27
2.00
650
550
450
350
250
150
50
-50
4.5
13.5
12.5
11.5
10.5
9.5
8.5
7.5
6.5
5.5
4.5
3.5
2.5
f1 (ppm)

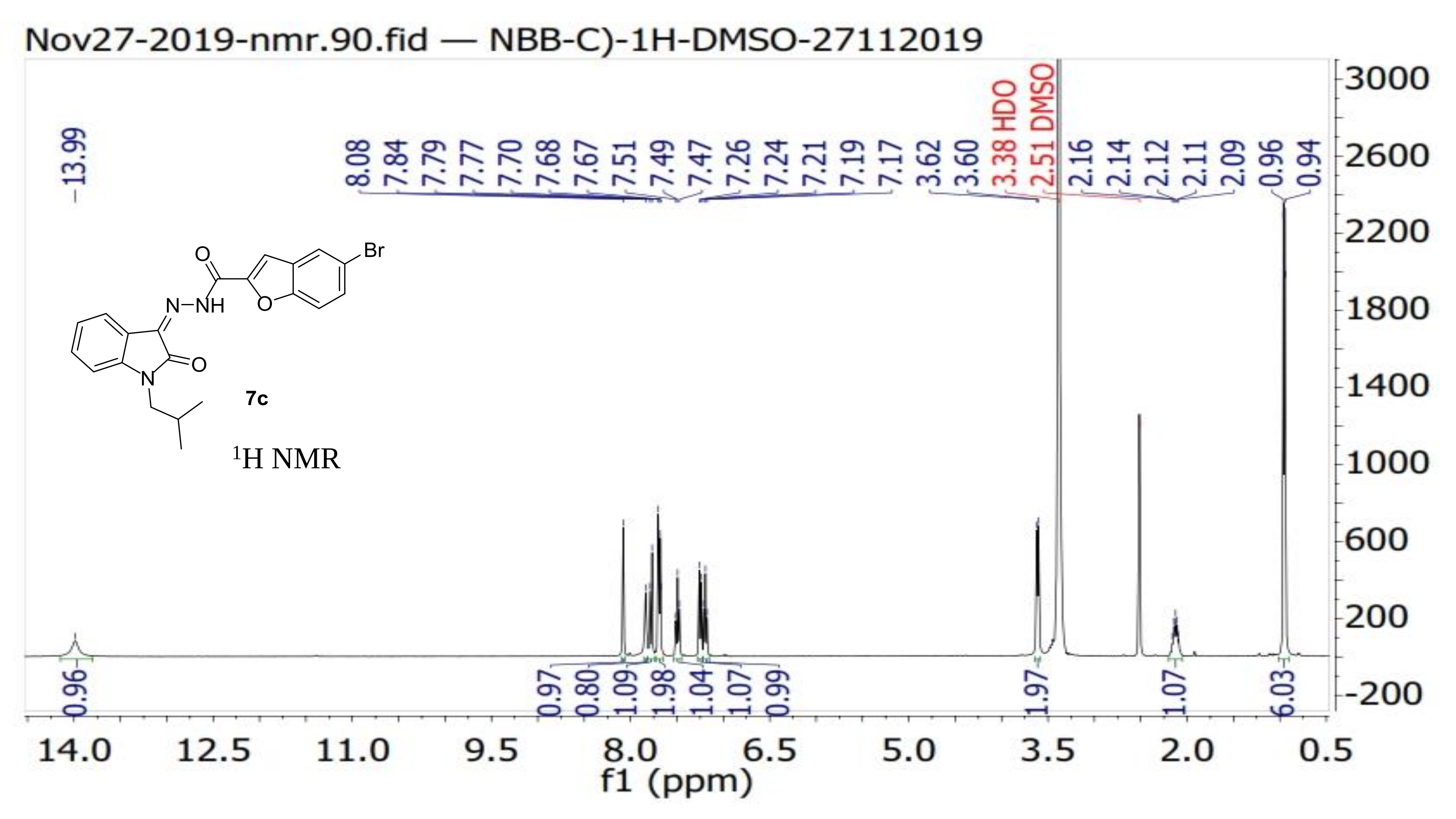
Nov27-2019-nmr.90.fid — NBB-C)-1H-DMSO-27112019
13.99
8.08 7.84 7.79 7.77 7.70 7.68 7.67 7.51 7.49 7.47 7.26 7.24 7.21 7.19 7.17 3.62 3.60 3.38 HDO 2.51 DMSO 2.16 2.14 2.12 2.11 2.09 0.96 0.94
Br
O
N–NH
O
O
N
7c
1H NMR
0.96
0.97 0.80 1.09 1.98 1.04 1.07 0.99
1.97
1.07
6.03
3000
2600
2200
1800
1400
1000
600
200
-200
14.0 12.5 11.0 9.5 8.0 6.5 5.0 3.5 2.0 0.5
f1 (ppm)

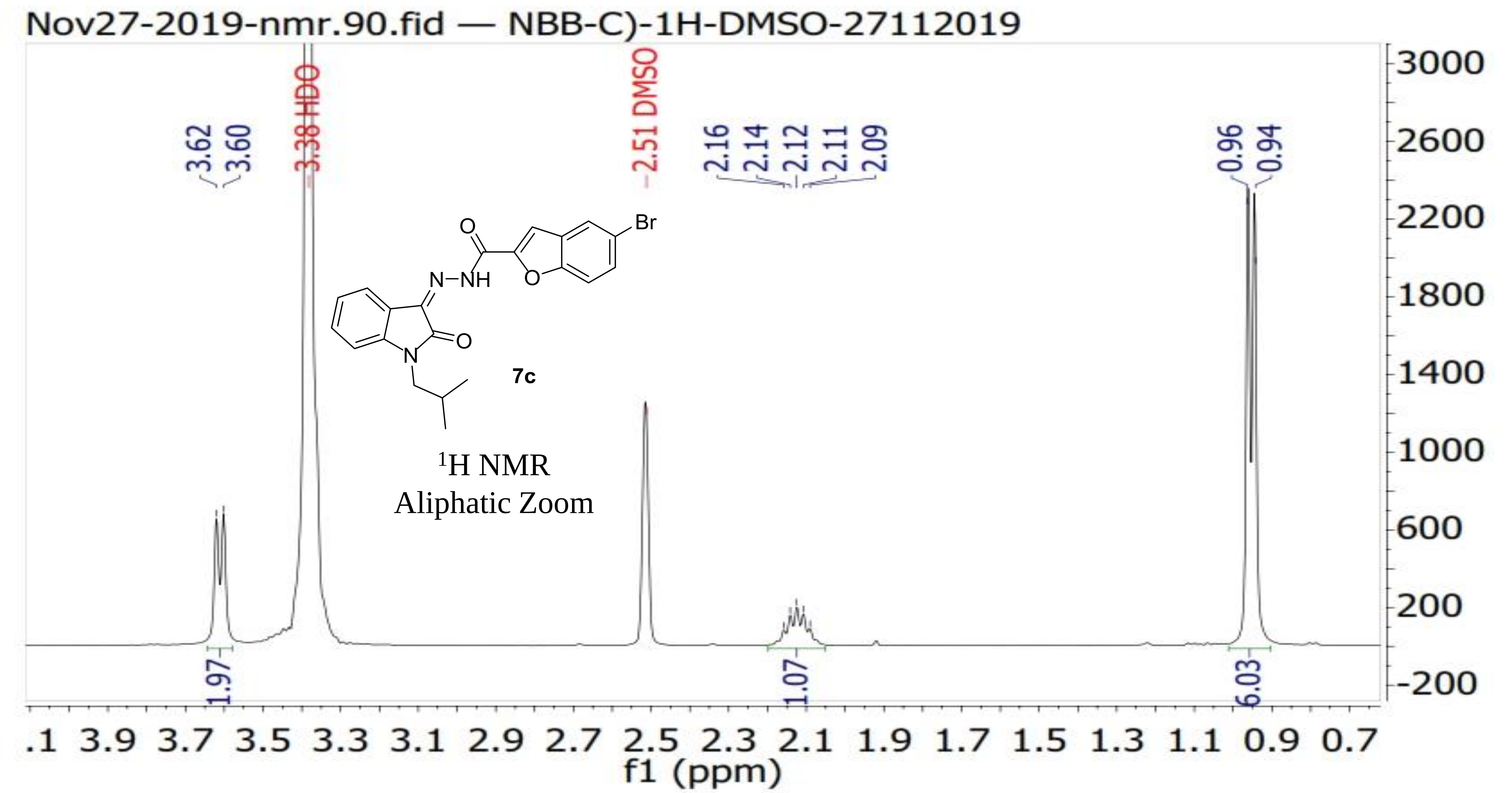

Nov27-2019-nmr.90.fid — NBB-C)-1H-DMSO-27112019
3.62
3.60
3.38 HDO
2.51 DMSO
2.16
2.14
2.12
2.11
2.09
0.96
0.94
O
Br
N–NH
O
N
O
7c
1H NMR
Aliphatic Zoom
1.97
1.07
6.03
3000
2600
2200
1800
1400
1000
600
200
-200
.1 3.9 3.7 3.5 3.3 3.1 2.9 2.7 2.5 2.3 2.1 1.9 1.7 1.5 1.3 1.1 0.9 0.7
f1 (ppm)

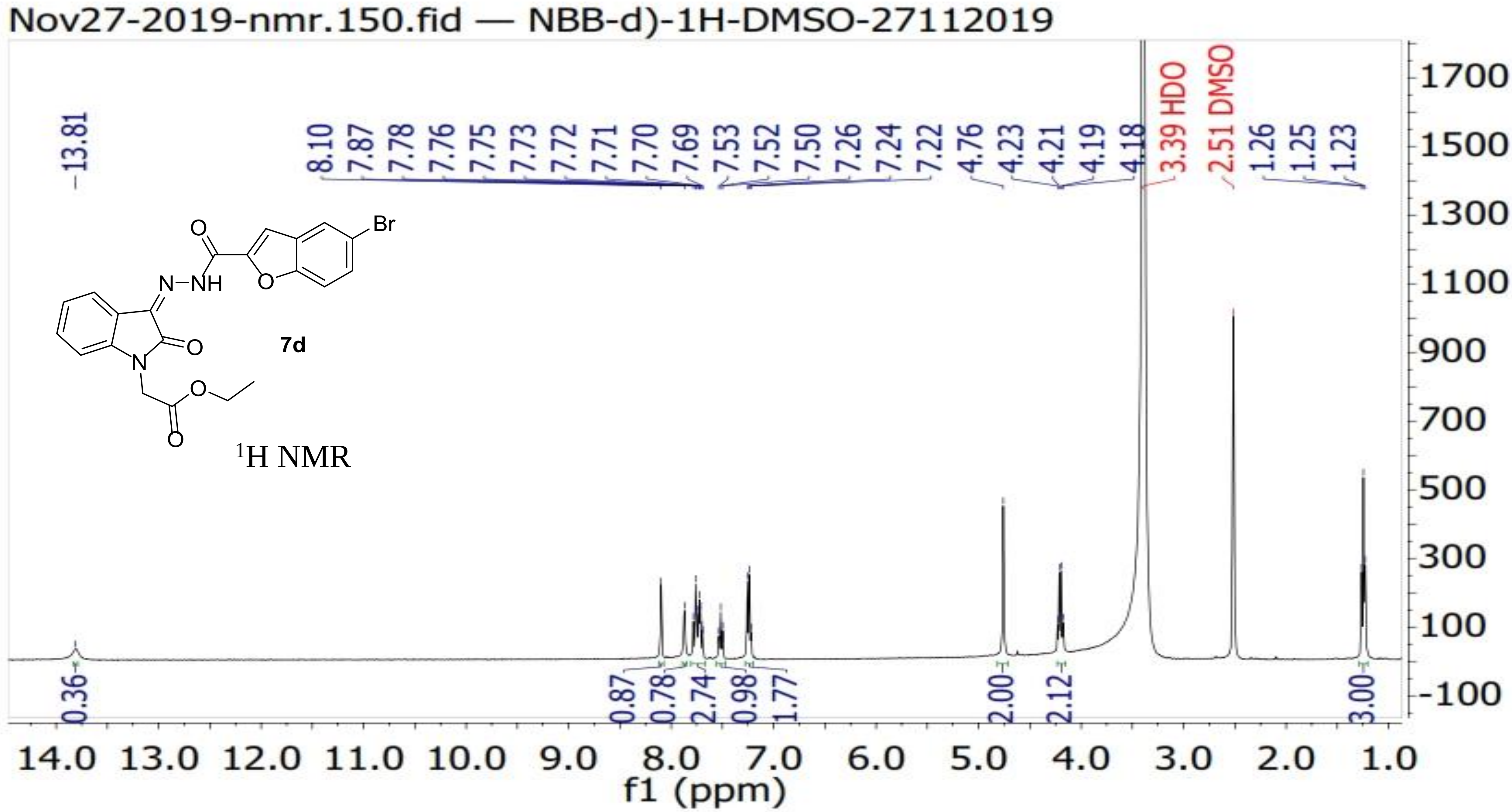

Nov27-2019-nmr.150.fid — NBB-d)-1H-DMSO-27112019
13.81
8.10 7.87 7.78 7.76 7.75 7.73 7.72 7.71 7.70 7.69 7.53 7.52 7.50 7.26 7.24 7.22 4.76 4.23 4.21 4.19 4.18
3.39 HDO
2.51 DMSO
1.26 1.25 1.23
Br
O
N–NH
O
O
N
O
O
7d
1H NMR
0.36
0.87 0.78 2.74 0.98 1.77
2.00 2.12
3.00
1700 1500 1300 1100 900 700 500 300 100 -100
14.0 13.0 12.0 11.0 10.0 9.0 8.0 7.0 6.0 5.0 4.0 3.0 2.0 1.0
f1 (ppm)

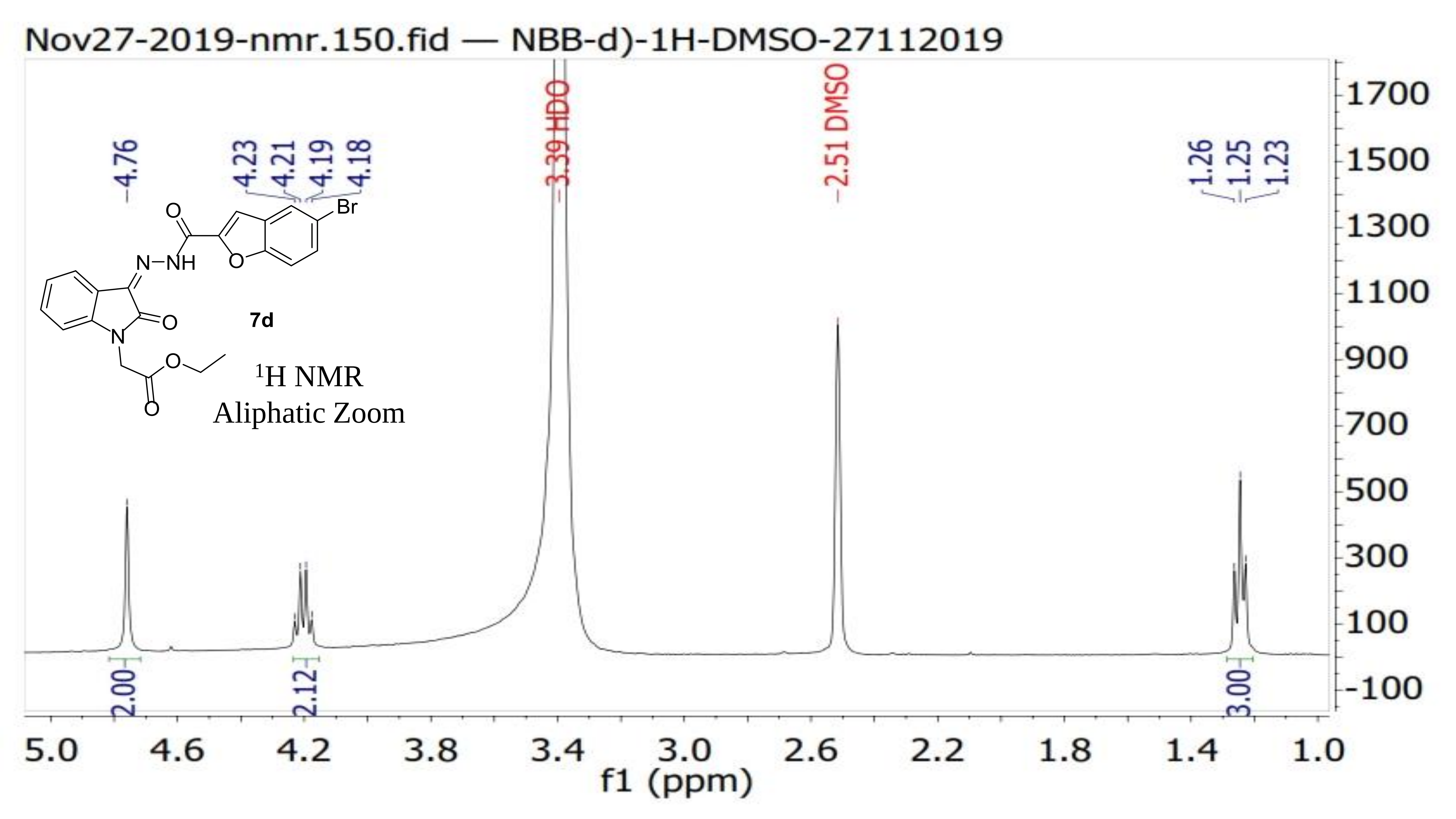

Nov27-2019-nmr.150.fid — NBB-d)-1H-DMSO-27112019
4.76
4.23
4.21
4.19
4.18
3.39 HDO
2.51 DMSO
1.26
1.25
1.23
O
Br
N
NH
O
O
N
O
O
O
7d
1H NMR
Aliphatic Zoom
2.00
2.12
3.00
1700
1500
1300
1100
900
700
500
300
100
-100
5.0
4.6
4.2
3.8
3.4
3.0
2.6
2.2
1.8
1.4
1.0
f1 (ppm)

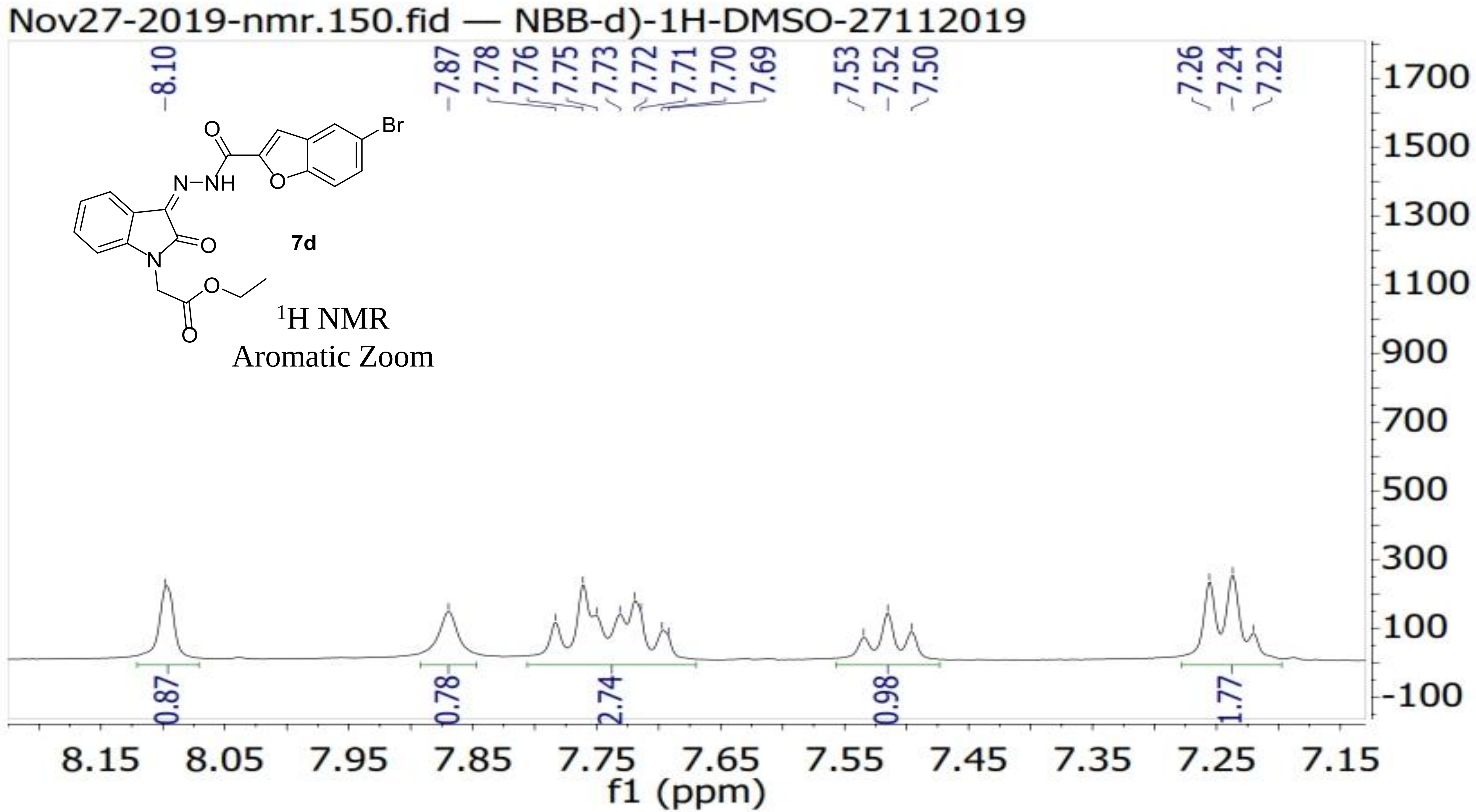
Nov27-2019-nmr.150.fid — NBB-d)-1H-DMSO-27112019
8.10
7.87
7.78
7.76
7.75
7.73
7.72
7.71
7.70
7.69
7.53
7.52
7.50
7.26
7.24
7.22
Br
O
N–NH
O
O
N
O
O
7d
1H NMR
Aromatic Zoom
0.87
0.78
2.74
0.98
1.77
1700
1500
1300
1100
900
700
500
300
100
-100
8.15
8.05
7.95
7.85
7.75
7.65
7.55
7.45
7.35
7.25
7.15
f1 (ppm)

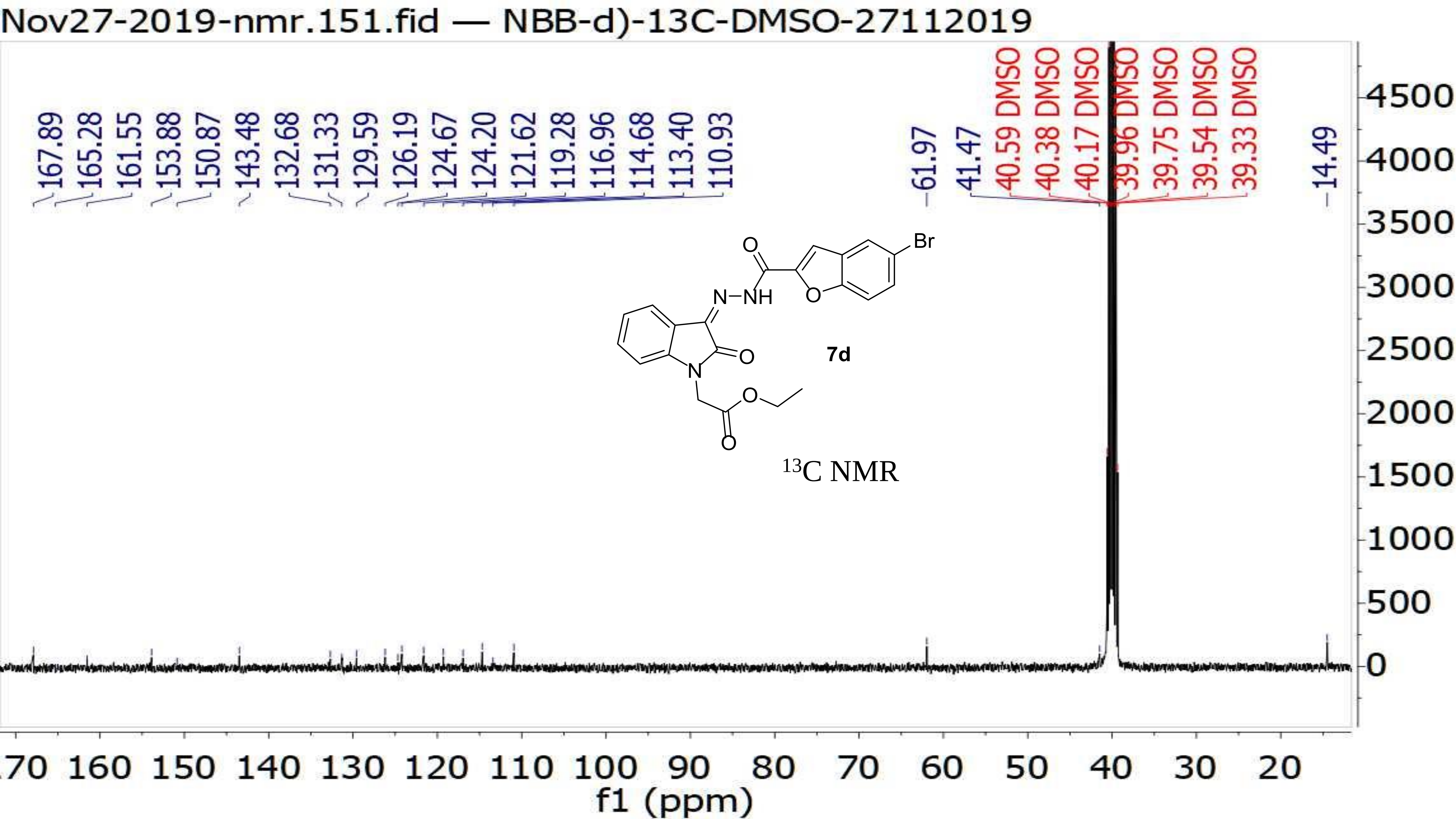
Nov27-2019-nmr.151.fid — NBB-d)-13C-DMSO-27112019
167.89
165.28
161.55
153.88
150.87
143.48
132.68
131.33
129.59
126.19
124.67
124.20
121.62
119.28
116.96
114.68
113.40
110.93
61.97
41.47
40.59 DMSO
40.38 DMSO
40.17 DMSO
39.96 DMSO
39.75 DMSO
39.54 DMSO
39.33 DMSO
14.49
Br
O
N
NH
O
O
N
O
O
O
7d
13C NMR
f1 (ppm)

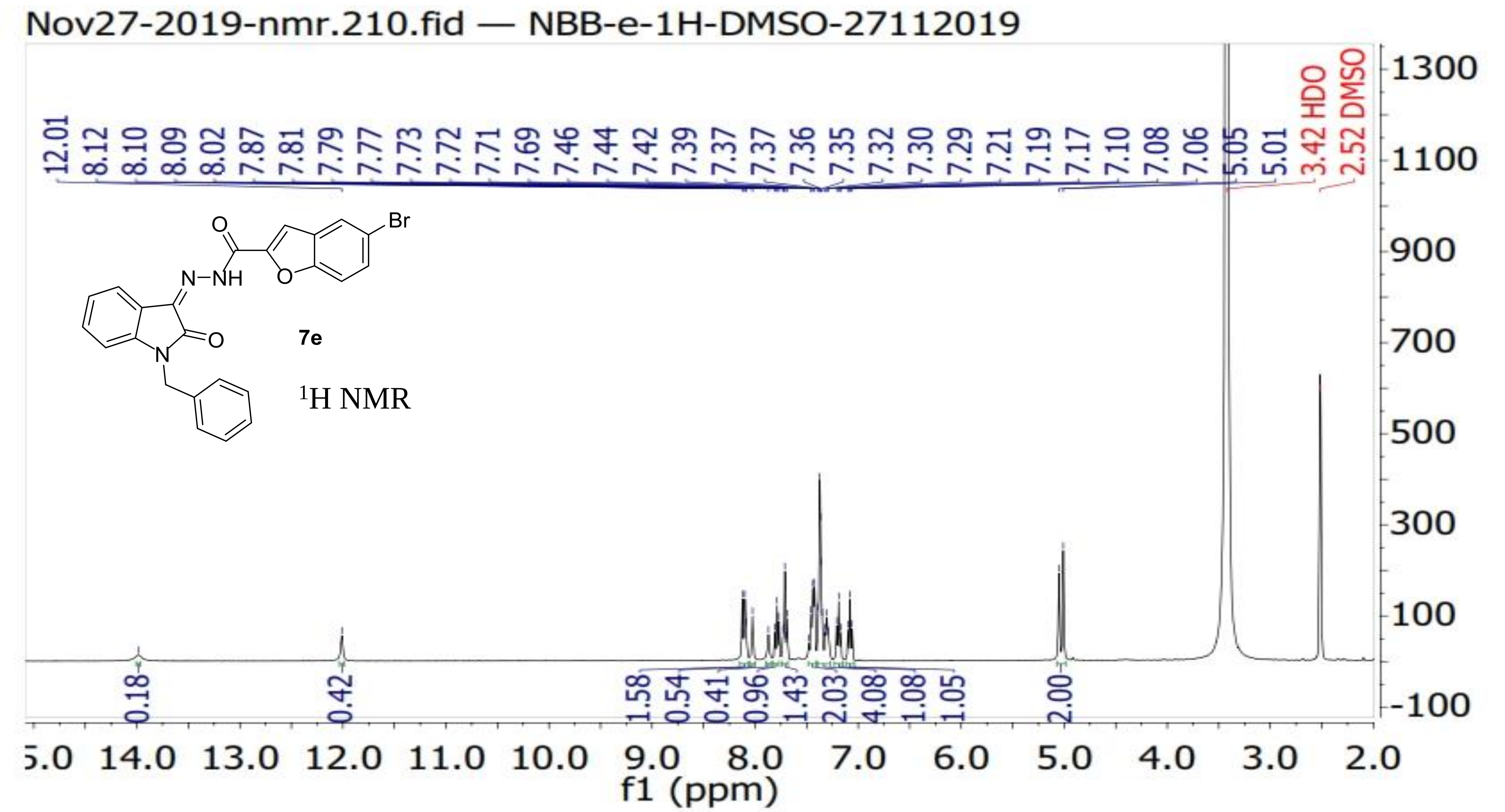

Nov27-2019-nmr.210.fid — NBB-e-1H-DMSO-27112019
12.01
8.12
8.10
8.09
8.02
7.87
7.81
7.79
7.77
7.73
7.72
7.71
7.69
7.46
7.44
7.42
7.39
7.37
7.37
7.36
7.35
7.32
7.30
7.29
7.21
7.19
7.17
7.10
7.08
7.06
5.05
5.01
3.42 HDO
2.52 DMSO
Br
O
N
NH
O
O
N
7e
1H NMR
0.18
0.42
1.58
0.54
0.41
0.96
1.43
2.03
4.08
1.08
1.05
2.00
1300
1100
900
700
500
300
100
-100
5.0
14.0
13.0
12.0
11.0
10.0
9.0
8.0
7.0
6.0
5.0
4.0
3.0
2.0
f1 (ppm)

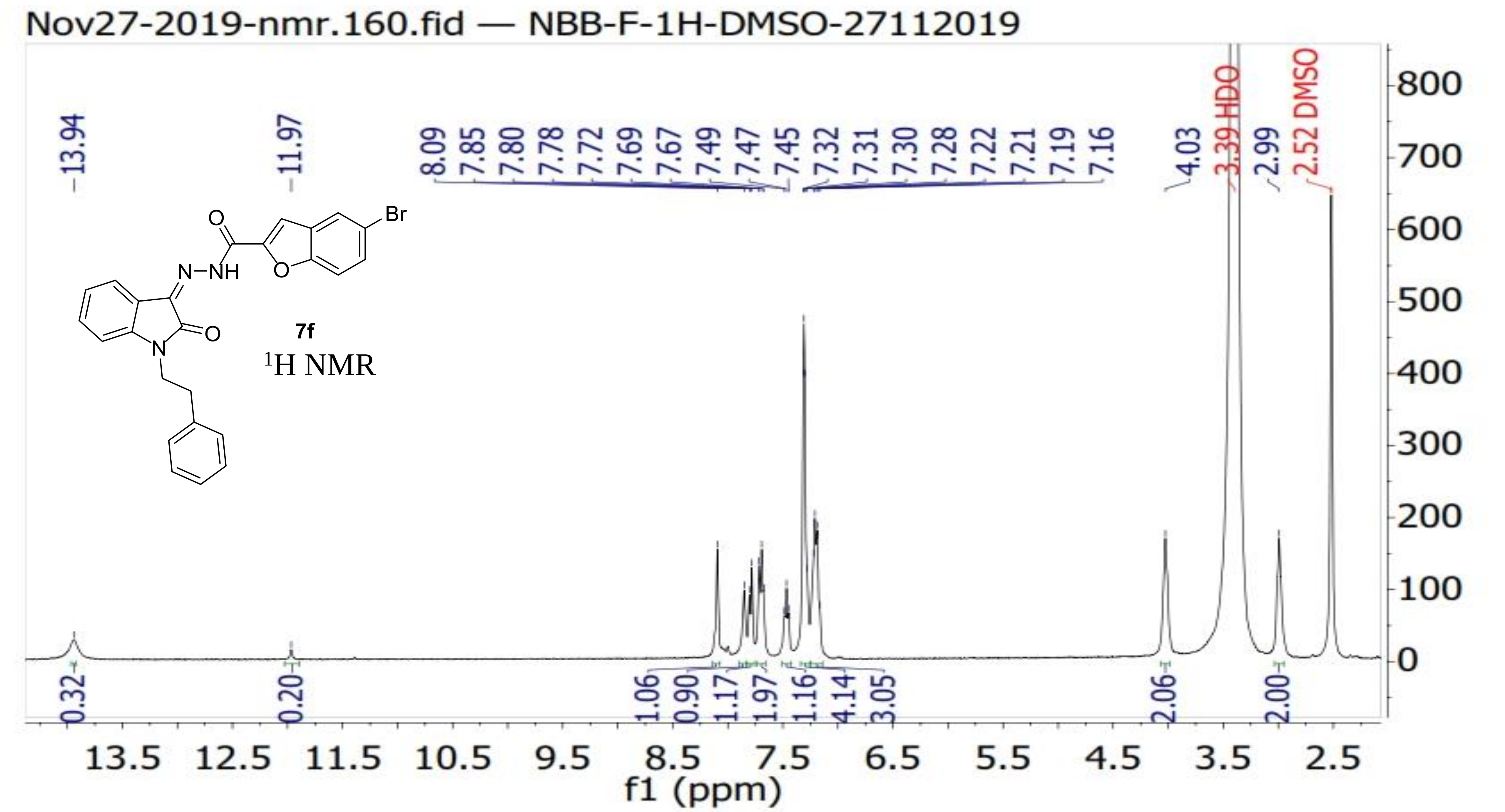
Nov27-2019-nmr.160.fid — NBB-F-1H-DMSO-27112019
13.94
11.97
8.09 7.85 7.80 7.78 7.72 7.69 7.67 7.49 7.47 7.45 7.32 7.31 7.30 7.28 7.22 7.21 7.19 7.16
4.03
3.39 HDO
2.99
2.52 DMSO
O
Br
N
NH
O
O
N
7f
1H NMR
0.32
0.20
1.06 0.90 1.17 1.97 1.16 4.14 3.05
2.06
2.00
800
700
600
500
400
300
200
100
0
13.5 12.5 11.5 10.5 9.5 8.5 7.5 6.5 5.5 4.5 3.5 2.5
f1 (ppm)

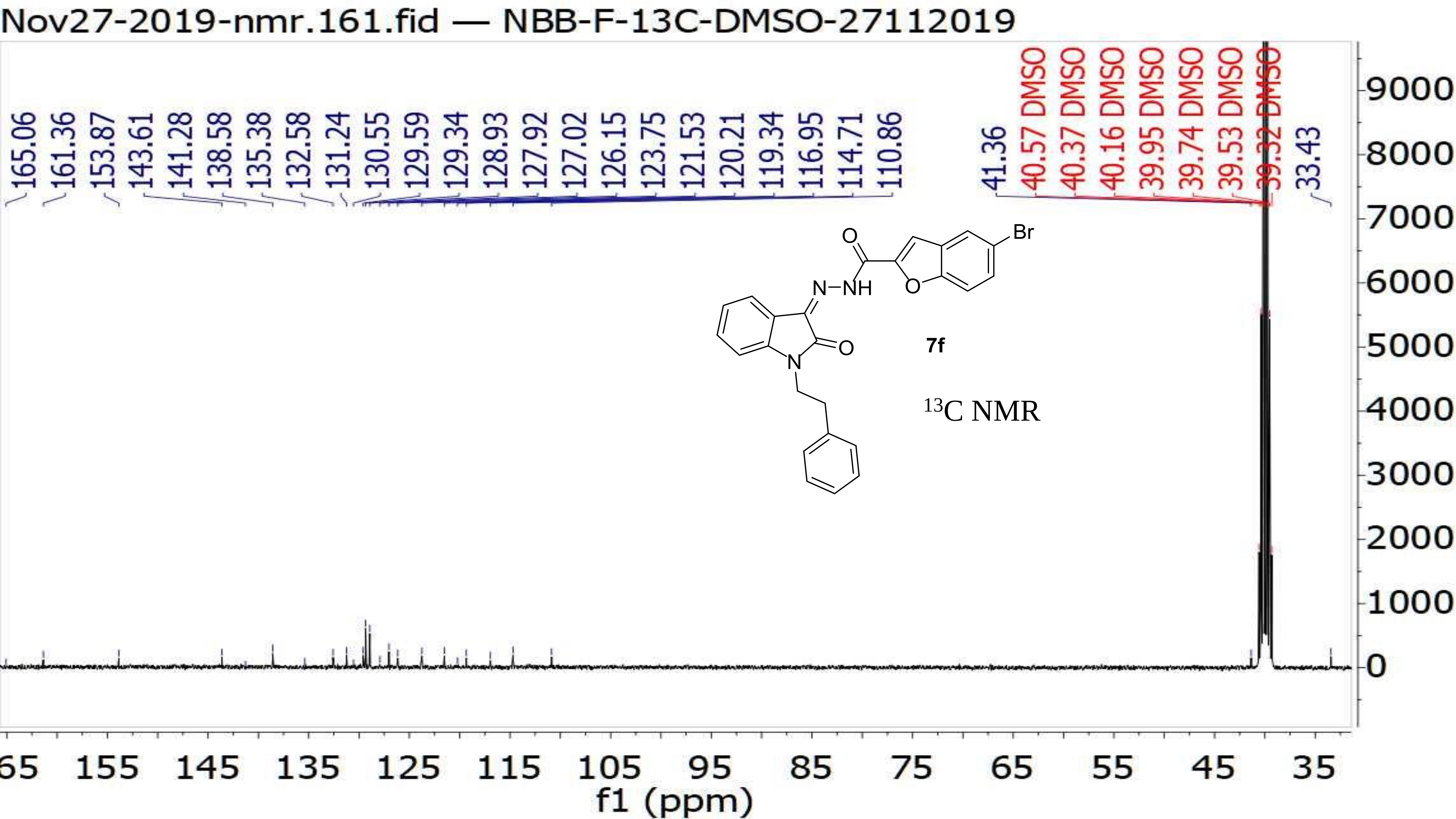
Nov27-2019-nmr.161.fid — NBB-F-13C-DMSO-27112019
165.06
161.36
153.87
143.61
141.28
138.58
135.38
132.58
131.24
130.55
129.59
129.34
128.93
127.92
127.02
126.15
123.75
121.53
120.21
119.34
116.95
114.71
110.86
41.36
40.57 DMSO
40.37 DMSO
40.16 DMSO
39.95 DMSO
39.74 DMSO
39.53 DMSO
39.32 DMSO
33.43
O
Br
N–NH
O
O
N
7f
13C NMR
9000
8000
7000
6000
5000
4000
3000
2000
1000
0
65
155
145
135
125
115
105
95
85
75
65
55
45
35
f1 (ppm)

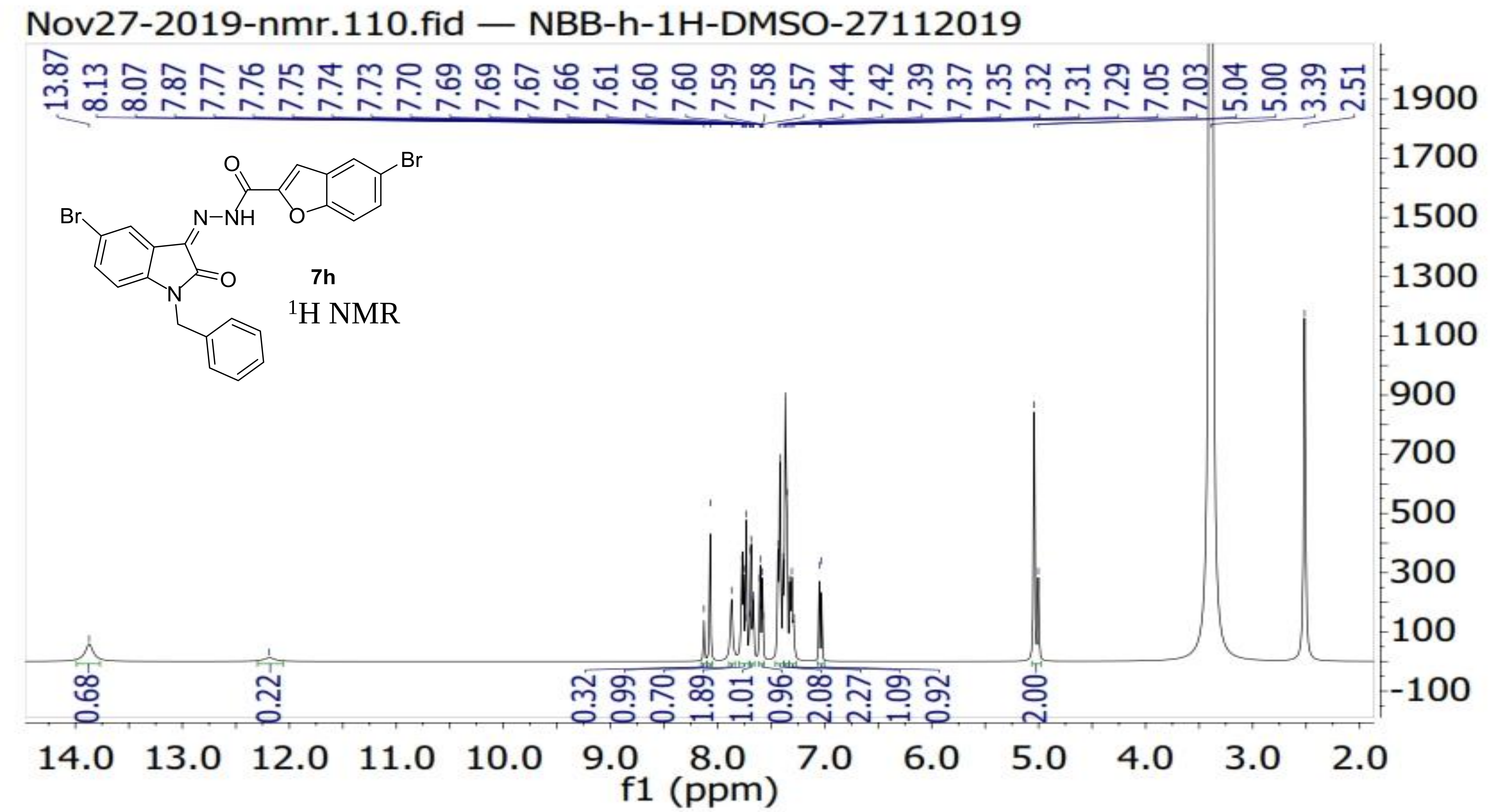
Nov27-2019-nmr.110.fid — NBB-h-1H-DMSO-27112019
13.87 8.13 8.07 7.87 7.77 7.76 7.75 7.74 7.73 7.70 7.69 7.69 7.67 7.66 7.61 7.60 7.60 7.59 7.58 7.57 7.44 7.42 7.39 7.37 7.35 7.32 7.31 7.29 7.05 7.03 5.04 5.00 3.39 2.51
Br
O
N–NH
O
O
N
Br
7h
^{1}H NMR
0.68 0.22 0.32 0.99 0.70 1.89 1.01 0.96 2.08 2.27 1.09 0.92 2.00
14.0 13.0 12.0 11.0 10.0 9.0 8.0 7.0 6.0 5.0 4.0 3.0 2.0
f1 (ppm)
1900 1700 1500 1300 1100 900 700 500 300 100 -100

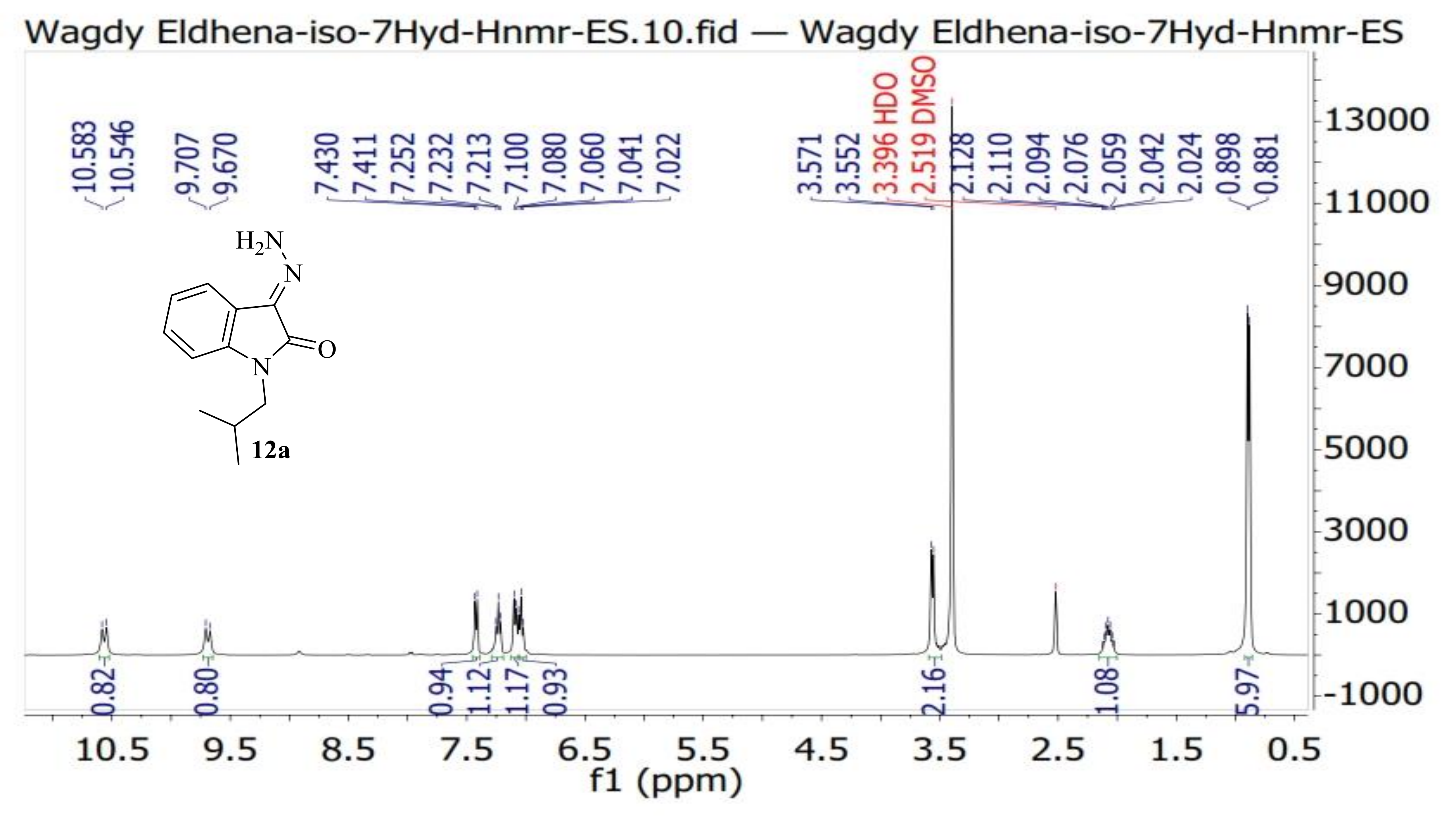

Wagdy Eldhena-iso-7Hyd-Hnmr-ES.10.fid — Wagdy Eldhena-iso-7Hyd-Hnmr-ES
10.583
10.546
9.707
9.670
7.430
7.411
7.252
7.232
7.213
7.100
7.080
7.060
7.041
7.022
3.571
3.552
3.396 HDO
2.519 DMSO
2.128
2.110
2.094
2.076
2.059
2.042
2.024
0.898
0.881
H2N
N
O
N
12a
0.82
0.80
0.94
1.12
1.17
0.93
2.16
1.08
5.97
13000
11000
9000
7000
5000
3000
1000
-1000
10.5
9.5
8.5
7.5
6.5
5.5
4.5
3.5
2.5
1.5
0.5
f1 (ppm)

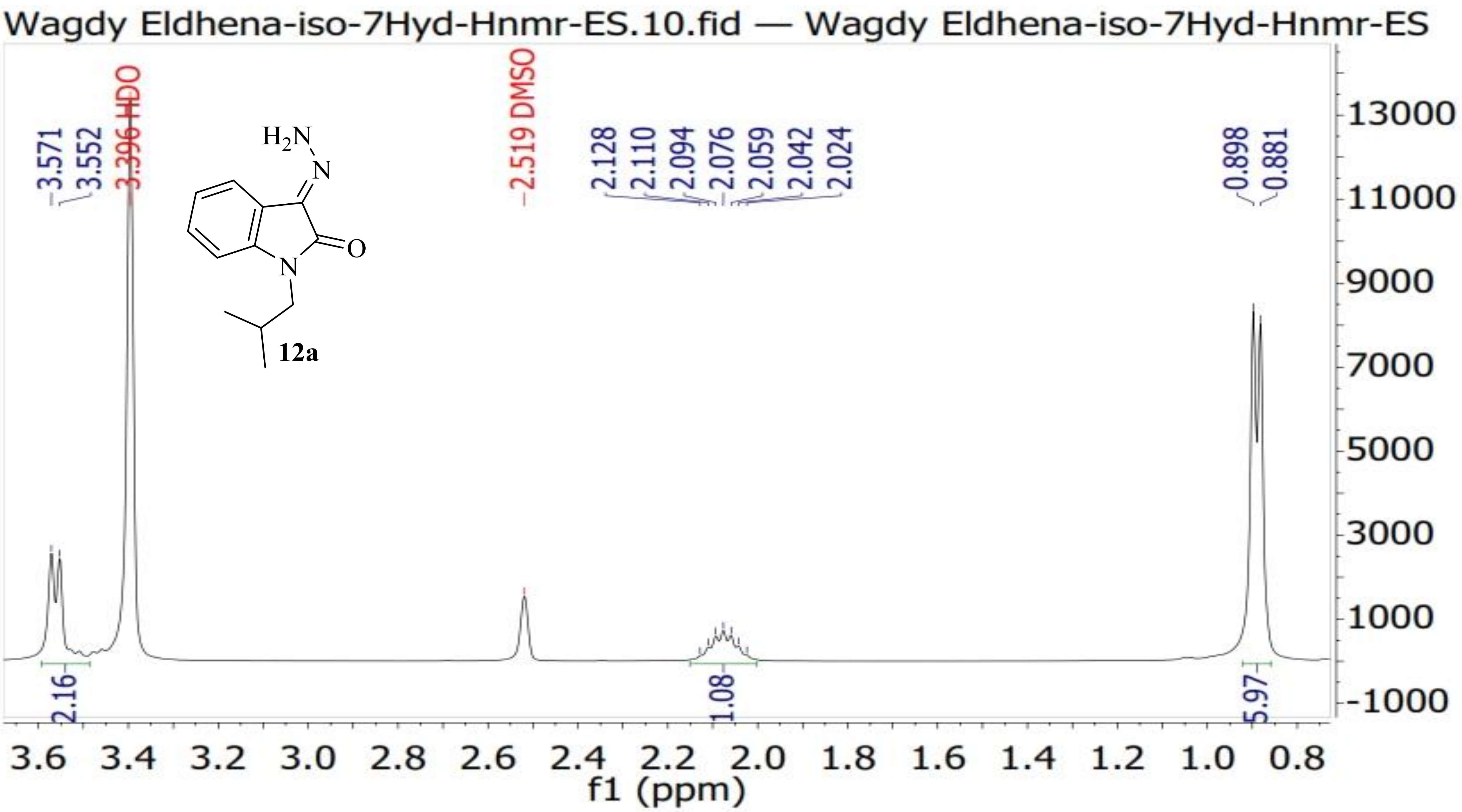

Wagdy Eldhena-iso-7Hyd-Hnmr-ES.10.fid — Wagdy Eldhena-iso-7Hyd-Hnmr-ES
3.571
3.552
3.396 HDO
2.519 DMSO
2.128
2.110
2.094
2.076
2.059
2.042
2.024
0.898
0.881
H_2N
N
N
O
12a
13000
11000
9000
7000
5000
3000
1000
-1000
2.16
1.08
5.97
3.6 3.4 3.2 3.0 2.8 2.6 2.4 2.2 2.0 1.8 1.6 1.4 1.2 1.0 0.8
f1 (ppm)

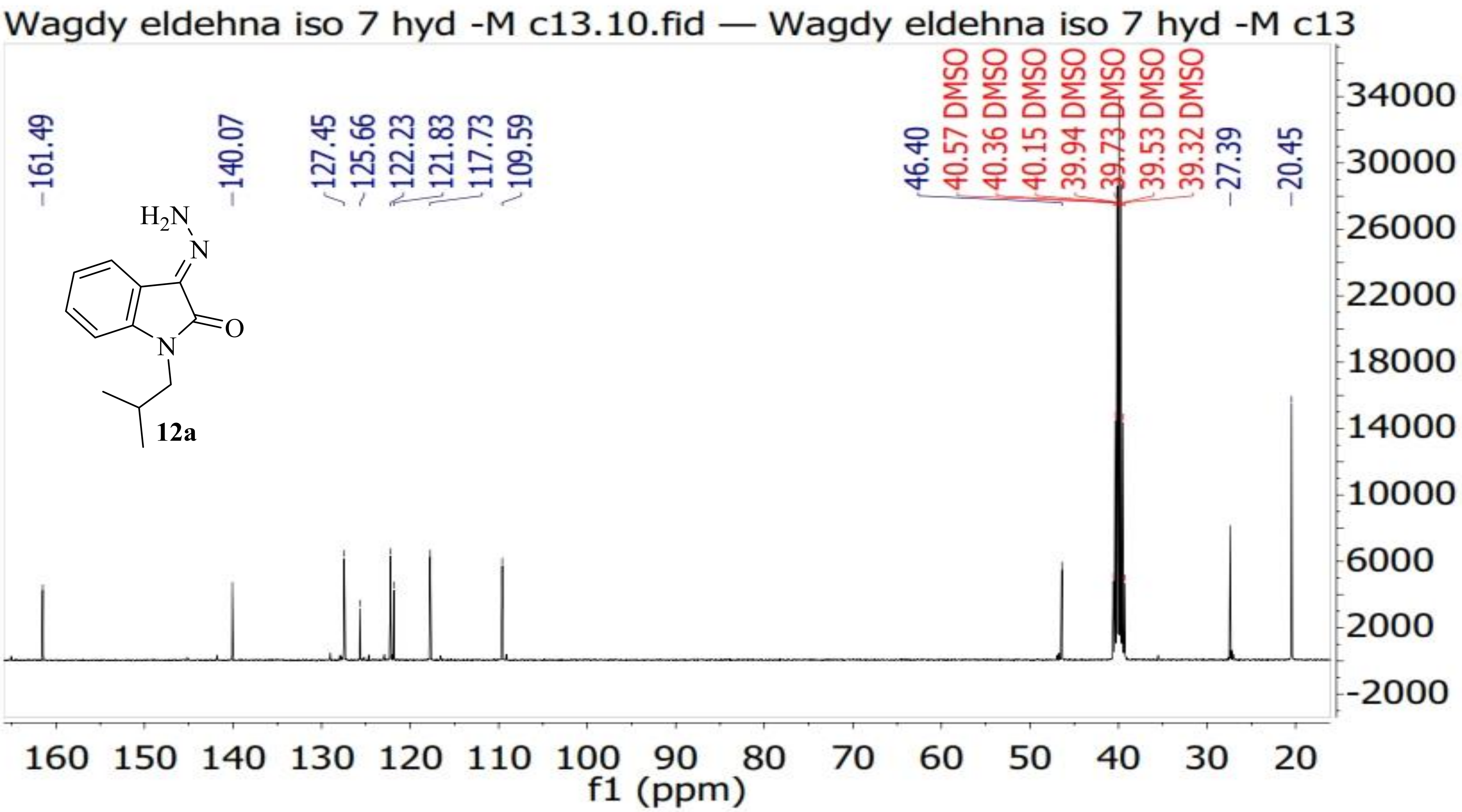
Wagdy eldehna iso 7 hyd -M c13.10.fid — Wagdy eldehna iso 7 hyd -M c13
161.49
140.07
127.45
125.66
122.23
121.83
117.73
109.59
46.40
40.57 DMSO
40.36 DMSO
40.15 DMSO
39.94 DMSO
39.73 DMSO
39.53 DMSO
39.32 DMSO
27.39
20.45
H2N
N
O
N
12a
34000
30000
26000
22000
18000
14000
10000
6000
2000
-2000
160
150
140
130
120
110
100
90
80
70
60
50
40
30
20
f1 (ppm)

Nov27-2019-nmr.61.fid — HBB-ib-13C -DMSO-27112019

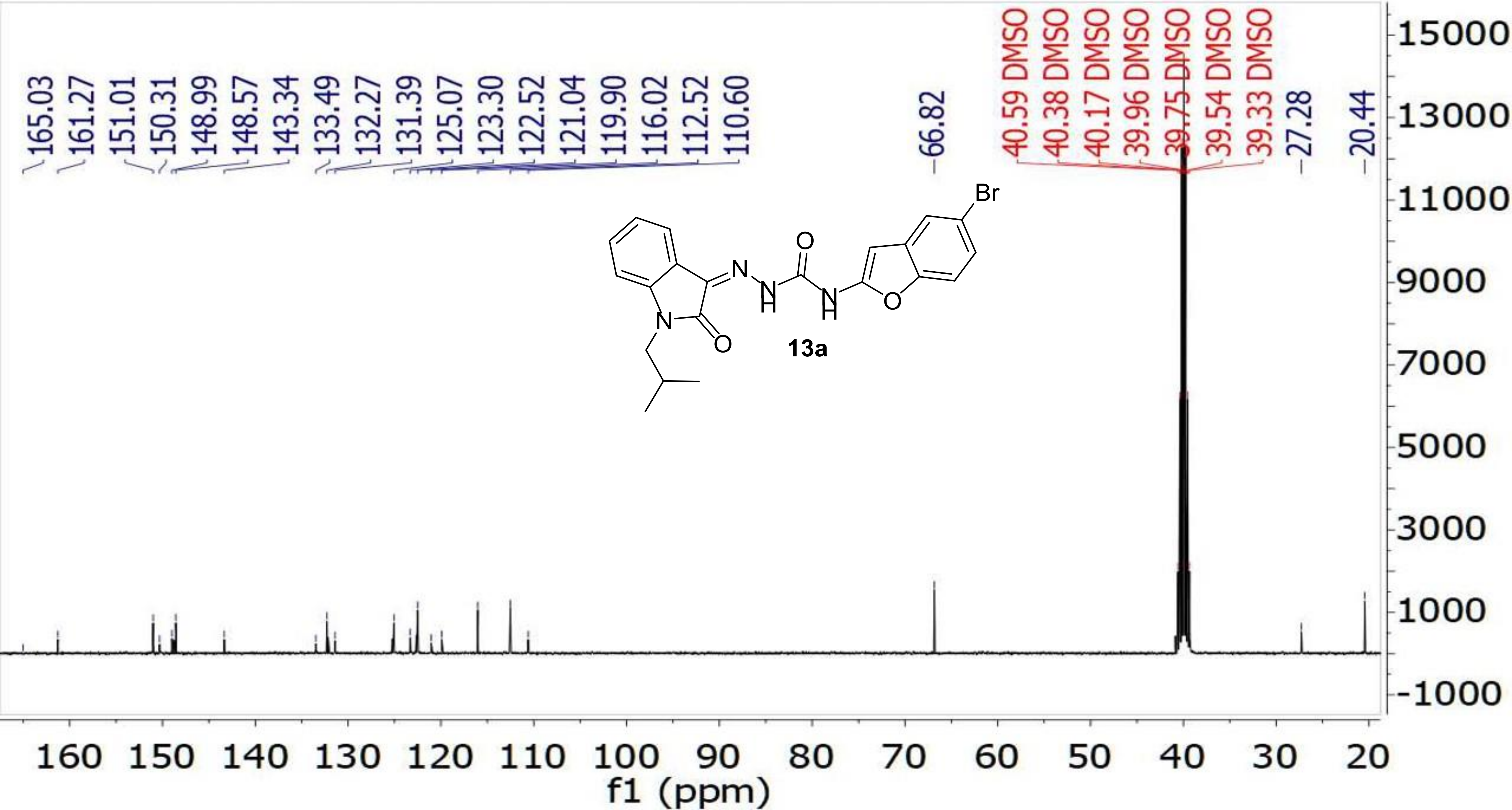

Supplement: Supplemental Material [file IENZ_A_1862101_SM8866.pdf]
